# Modeling Parkinson’s pathology in human iPSC dopaminergic neurons uncovers key mechanisms of Lewy body formation and heterogeneity

**DOI:** 10.1126/sciadv.aed8851

**Published:** 2026-07-10

**Authors:** Anne-Laure Mahul-Mellier, Lukas van den Heuvel, Maxime Teixeira, Manel L. N. Boussouf, Gaspard Oudinot, Amélie Thonet, Davide Speri, Yllza Jasiqi, Christina Ulrich, Razan Sheta, Walid Idi, Mary Croisier, Stéphanie Clerc-Rosset, Jérôme Blanc, Graham Knott, Abid Oueslati, Hilal A. Lashuel

**Affiliations:** ^1^ Laboratory of Molecular and Chemical Biology of Neurodegeneration, Institute of Bioengineering (IBI), Ecole Polytechnique Fédérale de Lausanne (EPFL), 1015 Lausanne, Switzerland.; ^2^ UPDANGELO, IBI and Global Health Institute, EPFL, 1015 Lausanne, Switzerland.; ^3^ Laboratory of Biological Electron Microscopy, Institute of Physics, School of Basic Sciences, Ecole Polytechnique Fédérale de Lausanne, 1015 Lausanne, Switzerland.; ^4^ CHU de Québec Research Center, Axe Neurosciences, Quebec City G1V 4G2, Canada.; ^5^ Department of Molecular Medicine, Faculty of Medicine, Université Laval, Quebec City G1V 0A6, Canada.; ^6^ BioEM Core Facility and Technology Platform, EPFL, 1015 Lausanne, Switzerland.; ^7^ Weill Cornell Medicine Qatar, Education City, Qatar Foundation, Doha, Qatar.; ^8^ Department of Neurology, Weill Cornell Medicine, New York, NY 10065, USA.

## Abstract

The aggregation of alpha-synuclein (aSyn) into intraneuronal inclusions of heterogeneous morphology, known as Lewy bodies (LBs), is a defining hallmark of Parkinson’s disease (PD); yet, our understanding of the mechanisms underpinning their formation and heterogeneity remains incomplete. Here, we present a human isogenic induced pluripotent stem cell–derived dopaminergic neuron (iDA) model that faithfully recapitulates the diverse biochemical, morphological, and ultrastructural features of LB neuropathology. The iDA model accurately reproduces the temporal relationships between neuritic and cell-body aSyn pathology and recapitulates the proteome, posttranslational modifications, and morphological diversity of aSyn aggregates found in human PD tissue. Moreover, our work provides critical insight into how different pathways to aSyn fibrillization and the complex interaction between aSyn fibrils and membranous organelles shape the morphological diversity of LB-like inclusions. This model represents a versatile platform to investigate the mechanisms of pathology formation, maturation, and neuronal dysfunction and to develop diagnostics and therapeutics that account for the diversity of aSyn pathology in PD and related synucleinopathies.

## Teaser

Human dopaminergic neurons reveal mechanisms shaping alpha-synuclein aggregation and Lewy body diversity in Parkinson’s disease.

## INTRODUCTION

Parkinson’s disease (PD) is a progressive neurodegenerative disorder for which there are no effective therapies or early diagnostic tools (*1*). The primary diagnostic neuropathological hallmarks of PD are the progressive degeneration of dopaminergic (DA) neurons in the substantia nigra (SN) pars compacta and accumulation of misfolded and aggregated forms of the presynaptic protein of alpha-synuclein (aSyn) in the form of the neuritic Lewy neurites (LNs) and cytoplasmic aggregates and inclusions, Lewy bodies (LBs) (*1*). The accumulation of misfolded aSyn aggregates is also observed in the brains of healthy aged individuals as well as in several other neurodegenerative disorders, collectively known as synucleinopathies. However, whether aSyn misfolding, aggregation, and inclusion formation are causes or consequences of the disease process remains a topic of active debate and investigation. This uncertainty is primarily due to the imperfect correlation between the presence and load of pathological aSyn aggregates, such as LBs and LNs, and the extent of neurodegeneration or the severity of PD symptoms.

Genetic mutations and multiplications of the SNCA gene, which encodes aSyn, or polymorphism in the promoter regions that regulate aSyn expression, have been linked to familial forms of PD or increased risk of PD (*2*). Furthermore, the detection of aSyn aggregates in the cerebrospinal fluid (CSF) of patients with PD, through the use of aSyn seed amplification assays, enables the diagnosis of PD with greater than 95% accuracy (*3*). This CSF seeding activity seems to correlate well with the presence or absence of aSyn fibrillar pathology in the brain, suggesting that aSyn fibrillar aggregates in the CSF correlate with brain LB-related pathology (*4*). Last, the presence of aSyn pathology in the brain correlates with cognitive decline in patients with PD and other synucleinopathies, e.g., Alzheimer’s disease (*2*, *5*, *6*). Despite converging evidence supporting a causative role for aSyn pathology in PD, several observations challenge its central role in the pathogenesis of all PD subtypes. These include the presence of aSyn pathology in the brains of aging healthy individuals (*7*, *8*), the lack of consistent correlations between aSyn pathology and neurodegeneration (*9*), and the absence of aSyn pathology in the brains of some patients with leucine-rich repeat kinase 2 (LRRK2) mutations who exhibit PD symptoms similar to those of sporadic PD (*10*). Two recent papers, using different proximity ligation assays and aSyn antibodies, demonstrated that widespread accumulation of oligomeric forms of aSyn occurs in the LB-negative LRRK2 mutation cases (*11*, *12*).

Several factors may contribute to the discrepancy in understanding the relationship between aSyn aggregation, neurodegeneration, and PD (*2*). These include the following: (i) the use of terminology that assumes uniformity among aSyn aggregates, i.e., the use of the terms aggregates or pathology to describe all forms of aSyn aggregates, despite extensive evidence of major morphological and biochemical heterogeneity in aSyn aggregates across the brains of patients with PD and other synucleinopathies; and (ii) the lack of cellular and animal models that enable investigating the evolution of LB or accurately replicate the complexity and heterogeneity of aSyn aggregation and pathology formation observed in the human brain (*13*).

A closer examination of Lewy pathologies in human brains reveals their morphological diversity and notable differences in the distribution of various molecular markers of LB (e.g., aSyn, ubiquitin, and neurofilaments), with the most notable differences observed between brainstem and cortical LB (*14*–*19*). Brainstem LBs, typically found in the SN and other regions of the brainstem, are associated with the motor symptoms of PD. In contrast, cortical LBs found in the hippocampus and neocortex are more frequently linked to cognitive impairments, such as those seen in dementia with LBs (*20*). Whether the association with different functional deficits could be attributed to differences in the biochemical, functional, and ultrastructural properties of aSyn aggregates remains unknown, in large part because of the failure to model aSyn pathological diversity in human neurons. Therefore, developing human neuronal models that recapitulate the formation of LBs and LNs over time and enable the dissection of their biochemical and ultrastructural properties is essential for investigating the mechanisms of aSyn pathology formation and dissecting the cell type–dependent relationship between this process and neurodegeneration in PD and other synucleinopathies.

Toward this goal, we developed a seeding-based isogenic induced pluripotent stem cell (iPSC)–derived dopaminergic neuron (iDA) model of aSyn LB formation that enables the investigation of aSyn fibrillization, pathology progression, and maturation under physiologically relevant conditions, specifically, in the absence of aSyn overexpression. iDA neurons treated with preformed fibrils (PFFs) of aSyn produce LB-like inclusions that closely replicate the diverse biochemical, ultrastructural, and morphological features of human LB pathology observed in idiopathic PD. We demonstrate that the differential distribution of aSyn fibrils and organelles, along with the specific types of membranous organelles that coaccumulate with aSyn inclusions, underlies the morphological diversity of LB-like inclusions in this model. In-depth characterization of these diverse inclusions suggests distinct mechanisms for the formation of aSyn pathology, including both organelle-dependent and organelle-independent pathways. Our work not only enhances the framework for studying LB maturation but also contributes to the standardization of seeding models, providing a more faithful and reproducible representation of the complex pathology observed in PD. This integrated approach offers a refined working model and a valuable platform for testing therapeutics and hypotheses regarding the intricate interplay between aSyn aggregation, organelle dysfunction, and pathology formation in PD and related synucleinopathies.

## RESULTS

### Internalization, processing, and clearance of aSyn PFF seeds over time in iDA

**Fig. 1.**
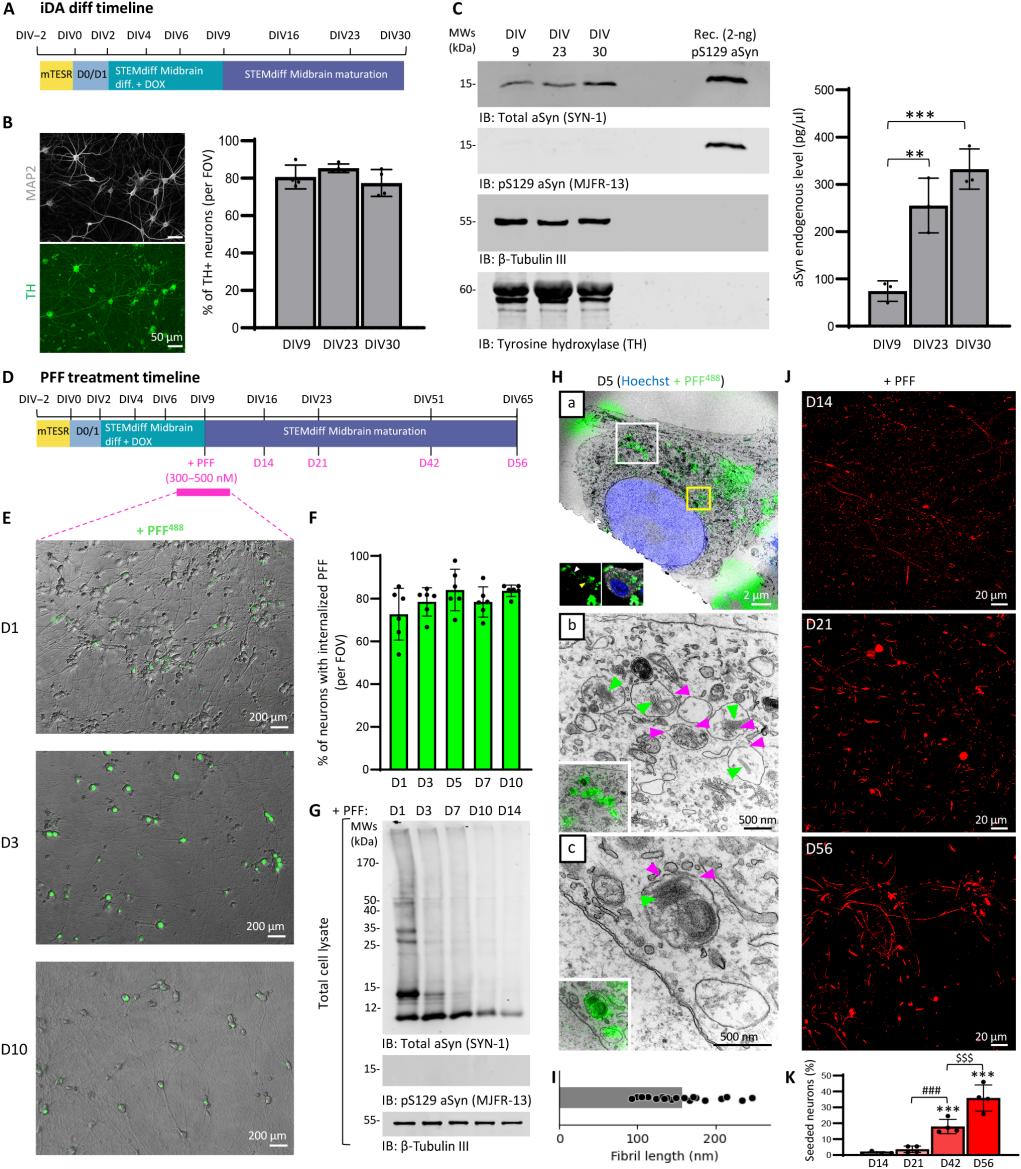
Internalization, processing, and clearance of aSyn PFF and subsequent pathology formation in iPSC-derived dopaminergic neurons. (**A**) Schematic of the iDA differentiation timeline using NGN2 programming. (**B**) ICC at DIV9 confirming dopaminergic differentiation (∼80 to 90% TH-positive neurons), with quantification at DIV9, DIV23, and DIV30. Cells were stained for MAP2 and counterstained with 4′,6-diamidino-2-phenylindole (DAPI). Scale bar, 50 μm. FOV, field of view. (**C**) Western blot (WB) analysis of total aSyn (SYN-1) and pS129 aSyn (MJF-R13) at DIV9, DIV23, and DIV30. β-Tubulin III and TH served as loading controls. aSyn levels were quantified relative to a 2-ng pS129 aSyn recombinant standard. (**D**) Seeding paradigm: 500 nM PFF added at DIV9; controls received phosphate-buffered saline (PBS). (**E**) Representative images of PFF^488^ internalization at D1, D3, and D10; individual channels are provided in fig. S3A. Scale bar, 200 μm. (**F**) Quantification of neurons containing internalized PFF from D1 to D10. (**G**) WB showing PFF processing: Internalized PFF (SYN-1) are cleaved to a ∼12-kDa fragment within 24 hours. β-Tubulin III served as a loading control. Densitometry quantifications are provided in fig. S3B. (**H**) CLEM at D5 showing PFFs within lysosomal-like vesicles (green arrows, intact; pink arrows, ruptured). Scale bars: 50 μm (a), 500 nm (b), and 200 nm (c). (**I**) Mean vesicular PFF size (∼156 nm). (**J** and **K**) Time-dependent development of pS129 pathology (81A antibody): neuritic aggregates at D14, with somatic inclusions emerging at D21 and increasing by D56. Only the pS129 channel from multichannel acquisitions is shown; corresponding MAP2, TH, and DAPI channels from the same fields of view are provided in fig. S5A. [(B), (C), (F), (I), and (K)] Data represent means ± SD from ≥3 independent experiments. Statistical analysis: analysis of variance (ANOVA) with Tukey post hoc test. (C) **P < 0.001, ***P < 0.0001 (DIV9 versus other time points). (K) ***P < 0.0001 (D14 versus other time points); ###*P* < 0.0001 (D21 versus D42); $$$*P* < 0.0001 (D42 versus D56).

The aSyn seeding model has been extensively studied and characterized in mammalian cell lines and primary neuronal cultures from rodents. In recent years, advancements in human stem cell technology have enabled the differentiation of iPSCs into different types of neuronal cells, including iDA (*21*–*44*). However, most available protocols for DA differentiation, until recently, required multistep processes taking several months (40 to 90 days) (*45*–*48*). In recent years, a protocol combining Neurogenin-2 (NGN2) programming and DA patterning has facilitated the rapid and efficient generation of human iPSC–derived midbrain neurons (iDA) (*49*, *50*) (Fig. 1A). In this study, we used this advanced cellular approach in combination with the seeding model to develop a robust model of PD in human-derived DA neurons.

First, we validated the effectiveness and reproducibility of the rapid protocol developed by Sheta *et al.* (*49*, *50*) (Fig. 1, A to C). Immunocytochemistry (ICC) combined with confocal imaging confirmed the differentiation of isogenic iPSCs into iDA (Fig. 1B and fig. S1). Among the neuronal population microtubule-associated protein 2 (MAP2)-positive, ∼80% of the neurons expressed tyrosine hydroxylase (TH), the rate-limiting enzyme in dopamine synthesis and a marker of DA neurons, after 9 days in vitro (DIV) (Fig. 1B). This differentiation efficiency was maintained over time, with a consistent ∼80% of the neurons expressing TH at DIV30. Western blot (WB) analysis of total cell lysates from these iDA cultures confirmed TH protein expression. In addition, we measured the concentration of endogenous aSyn in these cultures over time. Using recombinant monomeric aSyn as a standard, we estimated that endogenous aSyn was expressed at a concentration of 84 pg/μl at DIV9, which increased 3.5-fold by DIV30 to reach ∼300 pg/μl (Fig. 1C). At this protein expression level, we could not detect any signal for S129-phosphorylated aSyn (pS129) in iDA neurons (Fig. 1C).

Next, the iDA cultures were treated at DIV9 with aSyn PFFs (Fig. 1D and fig. S2) to induce seeding and formation of aSyn pathology as previously described (*51*–*54*). To assess the internalization of seeds by iDA neurons over time, we used wild-type (WT) human PFFs fluorescently labeled with Atto 488 (PFF^488^) (fig. S2C). A concentration of 300 nM was chosen to allow reliable quantification of uptake without saturating the fluorescent signal. One day after the addition of PFF^488^ to the extracellular media, we observed that ∼70 to 75% of the neurons had internalized the PFF seeds (Fig. 1, E and F, and fig. S3A). This uptake and accumulation, observed mainly as puncta in the cytosol, increased to ∼80% of the neurons with internalized PFF by day 3 (D3) posttreatment. The presence of the PFF remained detectable at the same level 10 days (D10) posttreatment (Fig. 1, E and F, and fig. S3A). This aligns with previous studies showing that PFFs are rapidly and efficiently internalized across various cellular models, including mammalian cell lines (*34*, *52*, *55*–*57*), primary neurons (*22*, *34*, *52*, *53*, *56*, *57*), and iPSC neurons (*22*, *25*, *28*, *32*, *34*, *35*, *40*, *52*, *56*, *58*–*61*), as well as in vivo following brain injection (*52*, *62*).

Biochemical analysis of total cell lysates from PFF-treated iDA cultures (Fig. 1G and fig. S3B) showed that within 24 hours of treatment, the internalized PFFs were C-terminally cleaved, as indicated by the appearance of a 12-kDa band. By 7 days (D7) posttreatment, the full-length aSyn (∼15-kDa band) was barely detectable. Although the truncated species were gradually cleared over time, a residual 12-kDa band remained detectable 14 days (D14) after PFF internalization. In addition, the detection of high–molecular weight aggregates decreased progressively over time. As expected, WB analysis also confirmed that the PFFs are not phosphorylated at serine S129. Similar observations have been reported by our group and others in primary neurons, iDAs, and mammalian cell lines treated with PFFs, as well as in mice injected with PFFs (*34*, *52*). Live imaging also showed that WT human PFFs fluorescently labeled with Atto 647 (PFF^647^; fig. S2D) are rapidly internalized by iDA cells through the endolysosomal pathway within minutes after adding PFFs to the extracellular medium (fig. S3, C to E), as previously described (*34*, *52*, *56*–*58*). Consistent with these results, correlative light electron microscopy (CLEM) combined with confocal microscopy confirmed the accumulation of the PFF^488^ in lysosomal-like vesicles (Fig. 1, H and I, and fig. S4). aSyn PFFs inside these vesicles measured ∼156 nm in length (Fig. 1I), consistent with the size distribution observed by electron microscopy (EM) during the characterization of the sonicated PFF preparation, which ranged from ∼50 to 250 nm (fig. S2A). We also observed that the membranes of the vesicles containing the PFFs appeared to be ruptured (pink arrows) or exhibited a less defined, diffuse membrane structure (Fig. 1H, c, and fig. S4). This is in line with a recent study showing that internalized PFFs trigger the perforation of the endolysosomal vesicles (*44*, *58*). Taken together, our results demonstrate that PFF are internalized and processed similarly in iDA cultures, as in other neuronal seeding models, such as PFF-treated primary WT or knockout neurons.

### aSyn PFF induce pS129 pathology in iDA

Next, we monitored the formation, maturation, and subcellular localization of pS129 pathology in iDA cells over time. We conducted ICC at D7, D14, D21, and D56 on iDA cells treated with 500 nM of unlabeled human aSyn PFF. No pS129 pathology was observed after D7 (fig. S5A). At D14, pS129 pathology was detected primarily in the neurites, with less than 1% of neurons showing cytosolic seeded aggregates (Fig. 1, J and K, and fig. S5A). In primary neuronal cultures, neuritic aSyn pathology has been reported in association with MAP2-positive processes (*53*), although precise assignment to dendrites versus axons remains challenging in dense cultures. In iDA neurons, pS129-positive aggregates were only rarely detected in MAP2-positive processes (fig. S5A) and were more frequently observed in neurites lacking MAP2 signal (fig. S5B). These observations remain descriptive and require further validation using experimental approaches that allow more definitive subcellular compartment assignment.

By D21, both neuritic and somatic pS129 pathology were observed; however, somatic pathology was present in fewer than 3% of the iDA neurons (Fig. 1, J and K, and S5A). At D56, ∼30 to 45% of the iDA neuronal cell bodies exhibited seeded aggregates (Fig. 1, J and K, and fig. S5A). It is worth noting that while 70 nM (∼1 μg/ml) of human seeds were sufficient to induce robust pathology in mouse primary neurons (*53*, *54*), a minimum concentration of 500 nM (∼7 μg/ml) of human PFFs was required to achieve a substantial level of pathology over time in iDA (Fig. 1, J and K, and fig. S5A). This finding aligns with other studies that used similar concentrations of PFF in iPSC-derived seeding models, where the reported effective range of PFF concentrations spans from ≤1 μg/ml (*30*, *40*, *44*, *56*, *59*) to 2 to 5 μg/ml (*25*, *28*, *32*, *34*, *35*, *52*, *60*) or 10 to 15 μg/ml (*22*, *32*, *39*, *41*, *63*) and up to 60 μg/ml (*29*). Even in lines with aSyn gene multiplication, a minimum of 5 μg/ml and up to 10 μg/ml was used (*32*, *39*, *63*). It is worth noting that under these conditions, mouse PFFs were unable to induce any pathology over time in our hands (fig. S5C). A likely explanation for the greater pS129 pathology observed in mouse primary neurons compared with iDA cultures is the fact that the sequence of the mouse aSyn-PFF and the substrate is the same, together with markedly higher endogenous mouse aSyn levels, estimated to be 7 to 10 times higher at early differentiation stages (DIV7 to DIV9) and around fourfold higher in more mature neurons (DIV30 to DIV35) (fig. S6, A and B). These findings underscore the importance of endogenous aSyn concentration as a key determinant of the initiation and the extent of pathological burden in neurons.

We were also intrigued by the fact that, despite ∼80% of the iDA neurons internalizing PFF seeds, only 3% exhibited pathology at D21. This was substantially lower than the 40% observed in primary mouse neurons under similar conditions (*53*). We hypothesized that variations in endogenous aSyn levels among neurons at DIV9, when seeds were added, or other differences in cellular factors that influence the initiation of seeding might explain why pathology developed in only a minor population at early stages (Fig. 1J). To estimate the local concentration of aSyn at the single-cell level, we performed ICC on untreated iDA cultures at DIV9. Neurons were costained with β-tubulin III (for the total neuronal population) and TH (for DA neurons) antibodies. Total aSyn levels in each cell were assessed using SYN-1 (epitope: 91 to 99) antibody. High-content imaging analysis revealed that endogenous aSyn was readily detected in both the soma and neurites of iPSC-derived neurons, consistent with observations reported in primary neuronal cultures and other neuronal cell–based models that do not fully recapitulate the maturity of adult human brain tissue, where aSyn is predominantly localized to presynaptic terminals (*64*) (fig. S6C). Quantification of total aSyn levels showed comparable expression across non-DA (β-tubulin III/TH−) and DA (β-tubulin III/TH+) neurons, regardless of whether TH expression levels were high or moderate (fig. S6, C and D). Because synaptic aSyn was barely detectable under our experimental conditions, this subcellular pool was not included as a separate parameter in the quantification. Notably, among TH+ neurons, 7% exhibited aSyn expression levels two to four times higher than the population average (fig. S6E). It is likely that these neurons with elevated aSyn levels are where seeding predominantly occurs, which may explain why, at D21, pathology was observed in only ∼3 to 5% of the total neuronal population (Fig. 1J). Overall, our findings highlight that although more than 80% of iDA cells internalized PFFs (Fig. 1, E and F), seeding initiation is a slow stochastic process occurring in only a few neurons at early stages.

### Seeded aggregates in iDA neurons exhibit LB pathological markers and a broad morphological spectrum similar to human brain pathology

**Fig. 2.**
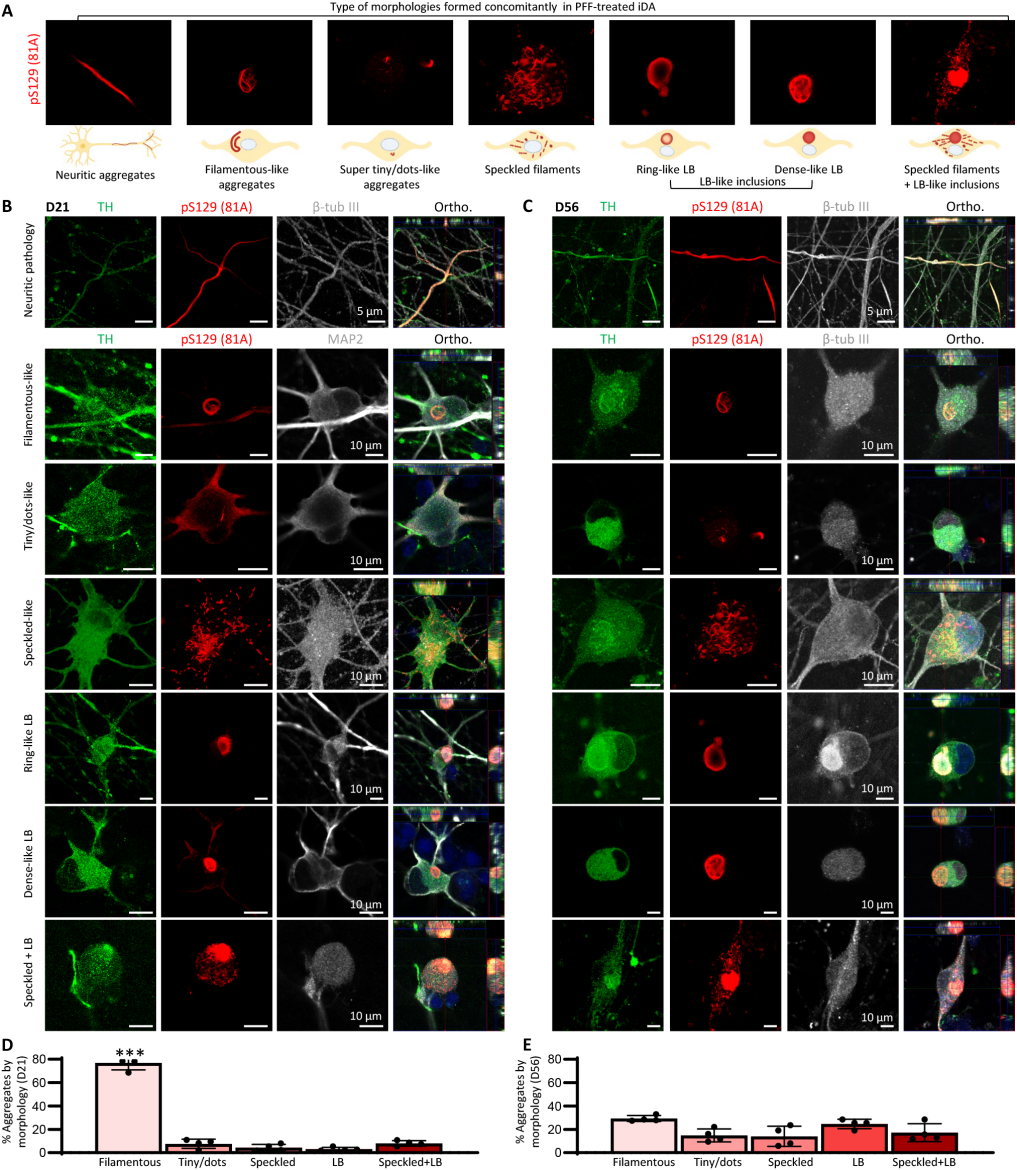
Seeded aggregates in iDA neurons exhibit a wide morphological spectrum similar to human brain pathology. (**A**) Schematic representation of the different types of morphological aggregates formed in PFF-treated iDA neurons: neuritic aggregates, filamentous-like aggregates, super tiny/dot-like aggregates, speckled-like aggregates, ring-like LBs, dense-like LBs, and LB-like inclusions. Representative images shown in (A) are presented in full, with an additional neuronal marker, in Fig. 2 or fig. S9. Schematic illustrations were generated using BioRender.com. Mahul-Mellier, A.L. (2026) https://BioRender.com/cayhdm7. (**B** and **C**) Representative images of iDA neurons at D21 (B) and D56 (C) showing the different aggregate morphologies positively stained with pS129 antibody (81A, red). Neurons were counterstained with TH (green), β-tubulin III, or MAP2 (gray) antibodies. Orthogonal projections (Ortho.). Scale bars, 5 μm [top row of (B) and (C)], 10 μm [bottom row of (B) and (C)]. (**D** and **E**) Quantification of the percentage of each type of seeded aggregate at D21 (D) and D56 (E), showing a predominance of filamentous-like aggregates at D21 and a significant increase in LB-like inclusions by D56. Data represent means ± SD from a minimum of four independent biological replicates (*N* = 4). Statistical analyses: ANOVA with Tukey post hoc tests. (D) ***P < 0.0001 (ANOVA followed by Tukey post hoc test, filamentous versus other morphologies). No statistically significant differences were observed in (E).

**Fig. 3.**
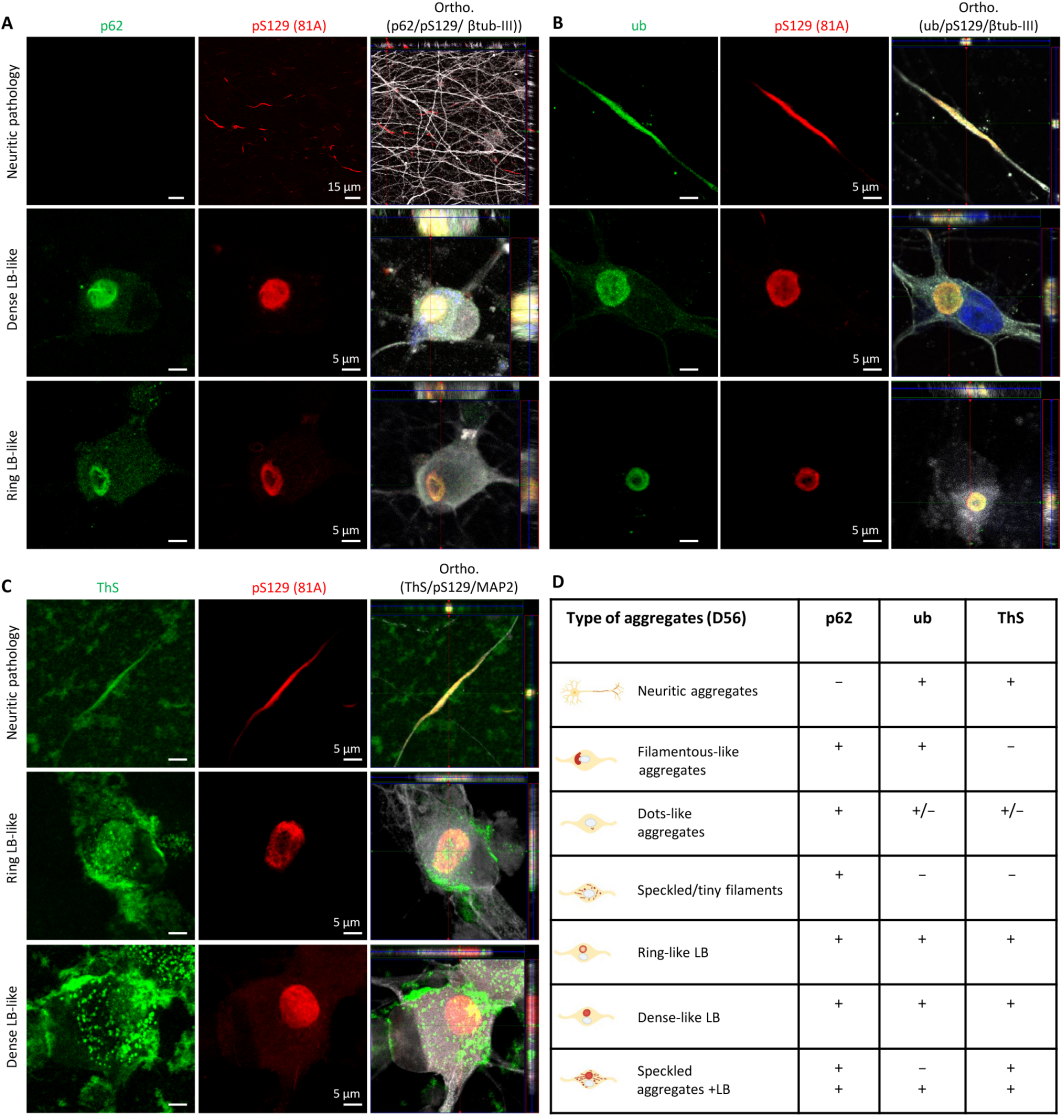
Seeded aggregates in iDA neurons exhibit the LB pathological markers similar to human brain pathology. (**A**) Representative images showing the differential sequestration of p62 and pS129 pathology in somatic and neuritic pathology at D56. p62 staining colocalized with the pS129-positive dense-like and ring-like LB-like inclusions but not with the neuritic pathology. (**B** and **C**) Ubiquitin [ub, (B)] and ThS (C) staining confirming β sheet amyloid-like species colocalized with both neuritic pS129 pathology and LB-like inclusions at D56. (A to C) Neurons were counterstained with β-tubulin III or MAP2 antibody. (**D**) Summary table showing the differential sequestration of p62, ubiquitin, and ThS in different types of seeded aggregates at D56 (see additional staining of the different types of seeded aggregates in fig. S8). Orthogonal projections (Ortho.). Scale bars, 5 μm [(A) to (C)]. Created in BioRender. MELLIER, A. (2026) https://BioRender.com/cayhdm7.

**Fig. 4.**
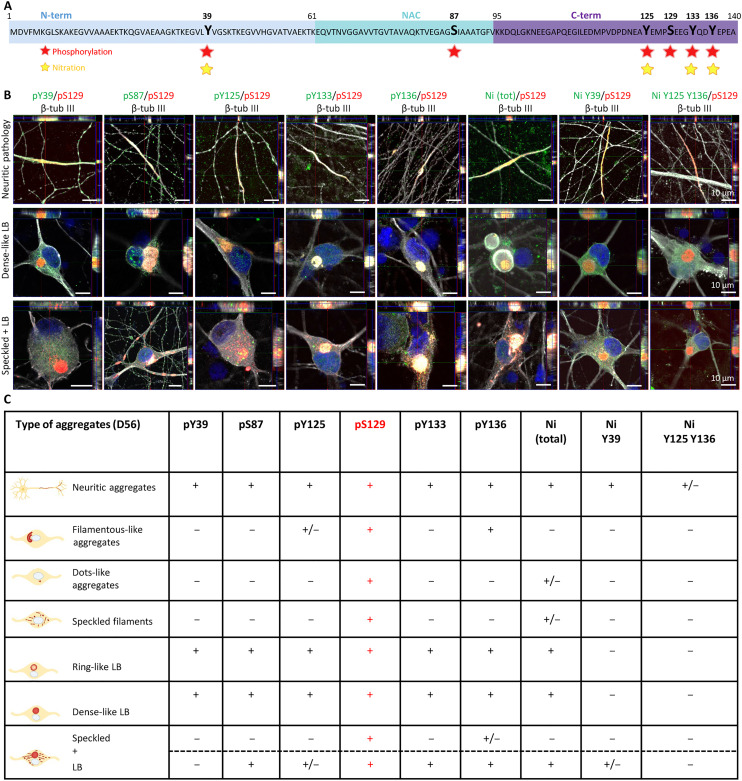
The iDA seeding model replicates the PTM signature associated with human pathology. (**A**) Schematic representation of PTMs analyzed in this study. These include phosphorylation at residue Y39, S87, Y125, S129, Y133, and Y136 and nitration at tyrosine residues (nY39, nY125, nY133, and nY136). All of these PTMs have been previously identified in aSyn pathology in postmortem brain tissues from patients with PD and MSA. (**B**) Representative confocal images at D56 illustrate the colocalization of pS129 with the PTM described in (A) across distinct aSyn aggregate morphologies within neuronal cell bodies. Images are shown as orthogonal projections of merged channels, including pS129, β-tubulin III, and the respective PTM-specific antibodies. Scale bar, 10 μm. Individual channels and additional aggregate morphologies, including dot-like, ring-like, filamentous, speckled, and neuritic inclusions, are shown in fig. S9. (**C**) Summary table displaying the distribution of specific PTM across the different aSyn aggregate types observed at D56, highlighting the diversity and selectivity of PTM profiles associated with each morphology. Schematic illustrations were generated using BioRender.com. Mahul-Mellier, A.L. (2026) https://BioRender.com/cayhdm7.

**Fig. 5.**
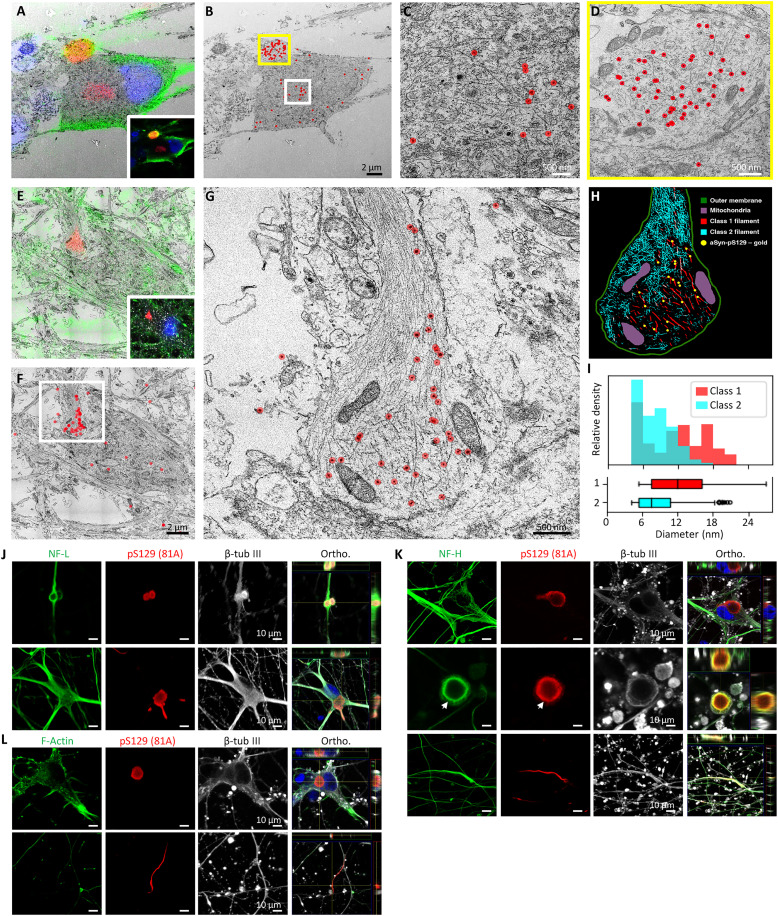
Seeded aggregates in iDAs are rich in fibrils that label positively for pS129 aSyn and that are distinct from cytoskeletal filaments. (**A**) ICC and EM overlay of a cell stained with fluoro-gold–conjugated antibodies binding to pS129 aSyn. The cell has an aggregate in its center and is attached to a small, dead cell (top) that is also immunopositive for pS129 aSyn. Inset: Immunofluorescence only. (**B**) Electron micrograph of the same cell, with gold-particle detections shown as red dots. (**C** and **D**) Zoomed insets of the aggregate inside the cell cytoplasm (C) and the dead cell (D) show the association of gold particles with filaments. (**E**) IF and EM overlay of another cell stained with fluoro-gold–conjugated antibodies binding to pS129 aSyn. (**F** and **G**) Gold-particle detections (red dots) agglomerate in the neurite of this cell, where two filament types can be distinguished: thick filaments associated with gold particles and thinner filaments that do not associate with gold particles. (**H**) Tomographic, neural network–based segmentations of gold-associated filaments (class 1) and thin filaments (class 2), together with manual segmentations of mitochondria and the neurite outer membrane. Gold-particle detections are shown in yellow. (**I**) Thickness measurements of the segmented filaments in class 1 (mean = 12.0 nm, SD = 5.2 nm) and class 2 (mean = 7.6 nm, SD = 3.0 nm). (**J** to **L**) Immunolabeling of the cytoskeletal proteins NF-L, NF-H, F-Actin, and β-tubulin III revealed no colocalization with pS129 staining in the soma of live cells. Scale bars, 2 μm [(B) and (F)], 500 nm [(C), (D), and (G)], and 10 μm [(K) and (L)].

**Fig. 6.**
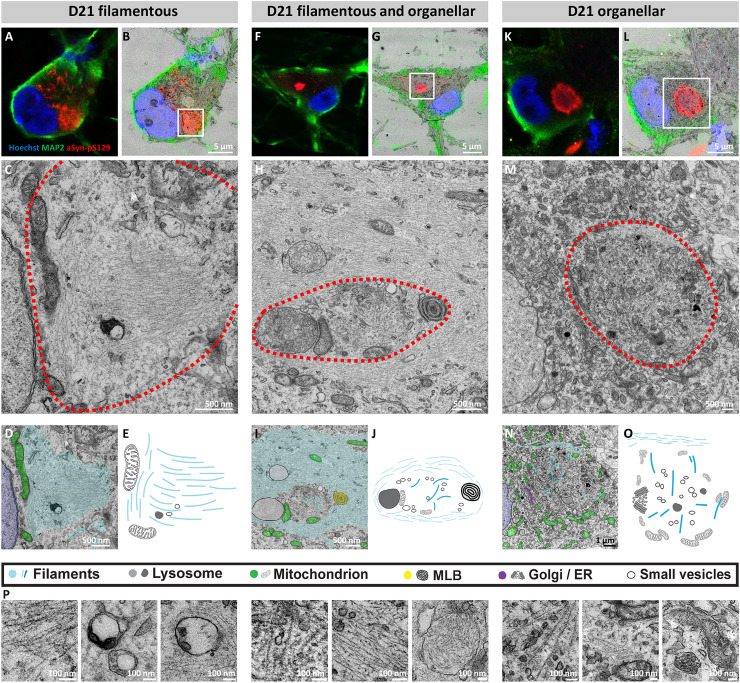
CLEM of pS129 aSyn aggregates at D21 in PFF-treated iDA. Neurons were fixed in 4% PFA, permeabilized by rapid freezing and thawing in the absence of detergent to keep the ultrastructure intact, and immunolabeled with pS129 (81A) antibody. After confocal immunofluorescence imaging, the same cells were subjected to heavy-metal staining, resin embedding, ultrathin sectioning, and imaged with a transmission electron microscope. Aggregates were classified into three groups on the basis of their ultrastructure. (**A** to **E**) A predominantly filamentous ultrastructure, where pS129 immunofluorescence correlates with a dense mesh of thin, parallel filaments. Small vesicles and electron-dense lysosomes are sparsely mixed with the filaments. (**F** to **J**) A filamentous mesh surrounds a dense aggregate of small vesicles, membrane-bound organelles and thick (13 ± 3.5 nm) fibrils, which are sparsely and randomly distributed. The membranous aggregate correlates with bright pS129 immunofluorescence [(F) and (G)], while the immunofluorescence in the filamentous mesh is less bright and more punctate. (**K** to **O**) pS129 immunofluorescence correlates with an ultrastructure dense in membranes, thick (13 ± 3.5 nm) fibrils and membrane-bound organelles, in particular mitochondria, lysosomes, and small vesicles. (**P**) Representative images of components within aggregates at D21, notably, filaments, small vesicles, lysosomes and mitochondria. Scale bars = 100 nm. Scale bars, 5 μm [(B) and (G) to (L)] or 500 nm [(C) and (H) to (M)] or 100 nm (P).

**Fig. 7.**
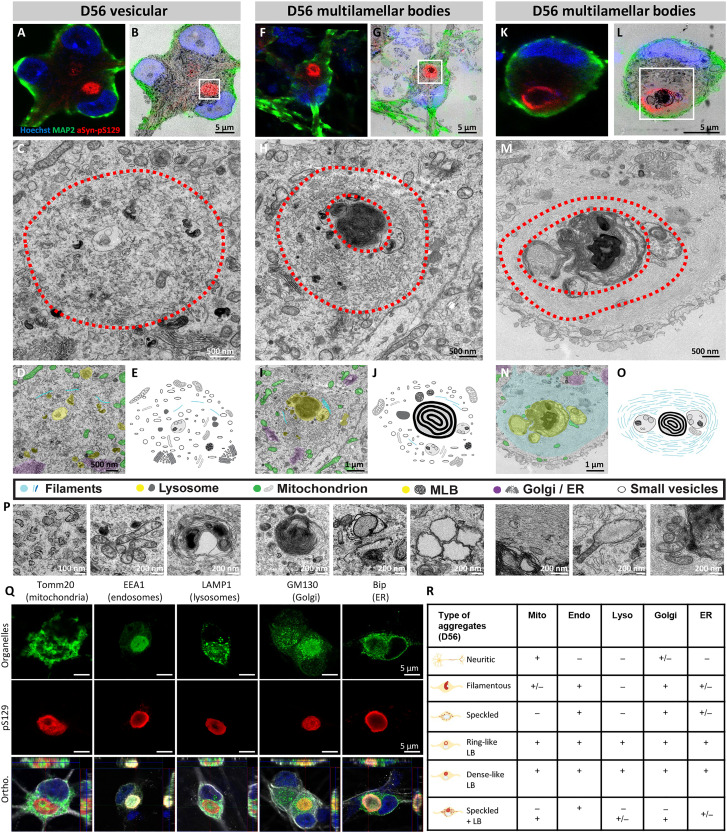
CLEM of pS129 aSyn aggregates at D56 in PFF-treated iDA. Aggregates were classified into three groups on the basis of their ultrastructure. (**A** to **E**) A uniform pS129 immunofluorescence, which correlates with an ultrastructure rich in small vesicles, lysosomes, clustered mitochondria, and long, thick filaments (13 ± 3.5 nm). (**F** to **J**) A ring-shaped pS129 immunofluorescence, of which the center correlates with the presence of membrane-rich multilamellar bodies (MLBs). Surrounding the MLBs are small vesicles and sparse, thick filaments (13 ± 3.5 nm). (**K** to **O**) A similar ring-shaped pattern of pS129 immunoreactivity with MLBs in its center but here surrounded by a dense mesh of parallel, thin (8 ± 2 nm) filaments. (**P**) Representative images of components within aggregates at D56, notably, small vesicles, MLB, filaments and mitochondria. Scale bars, 5 μm [(B) and (G) to (L)] or 500 nm [(C) and (H) to (M)] or 100 nm (P). (**Q**) Representative images show pS129 pathology (red, 81A antibody) alongside markers for key organelles: GM130 for the Golgi apparatus, BiP for the ER, EEA1 for endosomes, LAMP1 for lysosomes, and TOMM20 for mitochondria (all in green). Orthogonal (Ortho.) projections confirm that all these organelles except the ER are sequestered into pS129 LB-like inclusions at D56. Neurons were stained with the β-tubulin III antibody, while nuclei were counterstained with DAPI. Scale bars, 5 μm. (**R**) Summary table depicting the sequestration of cellular organelles across the distinct aSyn aggregate morphologies observed at D56.

**Fig. 8.**
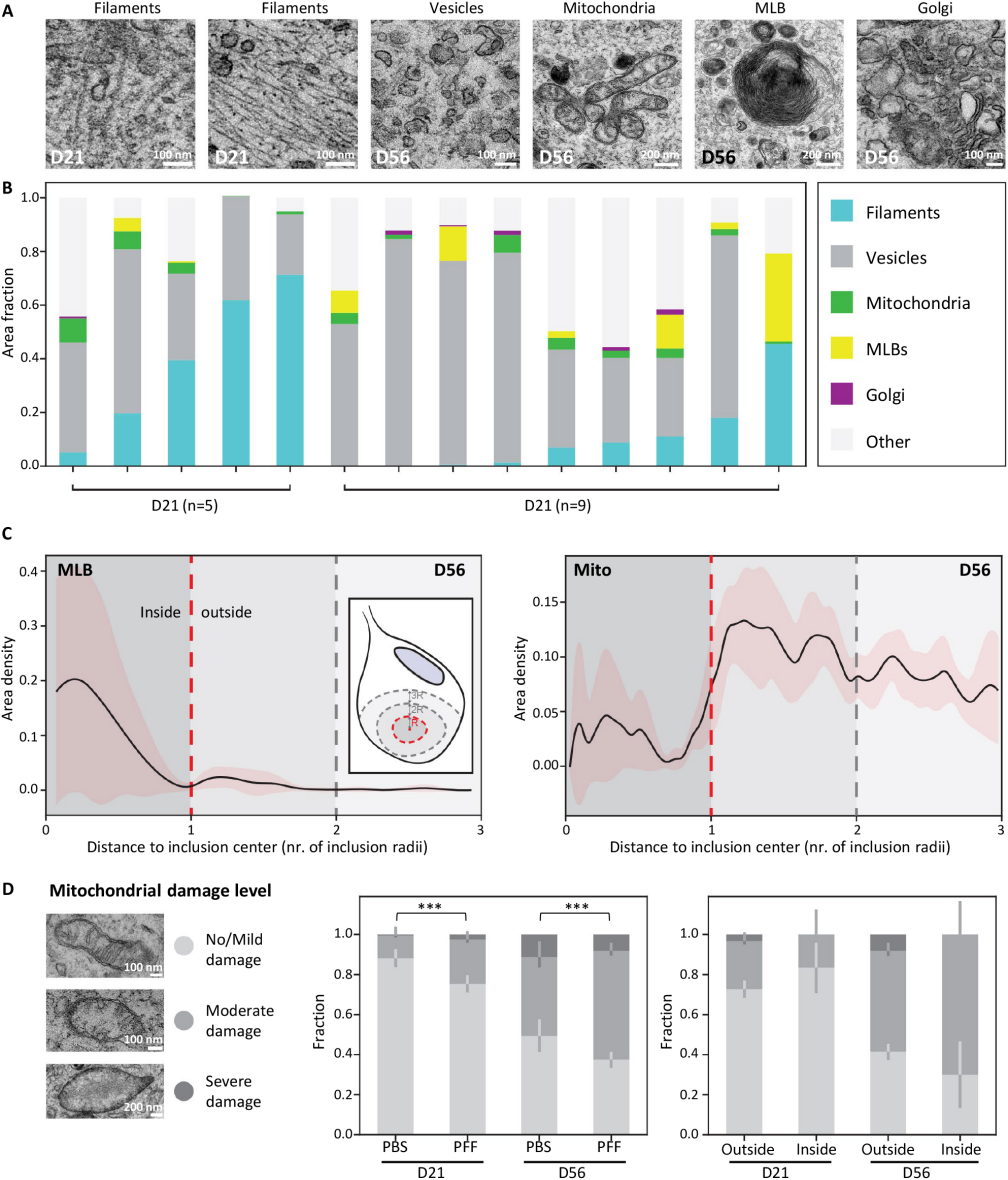
Inclusion composition and mitochondrial damage of seeded aggregates in PFF-treated iDAs. (**A**) Representative ultrastructural components observed within aggregates at D21 and D56, including filamentous structures (thick filaments of ∼13 nm, left; thin filaments of ∼8 nm, right), small vesicles, mitochondria, MLB, and Golgi apparatus. The filament panels are derived from images shown in Fig. 6, while vesicle-, mitochondria-, and MLB-containing panels are derived from images shown in Fig. 7; these are reused here as representative examples of the ultrastructural classes quantified in (B). Scale bars, 100 nm. (**B**) Area proportion of the abovementioned ultrastructural components in electron micrographs through the central slice of five D21 aggregates and nine D56 aggregates. (**C**) Area of MLBs (left) and mitochondria (right) relative to the total pS129-positive area at defined distances from the aggregate center (0 = center, 1 = periphery, 2 to 3 = one to two radii away). The black line indicates the mean across nine aggregates (nine cells); shaded area shows the 95% confidence interval (bootstrapping). (**D**) Three representative images show the classification of mitochondria into three classes according to their level of damage. Scale bars, 100 nm. The left plot shows the mean fraction of each class in PBS-control cells or PFF-treated cells with aggregates at D21 and D56. The right plot shows the fraction of each class inside and outside the inclusions of PFF-treated cells with aggregates, at D21 and D56. Error bars indicate one-half of the 95% confidence intervals for each class, calculated using bootstrapping. Data represent means ± SD from a minimum of three independent biological replicates (*N* ≥ 3). Statistical analyses: A chi-square test indicates significance between PBS- and PFF-treated cells with ***P < 0.0001 for D21 and D56.

**Fig. 9.**
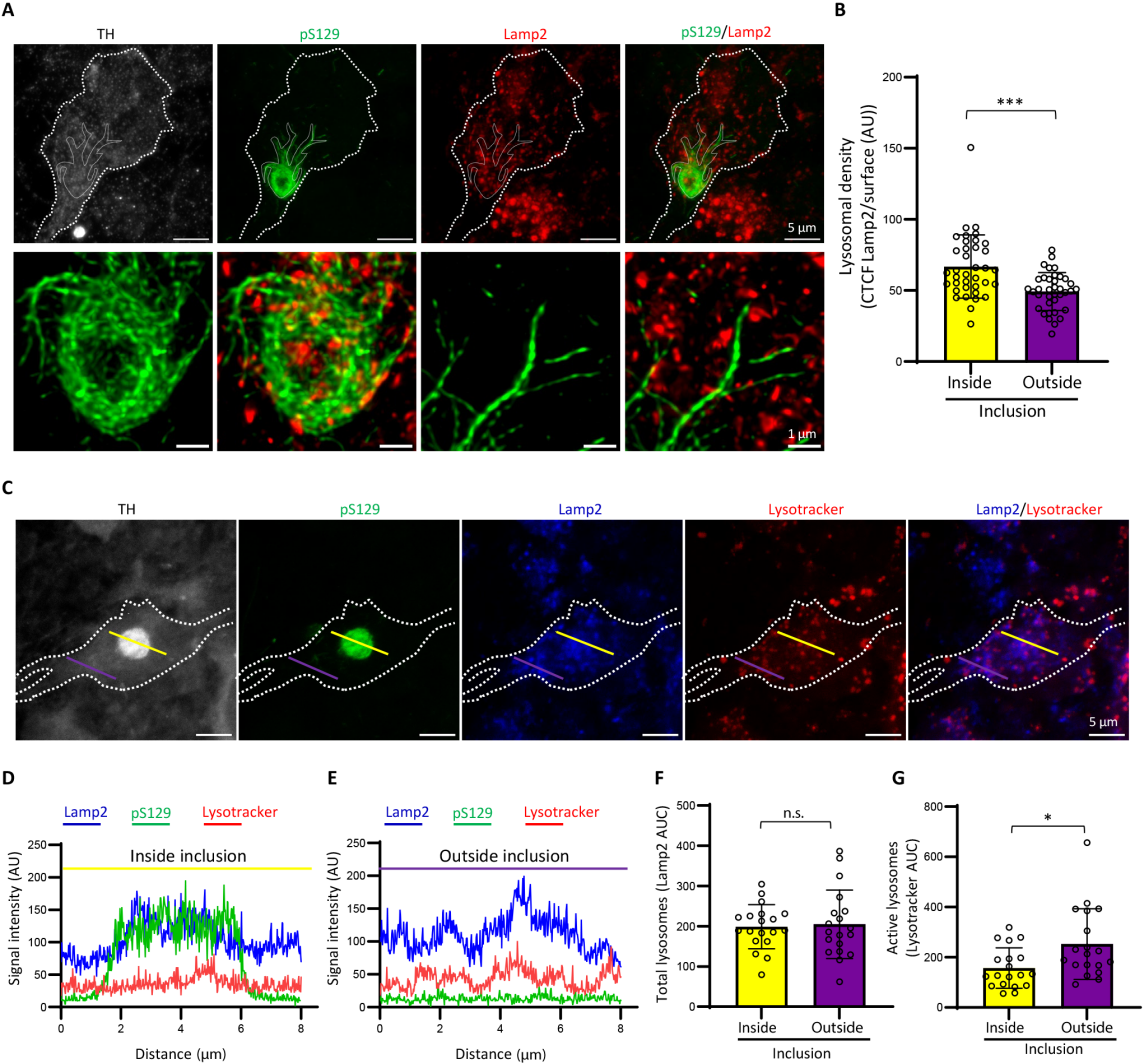
aSyn pathology disrupts lysosomal function in iDA. (**A**) Confocal microscopy images (top) of iDA neurons (stained for TH, gray), 42 days postaddition of aSyn PFF, showing a radiating aSyn inclusion (pS129, green) interacting with Lysosome-associated membrane protein 2 (LAMP2, red). Scale bar, 5 μm. STED microscopy images (bottom) highlight accumulation of lysosomes inside and around the inclusion core, as well as along radiating fibers. Scale bar, 1 μm. (**B**) Fluorescence intensity relative to lysosomes (red) was measured inside and outside the inclusion (green) in the same TH+ neuron to evaluate spatial lysosome distribution. (**C**) Confocal microscopy images of iDA neurons (TH, gray) showing a dense aSyn inclusion (pS129, green), total lysosomes (LAMP2, blue), and active lysosomes (lysotracker, red). Scale bar, 5 μm. (**D**) Fluorescence intensity profiles show lysosomes (blue) are less active (red) inside the inclusion (yellow line), with few lysotracker spikes compared with LAMP2 (dark arrow). (**E**) In contrast, lysosomes outside the inclusion (purple line) show synchronized lysotracker (dark arrows) signals with LAMP2, indicating functionality. (**F**) Quantification of area under the curve (AUC) for LAMP2 shows no significant difference between inside and outside the inclusion, indicating stable total LAMP2 levels. n.s., not significant. (**G**) AUC quantification for lysotracker. Shows a significant reduction inside the aSyn inclusion, indicating reduced lysosomal function. [(B), (F), and (G)] Data represent means ± SD (*N* = 3 independent biological replicates). Statistical analyses: unpaired *t* test. (B) Lysosomes are significantly increased inside inclusions compared with the cytosol (***P < 0.0001, unpaired *t* test). (F) No significant difference. (G) *P < 0.05.

Next, we conducted an in-depth characterization of pS129 pathology in both neurites and cell bodies, focusing on how our seeded aggregates reproduce PD-specific features observed in patient brain tissues. We compared the LB-like inclusions formed in PFF-treated iDA neurons to those found in human PD brain tissues, first assessing whether our seeded aggregates could replicate the full spectrum of LB morphologies observed in patients (Fig. 2). In addition, we examined key pathological markers (p62 and ubiquitin) (Fig. 3), posttranslational modification (PTM) signatures (Fig. 4), and the ultrastructural organization and morphology characteristic of bona fide LB inclusions (Figs. 5 to 7), as well as how organelle sequestration and dysfunction contribute to LB formation and maturation (Figs. 8 and 9). First, we observed a broad morphological spectrum of aggregates formed over time (Fig. 2). In addition to neuritic pathology, we classified the somatic-seeded aggregates into six categories: filamentous-like aggregates, tiny dot-like aggregates, speckled-like aggregates, and three types of LB, including dense-like LB, ring-like LB, and LB (dense- or ring-like) surrounded by speckled aggregates (Fig. 2A). This morphological diversity was evident at both D21 (Fig. 2B) and D56 (Fig. 2C).

At D21, filamentous-seeded aggregates were the predominant species (∼60%), with barely any LB-like structures detected (<5%) (Fig. 2, B and D). By D56, ∼45% of the seeded aggregates were classified as LB-like inclusions (Fig. 2, C and E). These findings suggest that aSyn fibrillization is required for LB formation. Immunostaining showed that TH was absent from early filamentous or speckled aggregates but frequently colocalized with more mature LB-like inclusions (Fig. 2 and fig. S7). This relocalization was observed in various inclusion morphologies (fig. S7). In some neurons, TH was diffusely distributed within the inclusion, while in others, it was sharply concentrated at the periphery or appeared fully sequestered. Where TH was strongly enriched within aggregates, the surrounding cytoplasm often appeared nearly devoid of TH staining, suggesting a progressive sequestration of this enzyme from the cytoplasm into the inclusions over time. Notably, this pattern mirrors observations in the putamen and SN of patients with PD and multiple system atrophy (MSA), where TH has been shown to accumulate within LB inclusions while its levels in the surrounding cytoplasm are reduced (*65*). This suggests that the formation and maturation of LB inclusions may disrupt the production of l-dopa and dopamine by reducing the availability of the rate-limiting enzymes necessary for dopamine synthesis (*66*, *67*). Thus, the presence of TH within pS129-positive aggregates in our model supports the idea that aSyn pathology can disrupt DA neuron function through altered TH localization and enzymatic activity, in direct agreement with observations from human brain tissue.

In our model, neuritic aSyn pathology consistently appears by D14, preceding the formation of somatic inclusions. We also observed that different types of seeded aggregates within neurons exhibit differential immunoreactivity of key LB pathological markers, including p62, ubiquitin, and β sheet dyes such as Thioflavin S (ThS), depending on their type and localization (Fig. 3 and fig. S8). p62 staining colocalized with all somatic pS129-positive aggregates, regardless of morphology, but not with neuritic pathology (Fig. 3A and fig. S8A), consistent with previous reports in mouse neuronal seeding models (*34*, *51*–*53*) and human brain tissues (*14*). Conversely, ubiquitin strongly labeled both pS129-positive neuritic aggregates and somatic inclusions, except for the speckled species (Fig. 3B and fig. S8B). Neuritic pS129 pathology and mature LB-like inclusions were also strongly positive for ThS, confirming the presence of β sheet amyloid-like species, while filamentous and speckled aggregates did not colocalize with ThS, although the dye was often found in close proximity (Fig. 3C and fig. S8C). Notably, these neuritic aggregates remained p62 negative (Fig. 3A), consistent with findings in human PD brain (*14*), where such inclusions are considered to reflect an earlier or distinct aggregation state.

This compartment-specific difference suggests that neuritic and somatic aggregates follow distinct molecular pathways or exhibit different kinetics of formation and maturation. Kuusisto *et al.* (*14*) first described these differences, and more recently, Lewis *et al.* (*15*) showed that neuritic aggregates often contain a mix of fibrillar and membranous material, which may provide a microenvironment for initial seeding that later propagates to the soma. Together, these findings indicate that seeded aggregates differ in composition and binding properties and that amyloid dyes such as ThS may not detect all forms of aSyn pathology, as recently noted by De Giorgi *et al.* (*68*).

### The iDA seeding model replicates the PTM signatures associated with the human pathology

Various PTMs, including phosphorylation at S129 and serine-87 (pS87), ubiquitination, and nitration [nitration at tyrosine-39 (nY39) or the C-terminal tyrosine residues (nY125/nY133/nY136)], have been identified in aSyn aggregates and LBs within the postmortem brains of patients with PD or MSA (*18*, *19*, *52*) (Fig. 4A). These modifications are believed to influence aSyn aggregation, aSyn fibril interactome, and inclusion formation and maturation, as well as toxicity and disease progression (*51*, *53*, *69*). Therefore, we sought to determine whether the iDA seeding model recapitulates human pathology at the level of PTMs.

Toward this goal, we used an expanded antibody toolset targeting different sequences within different domains of aSyn and most of the disease-associated PTMs, and it was recently validated in human brain tissues from patients with PD, MSA, and LBD (*18*, *19*, *70*). We analyzed the PTM signatures of aSyn neuritic and somatic pathology that developed over time in PFF-treated iDA neurons. Since only 3% of neurons showed somatic aggregates at D21, we chose to assess PTM signatures at D56, when ∼35% of neurons displayed seeded aggregates encompassing a wide spectrum of morphologies.

Neuritic pathology, which forms early in the aggregation process, consistently exhibited all major disease-associated PTMs, including pS129, pS87, pY39, pY125, pY133, and pY136. Strong colocalization of these PTMs with pS129-positive neuritic aggregates was observed (fig. S9B), reflecting an early, PTM-rich fibrillar state. This pattern extended to residue-specific nitration, with neurites staining positive for nY39, nY125, and nY136, as well as for total nitrated aSyn (fig. S9C). It is not clear whether these modifications co-occur on the same fibril and what percentage of the fibrils carry these modifications.

By contrast, PTMs incorporation in somatic aggregates was highly morphology-dependent. Among the various aggregate types, only LB-like inclusions, either dense or ring-like, showed strong and consistent colocalization with multiple PTMs, including pS87, pY125, pY133, pY136, and residue-specific nitration (Fig. 4 and fig. S9, D to G). In neurons containing both LB-like inclusions and surrounding speckled aggregates, the LB-like structures were PTMs positive while the adjacent speckled pathology remained negative, indicating spatially segregated PTM patterns within the same cell (Fig. 4 and fig. S9, H and I). In smaller somatic aggregates, such as dots, filamentous, or speckled forms, PTM signals were often restricted to surrounding granules (e.g., pS87 or pY125), rather than fully colocalizing with the aggregates themselves (fig. S9, J to M, white arrows). Dot-like and speckled aggregates consistently lacked pY136 staining.

Total nitrated aSyn was detected in most seeded aggregates except filamentous ones; however, only neuritic pathology and LB-like inclusions showed clear labeling with nY39 and nY125/nY136 antibodies. An exception was the LB-like inclusion surrounded by speckled pathology, which also stained positively for nY39. These findings are consistent with recent studies from our laboratory suggesting that nitration may reflect a late-stage modification to neutralize the pathogenic activity of aSyn fibrils and LBs (*69*).

Together, this represents the most comprehensive PTMs characterization of seeded aSyn pathology in human-derived neurons to date. Our findings demonstrate that PTMs pattern is tightly linked to both the morphology and subcellular localization of aSyn aggregates. Neuritic pathology rapidly accumulates disease-relevant PTMs, while somatic aggregates require higher-order organization, specifically LB-like architecture, to exhibit full PTM signatures. These results highlight the complex biochemical landscape of seeded aSyn pathology and support the notion that distinct PTM patterns may reflect maturation stages, structural classes, or divergent aggregation pathways. Whether these modifications precede or follow fibrillization remains to be determined.

### CLEM reveals a large diversity of pS129-positive inclusions at the ultrastructural level

The morphological and biochemical similarities between seeded aggregates in iDA neurons and bona fide LBs in the human PD brain, including pathological markers, a broad morphological spectrum, and PTM signatures, motivated us to proceed with characterizing the ultrastructure of aSyn pathology formed in iDAs using CLEM. We hypothesized that such major differences in their biochemical signatures should be reflected in the composition and ultrastructural properties of these different types of aggregates. Given that LB-like aggregates are enriched in membranous structures and organelles, it is crucial to preserve membrane integrity and avoid the damage caused by detergents like Triton. To achieve this goal, we developed a detergent-free ICC protocol based on rapid freeze–thaw permeabilization (see Materials and Methods). In this approach, iDA neurons were fixed in 4% paraformaldehyde (PFA) at D21 or D56 and permeabilized by rapid freezing and thawing without detergent, thus preserving the ultrastructure of neurons, inclusions, and organelles. Last, to validate the presence of aSyn fibrils, they were immunolabeled with a pS129 antibody (81A) (Figs. 5 to 8).

Neurons with pS129 pathology were identified with confocal immunofluorescence imaging, and the same cells were heavy-metal stained, resin-embedded, ultrathin sectioned, and imaged with a transmission electron microscopy (TEM) as previously described (*52*, *53*). In most inclusions, TEM imaging revealed the presence of fibrils, which colocalized with pS129 aSyn fluorescence immunopositivity (Figs. 5 to 8).

Neuritic aggregates were composed of aSyn filaments and thinner filaments, likely corresponding to neurofilaments, surrounding mitochondria, structures similar to those described by Lam *et al.* (*32*). Pre-embedding immunogold labeling confirmed that these structures were indeed aSyn fibrils (Fig. 5, A to I) rather than cytoskeletal proteins of similar diameter (Fig. 5, G to I). ICC also confirmed the absence of colocalization of the pS129 somatic or neuritic pathology with the filamentous cytoskeletal proteins, including the three main subunits of the neurofilaments, namely, NF-L, NF-M and NF-H, and β-tubulin III or actin (Fig. 5, J to L, and fig. S10). Despite this general lack of association, in this model, NF-H recruitment at the periphery of the LB-like inclusion was observed only in dead neurons (Fig. 5K, white arrow).

At D21 (Fig. 6 and fig. S11), the number and density/distribution of fibrils present in each inclusion varied greatly, with some inclusions predominantly composed of long fibrils. In contrast, others mainly consisted of small vesicles intermixed with organelles and a sparse number of randomly oriented fibrils. The ultrastructure of a speckled aggregate consisted of long filaments that were organized in a parallel fashion and devoid of membranous organelles (Fig. 6, A to E). In contrast, the dense inclusions were consistently associated with a mixed ultrastructure of small vesicles, lysosomes, mitochondria and fibrils (Fig. 6, F to O, and fig. S11). When the dense LB was surrounded by speckled pS129 staining, these speckles colocalized with patches of predominantly long, parallel-associated filaments curving through the cytosol and sometimes enclosing the dense aggregate (Fig. 6H). Another example of a large, dense LB also consisted of membranes intermixed with fibrils but was surrounded by a thin layer of filaments that presumably belonged to the intermediate filament family (Fig. 6M). The condensation of pS129 immunostaining at the periphery of this aggregate correlates with an ultrastructure enriched in mitochondria.

At D56, the CLEM analysis confirmed the ultrastructure of three more dense LBs to be a mix of membranous organelles and fibrils, although fewer fibrils and more vesicular structures were present compared with D21 (Figs. 7, A to O, and 8, A and B, and fig. S12). In total, we identified five ring-like LB-like inclusions and characterized their ultrastructure (fig. S12). The ultrastructure of the pS129-immunopositive halo was found to be heterogeneous: In three examples, it consisted of parallel-associated filaments (Fig. 7M), whereas in the remaining two examples, it had an ultrastructure that was rich in small vesicles and sparse fibrils (Fig. 7, C and H). Using specific antibodies for each organelle, we confirmed that mitochondria, lysosomes, endosomes, the Golgi apparatus, and the endoplasmic reticulum (ER) are differentially sequestered on the basis of the type and subcellular localization (neurites versus soma) of aSyn-seeded aggregates, indicating distinct organelle involvement at various stages of aggregate formation and maturation within iDA neurons (Fig. 7, P and Q, and fig. S13). Despite their ultrastructural heterogeneity (Fig. 8B), a common feature of almost all inclusions observed by CLEM was an enriched density of mitochondria at the periphery of the pS129-immunopositive region (Fig. 8C), which is consistent with human brain LB pathology.

Mitochondria were consistently associated with nearly all types of aggregates, including neuritic aggregates, filamentous-like aggregates, ring-like LB, dense-like LB, but not with the speckled LB (Fig. 7, P and Q). Lysosomes, on the other hand, were specifically recruited only to LB-like inclusions, such as ring-like LB and dense-like LB. This restricted involvement suggests that lysosomes play a key role in the processing and maturation of advanced aggregates. Endosomes, however, were found in all aggregate types except neuritic aggregates, suggesting a more specialized role in the progression and processing of more developed aggregates. The Golgi apparatus was consistently recruited to all aggregate types, including neuritic aggregates, filamentous-like aggregates, and all LB-like inclusions. This indicates that Golgi-related processes are implicated across all stages of aggregation, from early to mature inclusions. Although EM clearly revealed the presence of fragmented and damaged ER within LB-like inclusions, the ER was difficult to detect by ICC (fig. S13C). The Binding immunoglobulin Protein (BiP) showed no colocalization, whereas calreticulin labeling revealed only partial ER signal, suggesting that the limited ICC detection may result from epitope masking or structural disruption of the ER within inclusions. These findings collectively highlight the organelle-specific roles in aSyn aggregate formation and maturation, with mitochondria, lysosomes, the Golgi apparatus, and endosomes contributing distinctively to different stages of the aggregation pathway.

Notably, in all five examples (Fig. 7 and fig. S13), the center of the ring, where the pS129 immunopositivity is weak, consistently correlated with the presence of electron-dense multilamellar bodies (MLBs). These MLBs were only observed within LB-like inclusions and were never detected in the surrounding cytoplasm (Fig. 8C). These MLBs exhibited strong immunoreactivity for Lysosomal-Associated Membrane Protein 1 (LAMP1), a lysosomal marker, confirming their lysosomal identity (*71*, *72*) (Fig. 7, H to M). These LAMP1-positive structures reflect a specific stage of lysosomal and autophagy failure (*71*), where impaired degradation of membranes, organelles, and protein aggregates creates a microenvironment prone to membrane stacking, lipid peroxidation, and progressive MLB formation. In addition, the center of ring-like inclusions showed enrichment of Translocase of Outer Mitochondrial Membrane 20 (TOMM20), a mitochondrial outer membrane marker, while intact mitochondria were notably absent on the EM micrographs (Fig. 7P). This spatial and molecular profile suggests that MLBs may form through lysosomal processing of mitochondrial components. MLBs are membrane-bound organelles composed of concentric lamellae, typically associated with lysosomes and known to accumulate when lysosomal degradation is impaired. Their presence within LB-like inclusions, combined with LAMP1 and TOMM20 positivity, points to a convergence of mitochondrial turnover and lysosomal activity under stress conditions. Consistent with this, a recent study demonstrated that PFF-injected mice develop MLBs in perinuclear regions of DA neurons in the SN (similar to Fig. 7, H to M), where they co-occur with autophagosomes, endolysosomes, and oxidized mitochondria, reflecting active organelle remodeling and stress responses (*62*).

In our study, MLBs were consistently observed within somatic LB-like inclusions. Postmortem EM studies found MLBs to be enriched in the brain of patients with Alzheimer’s disease, where they localize close to tau fibrils in somatic neurofibrillary tangles (*73*, *74*). Here, they are thought to arise resulting from autophagic stress and impaired proteostasis (*71*, *74*). In PD postmortem brain, dense and lamellated bodies were noted by Forno and Norville (*75*) not in the soma but in axonal swellings associated with LB pathology. Rosette-like lamellae were later also observed within membranous inclusions in PD (*17*). Together, these observations suggest that MLB within LB-like inclusions represents a pathological interface between mitochondrial degradation and lysosomal dysfunction, marking them as indicators of disrupted organelle homeostasis in aSyn-driven pathology formation and neurodegeneration (*17*, *71*, *73*–*75*).

Next, we used EM to evaluate mitochondrial health in the iDA seeding model. Mitochondria in PFF-treated cells with inclusions, as well as those in phosphate-buffered saline (PBS)–treated control cells, were classified in three groups on the basis of their level of damage (Fig. 8D): no/mild damage, moderate damage (light swelling and/or decreased number of cristae), and severe damage (almost complete loss of cristae). In both D21 and D56, the fraction of damaged (moderately and severely) mitochondria was significantly increased in cells with inclusions compared with PBS-treated control cells, indicating that inclusion formation negatively affects mitochondrial health. The majority of severely damaged mitochondria was found outside and in close proximity to the pS129-immunopositive inclusions, not within (Fig. 8D), possibly because severely damaged mitochondria within inclusions might give rise to MLBs in the process of inclusion formation.

Together, our results at D21 and D56 suggest that the evolving morphological diversity of aSyn aggregates is likely driven by multiple, interconnected or interdependent factors. These include differences in fibril abundance, the spatial distribution and ultrastructural organization of fibrils and membranous organelles within inclusions, as well as variations in their biochemical composition and PTMs. In addition, the selective recruitment and sequestration of specific organelles, such as mitochondria, lysosomes, endosomes, and the Golgi apparatus, further contribute to the heterogeneity of aggregate structure and likely reflect stage-dependent remodeling events and cellular stress responses. Together, these elements shape not only the maturation and architecture of aSyn inclusions but also their impact on organelle function and neuronal integrity over time.

### aSyn pathology disrupts lysosomal function in iDA

Previous studies using advanced techniques such as immunohistochemistry (*76*), proteomics (*77*–*79*), stimulated emission depletion (STED) microscopy (*80*), and CLEM (*15*, *17*, *81*, *82*) have provided robust evidence that endolysosomal organelles are sequestered within LB inclusions in postmortem human brain tissues. Proteomic analyses have identified numerous endolysosomal proteins, including those involved in vesicular trafficking and lysosomal function, reinforcing the role of endolysosomal pathways in LB pathology. Lysosomal markers, such as LAMP1, colocalize with aSyn aggregates, while ultrastructural analyses reveal lysosomal vesicles integrated within LB inclusions. These findings suggest a direct sequestration of endolysosomal compartments into LB, indicating their active recruitment into these pathological structures. This sequestration is believed to impair endolysosomal trafficking, leading to lysosomal dysfunction and subsequent cellular stress, as well as disrupted neuronal homeostasis. To determine whether our cellular model also recapitulates this aspect of the pathology, we investigated how the formation and maturation of LB-like inclusions in PFF-seeded iDA neurons affect lysosomal distribution and function.

First, we assessed the extent to which lysosomes were sequestered within LB-like inclusions at D42 (Fig. 9, A and B). We immunostained lysosomes using LAMP2 and observed their accumulation in contact with, and within, pS129-positive (pS129^+^) aSyn inclusions, suggesting that lysosomes tend to cluster and accumulate in the core of the LB-like inclusion (Fig. 9, A and B).

To test whether sequestration affected lysosomal function, we used LysoTracker to label active acidic vesicles (*56*, *83*, *84*) and analyzed inside (Fig. 9, B and C, yellow line) and the fluorescence intensity distribution of LAMP2 and LysoTracker outside (Fig. 9, B to D, purple line) the pS129+ inclusions. Inside inclusions, LysoTracker did not spike with LAMP2, whereas outside, both signals peaked together (Fig. 9, C and D). This indicates that lysosomes trapped in inclusions are functionally impaired. Such disruption may reflect an overloaded degradation system but could also represent a protective mechanism by sequestering damaged components. Rupture of lysosomal membranes may then release enzymes that fragment or degrade fibrils, explaining the scarcity of long fibrils in LB cores.

We next quantified fluorescence (area under the curve) for LAMP2 and LysoTracker across iDA neurons. Outside the inclusions, lysosomes were more active, with LysoTracker intensity peaking simultaneously with LAMP2 (Fig. 9D). While total lysosome content (LAMP2 levels) was unchanged, LysoTracker intensity was significantly reduced inside inclusions and elevated outside (Fig. 9, F and G). These results indicate that lysosomes sequestered into aSyn inclusions become functionally impaired or inactive.

Consequently, lysosomes accumulate in the core of inclusions but lose activity, resulting in impaired clearance capacity, heightened neuronal stress, and accelerated neurodegeneration. Together, these findings underscore the importance of preserving lysosomal integrity and highlight it as a potential therapeutic target in synucleinopathies. Using STED microscopy, we observed a significantly higher lysosomal density within the LB-like inclusions than in the surrounding cytoplasm, suggesting a concentration of lysosomal vesicles within the inclusions. Moreover, lysosomes sequestered within the LB-like inclusions exhibited reduced activity, indicating a significant functional impairment (Fig. 9, C to F). This reduction in lysosomal activity within LB-like inclusions implies that lysosomal dysfunction may disrupt cellular clearance processes, increasing neuronal stress and contributing to progressive neuronal dysfunction. Such impairment likely accelerates neurodegenerative progression, underscoring the critical role of lysosomal integrity in maintaining neuronal health and highlighting potential therapeutic avenues to target lysosomal preservation in synucleinopathies.

## DISCUSSION

In this study, we used a human isogenic iDA neuronal model, combined with a PFF seeding approach, to resolve the sequential stages of LB development and maturation in a context that recapitulates human brain pathology without relying on aSyn overexpression, genetic manipulation, additional cellular stressors or artificial constructs that might perturb the fibril interactome (e.g., GFP-αSyn fusion proteins) (figs. S14 and S15). We show that this physiologically relevant system captures the morphological, biochemical, and ultrastructural spectrum of aSyn aggregates observed in PD (*1*, *14*, *15*, *17*, *76*, *85*).

One key advantage of our iDA model is that it consistently generates both neuritic and somatic inclusions, including diffuse, dense and ring-like LB-like structures. This dual-compartment resolution enables direct, spatially resolved analysis of aggregate formation and progression within the same human neuron system. This is in contrast to most published iPSC-derived seeding model studies, in which filamentous or ribbon-like somatic aggregates, in addition to neuritic pathology, have been observed in only a small subset of studies (*29*, *30*, *39*, *58*).

Furthermore, in this model, we observed a wide spectrum of seeded aSyn pathology, ranging from early neuritic filamentous aggregates to dot-like, speckled, and filamentous inclusions, as well as dense and ring-like LB-like inclusions within the soma. This broad phenotypic range mirrors the diversity of Lewy pathology observed in postmortem PD, LBD, and MSA brain tissue and allows the study of both early seeding events and late-stage inclusion maturation processes.

**Fig. 10.**
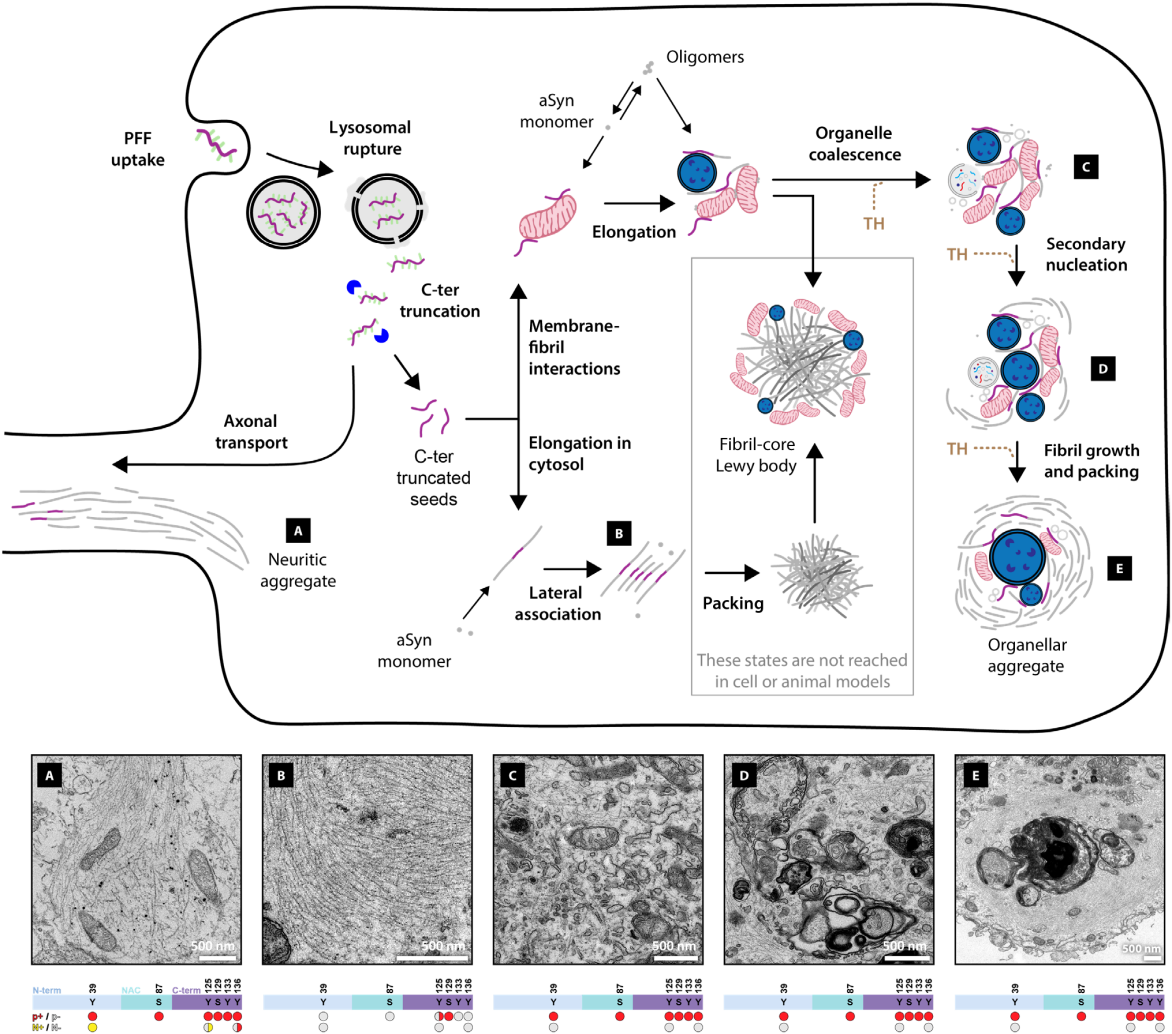
Mechanistic model of aSyn aggregation and inclusion diversity. Schematic representation of aSyn pathology formation integrating data from this study, prior seeding models, and recent EM analyses of LBs from human postmortem brains in PD and LBD. Following internalization into iDA neurons, PFF undergo rapid C-terminal truncation, predominantly at residue 114. These cleaved seeds recruit endogenous monomeric aSyn, initiating fibril elongation and amplification. Two distinct pathways are proposed: a membrane/organelle-independent (**A** and **B**) and a membrane/organelle-dependent mechanism (**C** to **E**), whose relative contribution likely depends on monomer availability and cellular context. In the membrane-independent route, fibrils elongate in the cytosol through monomer addition, undergo specific PTMs, and cluster into dense fibrillar assemblies. Neuritic aggregates (A) are primarily generated via this pathway following transport of truncated seeds to neurites. In the soma, when the kinetic of fibril growth exceeds organelle recruitment, compact inclusions with laterally associated fibrils form. These purely fibrillar inclusions may represent precursors of densely packed fibril-core LBs with radiating fibrils and peripheral organelles; however, such radiating structures have not been observed in seeding models, and this remains speculative. Conversely, in the membrane-dependent route [(C) to (E)], truncated seeds associate with intracellular membranes, where fibrils elongate and incorporate lipids. These lipid-rich fibrils scaffold organelle accumulation, generating membrane- and organelle-dense inclusions. Variability in fibril morphology, PTMs, packing, and organelle content likely reflects differences in seeding dynamics, maturation state, aberrant processing, and incorporation of TH. Fibrils within mature inclusions are consistently shorter than those observed earlier or peripherally, suggesting fragmentation by organelle-derived proteases. While this may facilitate degradation, impaired clearance could permit accumulation of seeding-competent fragments and promote propagation. LB-like inclusions may therefore act as reservoirs under proteostatic failure. This model captures seeded fibril amplification and inclusion maturation but does not encompass early oligomerization events, which may represent parallel or precursor pathways to aSyn fibrillization and LBs formation. Scale bars, 500 nm.

Beyond confirming known features of pathology, the model uncovers mechanistic insights into the mechanisms underpinning the diversity of aSyn-induced neurodegeneration, pathology formation, and propagation. Using a combination of imaging approaches and molecular profiling, we followed the development of aSyn pathology from early seed uptake to the formation of mature LB-like structures. While relying on pS129 antibodies, which have been the main pathology detection tools in all previous iPSC-derived seeding models, facilitates the detection of aSyn pathological diversity (*25*, *28*, *40*–*42*, *44*, *56*, *58*, *63*), it allows only limited insight into the biochemical diversity of aSyn aggregates across different inclusions and at different stages of LB formation. To address this, we performed systematic multiparametric immunostaining using additional hallmark markers of human LB pathology, including p62, ubiquitin, ThS, and a broad panel of antibodies against disease-associated PTMs and different organelles (fig. S16). This expanded molecular profiling allowed us to define distinct PTM, organelle, and ultrastructural signatures associated with specific inclusion morphologies and maturation stages. Together, our work shows that LB formation is not the result of a passive accumulation of fibrils, but rather a dynamic, progressive process driven by tightly coordinated events, in which the pattern of aSyn PTMs, together with fibril interactions with organelles and other intracellular components, drives the morphological and biochemical heterogeneity of aSyn pathology. Longitudinal analysis at D21 and D56 revealed a reproducible progression from simple filamentous structures to more complex cortical-like, ring-like, and compact LB-like morphologies, accompanied by the accumulation of disease-associated PTMs and pathological markers (Fig. 10). Although we did not follow individual inclusions over time, the consistency of these patterns suggests a stepwise maturation process reminiscent of LB development in vivo.

In this context, it is informative to consider prior reports of LB-like pathology in iPSC-derived neurons. To date, only two studies have described such structures following PFFs treatment. Lam *et al.* (*32*) used iPSC-derived cortical neurons expressing aSyn–green fluorescent protein (GFP) to study the ultrastructure of somatic inclusions. These inclusions contained fibrils, clustered vesicles, lipid droplets, dysmorphic mitochondria, and filamentous material, features reminiscent of human LBs. The authors classified multiple inclusion types, including neuroprotective p62-positive structures and lipid-rich, neurotoxic inclusions. While the use of aSyn-GFP fusion constructs facilitates the detection of aSyn aggregates and monitoring their fate, it is also known to alter fibril surface properties and cellular interactions, potentially affecting inclusion morphology and composition (*86*, *87*). In previous neuronal seeding models expressing aSyn-GFP fusion constructs, the inclusions were found to be composed primarily of fibrillar aggregates (*88*). In parallel, Bayati *et al.* (*44*) demonstrated that LB-like inclusions could form in iDA neurons treated with aSyn PFFs but only under immune challenge with interferon-γ. In their model, lysosomal impairment facilitated PFF uptake and aggregate accumulation within membrane-bound compartments, proposed to result from autophagosome expansion due to chaperone-mediated autophagy dysfunction (*44*, *89*). However, these membrane-enclosed inclusions contrast with the membraneless LB observed in human tissue (*17*, *75*, *90*–*94*), except in rare neuritic cases (*15*). These membrane-enclosed inclusions were identified primarily through immunogold labeling of the PFF, without molecular or ultrastructural confirmation of their correspondence to authentic LB pathology resulting from newly formed aggregates.

### PTMs shape the biochemical architecture and identity of aSyn pathology

Our iDA seeding model reveals that distinct aSyn PTMs are selectively enriched across distinct aggregate subtypes and neuronal compartments. Moreover, our results reveal that neuritic and somatic aSyn pathology exhibit distinct aSyn PTM patterns. Mature LB-like inclusions displayed the most complex PTM signatures, whereas early-stage or filamentous aggregates showed minimal modification. This suggests either a progressive accumulation of PTMs during aSyn aggregate formation and maturation or that specific PTM patterns may influence the kinetics of pathology formation and the types of aggregates that emerge. The detection of fibrillar aggregates that exhibit minial PTMs also suggests that many of the PTMs could occur post aSyn fibrillization. In neurons that simultaneously contain LB-like inclusions and adjacent speckled aggregates, only the LB-like structures are PTMs positive, while speckled aggregates remain negative, demonstrating selective PTM incorporation within the same cellular environment. These findings support the idea that PTM profiles not only reflect the stage of aggregate maturation but also vary by subcellular localization and possibly by the structural identity of the aggregate itself. These compartment-specific PTM profiles likely have functional consequences.

Previous STED microscopy of postmortem human PD tissue has shown that PTM-modified aSyn species, such as pS129 and N- and C-terminal truncations, form concentric, layered “onion-skin” architectures within a subset of aSyn inclusions (ring-like structures) (*80*). This structural stratification suggests that PTMs are not merely post hoc consequences of aggregation but may actively regulate the progression of inclusion assembly. These patterns imply the existence of distinct biochemical microenvironments within aggregates, potentially governed by local kinase/phosphatase activity, protein quality control mechanisms, or oxidative stress. In line with this, prior studies have found that early-stage aggregates are enriched in PTMs, such as pS129 and nY39, while later-emerging modifications, such as pS87 and pY136, are associated with more mature inclusions (*95*). Our data mirror this progression, with complex PTM profiles accumulating in compact, late-stage aggregates and early inclusions remaining largely unmodified (Fig. 10). Recent studies from our group demonstrate that PTMs can also modify the biochemical behavior and pathogenic potential of aSyn aggregates, with modifications such as O-GlcNAc modification (*52*) or nitration (*69*) nearly abolishing the seeding activity of aSyn fibrils in vivo. pS129 was the only PTM consistently detected across all aggregate morphologies and compartments, including dot-like, speckled, filamentous, neuritic, and LB-like structures. This positions pS129 as a central hallmark of fibrillar aSyn pathology and the most reliable marker for capturing the full spectrum of seeded fibrillar aggregates (excluding nonfibrillar oligomers). Its ubiquitous presence suggests that pS129 is deposited early, potentially during or immediately after fibrillization, while other PTMs appear selectively and correlate more strongly with inclusion maturation and subcellular context.

### A mechanistic model of aSyn pathology formation and diversity

On the basis of our observations, we propose a working model for the mechanisms of aSyn seed–mediated pathology formation that could partially explain the pathological diversity observed in human PD brains (Fig. 10). Upon internalization of PFF seeds into neurons, through the endolysosomal pathway, they undergo rapid cleavage of the last 25 C-terminal residues, which increases the hydrophobicity of the fibril surfaces and facilitates both primary and secondary nucleation events at the fibril ends and surfaces, respectively. At this stage, aSyn aggregation can proceed through at least two pathways: a membrane-independent mechanism and a membrane-dependent mechanism.

#### Membrane-independent pathway

Fibril elongation through monomer addition, combined with PTMs incorporation and further C-terminal cleavages, generates fibrils with a strong propensity for lateral association and rapid clumping into dense fibrillar assemblies. These assemblies are distinguished by cores enriched in truncated aSyn species and peripheries enriched in full-length and phosphorylated aSyn. Membranous organelles, such as mitochondria, are subsequently recruited to the periphery. Notably, this process occurs in both neurites and soma; however, the PTM patterns of neuritic fibrils differ from those of organelle-free fibrillar accumulations in the soma, suggesting compartment-specific interactomes and differential fibril processing.

#### Membrane-dependent pathway

Growing evidence indicates that, upon internalization into neurons, aSyn fibrils associate with various membranous organelles, including mitochondria. Fibril elongation on organelle surfaces promotes the formation of longer fibrils that may act as scaffolds for the recruitment of additional membranous organelles. Thus, it is plausible that fibril interactions with different organelles may trigger distinct endogenous fibril seeds and pathways to fibrillization and LB formation.

Our CLEM studies suggest that organelle sequestration in the vicinity and center of LBs is not a passive consequence of aggregation but appears highly selective and compartment specific (Fig. 10). Mitochondria were consistently recruited across inclusion types, but their distribution varied: They often accumulated at the periphery of pS129-positive inclusions, a pattern also reported in brain tissue of patients with PD (*17*). Lysosomes, in contrast, were found exclusively in more mature, LB-like aggregates, implying that lysosomal engagement is a late-stage event, potentially reflecting an overwhelmed or dysfunctional autophagic response. The Golgi apparatus and endosomes were recruited more broadly, including to earlier-stage aggregates, suggesting that secretory and trafficking pathways may be disrupted from the onset of seeded pathology. A particularly compelling feature of the ring-like mature LB-like inclusions is the presence of electron-dense MLBs in their cores, consistent with previous reports in Alzheimer’s disease (*73*) and PD models (*44*, *62*) where they arise under sustained mitochondrial and lysosomal stress (*44*, *62*, *72*).

At the same time, we note that MLBs have not been directly observed in somatic, filamentous LBs in postmortem studies. However, the centers of bona fide ring-shaped LBs are known to be lipid rich (*96*, *97*), electron dense (*75*), and surrounded by fibrils, three features shared with the MLB core of ring-like inclusions in our cell model. Although we have no evidence that MLBs represent a precursor stage of dense-core LBs, which may take years to develop in the human brain, their similar biochemical composition (i.e., enrichment in lipids and mitochondrial and autophagosomal markers) and their central position within the aSyn-positive ring suggest that these two structures may be related. Moreover, their selective restriction to the inclusion core suggests that MLBs represent a late-stage hallmark of LB maturation, reflecting combined mitochondrial and lysosomal dysfunctions. Together, we propose that the differential recruitment and fate of organelles contribute to the distinct morphologies of aSyn pathological aggregates observed across disease contexts (Fig. 10).

Moreover, it is plausible that fibrils formed through the membrane-dependent pathway differ in structure, biochemical composition (PTMs and lipids), and secondary nucleation properties from those formed via the membrane-independent pathway. Previous studies have shown that fibril growth on membrane surfaces can disrupt membranes and sequester lipid molecules, which then become incorporated into the fibrils. Lipid incorporation could alter fibril structure and biochemical properties, potentially explaining why speckled-like aggregates are devoid of PTMs (e.g., ubiquitination) and do not bind ThS. A consistent observation across our CLEM studies is that fibrils located in the core of membranous organelle–enriched inclusions are markedly shorter than those at the periphery of inclusions or in organelle-free neuritic and somatic fibril bundles. This suggests that core fibrils may undergo fragmentation driven by proteolytic enzymes released from disrupted lysosomes. Since fibril size likely influences their ability to be secreted and internalized, and smaller fibrils exhibit higher internalization and seeding activity, these findings further link LB formation to the generation of seeding-competent small fibrils. Together, this supports the idea that blocking the transition from fibrils to LBs could hinder the propagation of aSyn pathology.

Together, our mechanistic model (Fig. 10) provides a framework for testing how different pathways of fibril growth, remodeling, and fragmentation contribute to the diversity of aSyn pathology. This framework can be refined and expanded through future studies that integrate high-resolution imaging, biochemical analysis, and structural characterization of both model-derived and human brain–derived fibrils (*13*). In this context, it will be particularly important to address how the exposed N- and C-terminal domains of fibrils regulate interactions with cellular proteins and organelles and how PTMs within these domains influence pathology formation and spreading. Developing new neuronal models that express, at physiological levels, N- and C-terminal variants designed to alter fibril-membrane or fibril-organelle interactions could provide critical insights into the mechanisms that shape aSyn aggregation, inclusion maturation, and toxicity.

These ultrastructural organization of the various Lewy aggregates raise a broader question in synucleinopathy research: whether LBs serve primarily harmful or protective functions. Our findings support a dual perspective. On one hand, the accumulation of mitochondria, lysosomes, and endosomes within inclusions coincides with structural disruption and loss of organelle function, most clearly demonstrated by our lysosomal functional assays. Lysosomes trapped inside inclusions exhibit significantly reduced acidification and functional impairment, suggesting that their sequestration compromises degradation capacity and may exacerbate cellular stress. Likewise, mitochondrial damage was more pronounced in neurons with inclusions, with a subset of severely damaged mitochondria observed outside of aggregates, potentially representing organelles that had not yet been engulfed or processed into MLBs. On the other hand, the selective compartmentalization of dysfunctional organelles into inclusions could reflect a protective mechanism aimed at shielding the surrounding cytosol from further harm. This aligns with models proposing that LB function to sequester potentially harmful or damaged cellular components. However, as our data show, this sequestration comes at a cost: Lysosomal function is compromised, organelle turnover is disrupted, and essential proteins and enzymes like TH become mislocalized and likely rendered nonfunctional because of their confinement within inclusions and reduced accessibility within the cytoplasm. It is reasonable to propose that the kinetics and efficiency of LB formation could determine whether this process is protective or toxic. Rapid and efficient LB formation, leading to the sequestration of toxic proteins and dysfunctional organelles, could be protective, whereas delayed or stalled LB formation would allow the accumulation of toxic aggregates and damaged organelles, thereby exacerbating toxicity. Further studies are needed to test this hypothesis.

In conclusion, our study establishes the iDA seeding model as a physiologically relevant human neuron system that resolves the sequential stages of Lewy pathology formation without genetic manipulation or aSyn overexpression. In this model, inclusions emerge through progressive incorporation of PTMs, selective recruitment of mitochondria, lysosomes, and other organelles, and the late appearance of multilamellar bodies, together defining a stepwise maturation process. These observations indicate that inclusion remodeling is an active process that can both compartmentalize damaged components and, at the same time, compromise essential cellular functions. By capturing these dynamic and compartment-specific events, the iDA model provides a framework for dissecting the mechanisms that shape aSyn pathology, deciphering the relationship between aSyn pathology formation and neurodegeneration and for testing targeted approaches to modulate its maturation and impact on neuronal health.

## MATERIALS AND METHODS

### Purification of human WT aSyn

Recombinant overexpression and purification of human WT aSyn were carried out using *Escherichia coli* BL21 (DE3) cells transformed with pT7-7 plasmids encoding WT aSyn. After growing the cells in Luria broth medium with ampicillin, protein expression was induced with 1 mM 1-thio-β-d-galactopyranoside when the optical density at 600 nm reached 0.4 to 0.6. The culture was incubated for 4 to 5 hours before the cells were harvested by centrifugation. Cell pellets were lysed using ultrasonication, and the lysate was subjected to heat treatment followed by centrifugation to remove impurities. The resulting supernatant was purified using a HiPrep Q Fast Flow column, and fractions containing pure aSyn were analyzed by SDS–polyacrylamide gel electrophoresis (SDS-PAGE), pooled, and further purified using reversed-phase high-performance liquid chromatography as previously described (*51*, *53*). The final product was confirmed to be highly pure through ultraperformance liquid chromatography and electrospray ionization mass spectrometry, then snap frozen and lyophilized for storage.

### Preparation of WT aSyn monomers

The lyophilized WT aSyn was resuspended in PBS buffer and processed according to a previously described filtration method (*51*, *53*). The resuspended aSyn was adjusted to a pH of approximately 7.2 to 7.4 and filtered using 100-kDa spin filters. The filtrate containing the monomeric form of aSyn was quantified by measuring the absorbance at 280 nm using a NanoDrop 2000 Spectrophotometer (Thermo Fisher Scientific) and an extinction coefficient of 5960 M^−1^ cm^−1^, as predicted from the aSyn sequence (ProtParam, ExPASy). Monomeric WT aSyn was prepared for use in subsequent experiments.

### Preparation of WT aSyn PFF

WT aSyn PFFs were prepared by dissolving 4 mg of lyophilized recombinant aSyn in 600 μl of 1× PBS (*51*, *53*). The pH was adjusted to approximately 7.2 to 7.4, and the solution was filtered through 0.2-μm filters (MERCK, SLGP033RS). The filtrate was transferred to black screw-cap tubes, which were incubated at 37°C and shaken at 1000 rpm for 5 days. Fibril formation was verified by TEM and Coomassie staining, as previously described (*51*, *53*). For sonicated seed preparation, WT aSyn PFFs were sonicated for 20 s at 20% amplitude, with 1-s pulses on and off (Sonic Vibra Cell, Blanc Labo, Switzerland). Following established protocols, the amount of monomers and oligomers released from the sonicated fibrils was quantified through filtration. TEM confirmed the fibril structure, and Coomassie staining was used to quantify the release of monomers and oligomers following sonication (*51*, *53*).

### Preparation of WT aSyn PFFs fluorescently labeled

Fluorescent labeling of human WT aSyn PFFs was performed as previously described (*53*) by diluting PFFs to a concentration of 250 μM in 500 μl of PBS, with the pH adjusted to 7.5. An equivalent amount of Atto488 or Atto647 maleimide (Atto-Tec, Switzerland) was added, and the mixture was incubated overnight at 4°C. After incubation, the labeled PFFs were ultracentrifuged at 100,000*g* for 1 hour at 4°C. The supernatant was discarded, and the pellet was resuspended in PBS, repeating the wash steps until the dye in excess was removed. Labeling was confirmed by running the fibrils on SDS-PAGE and scanning the gel using the Typhoon FLA 7000 (GE Healthcare) with excitation and emission wavelengths of 400 and 505 nm, respectively. The labeled fibrils were fragmented through sonication, using 4 cycles of 5 s at 20% amplitude (Sonic Vibra Cell, Blanc Labo, Switzerland). Last, the PFFs were snap frozen in liquid nitrogen and stored at −80°C. The structural and biophysical properties of the fibrils were evaluated using EM, SDS-PAGE with Coomassie staining, and thioflavin T assays.

### Differentiation of NGN2-iPSCs into iDA neuronal culture 

The optimized protocol for generating functional iDA cultures has been fully described by Sheta *et al.* (*49*, *50*). Briefly, the NGN2-expressing human iPSCs (AIW002-02, from The Early Drug Discovery Unit, McGill University, Canada) were first differentiated into iNeurons. On day −1, cells were plated in mTESR plus media (STEMCELL Technologies, Switzerland) containing Y27632 (Tocris, Switzerland). The next day (DIV0), the mTESR plus media was fully replaced by the complete DIV0/DIV1 media composed of Dulbecco’s modified Eagle’s medium (DMEM)/F12 (Life Technologies, Switzerland), N2 (Life Technologies, Switzerland), B27 (Life Technologies, Switzerland), Non-Essential Amino Acids (NEAA) (Life Technologies, Switzerland), brain-derived neurotrophic factor (BDNF) (10 ng/ml) (450-02; PeproTech, UK), glial cell line–derived neurotrophic factor (GDNF) (10 ng/ml) (450-10; PeproTech, UK), mouse laminin (23017015; Life Technologies, Switzerland), and doxycycline (2 μg/ml) (Sigma-Aldrich, Switzerland) to induce NGN2 expression. On DIV1, the media was fully changed again, using the complete DIV0/DIV1 media as described above. At DIV2, the cells were dissociated with Accutase and replated on dishes (96- or 48-well black clear-bottom plates) or on coverslips coated with poly-l-ornithine (Sigma-Aldrich, Switzerland) and laminin (23017015; Life Technologies, Switzerland) in a Neurobasal medium supplemented with N2, B27, GlutaMax, NEAA, BDNF (10 ng/ml), GDNF (10 ng/ml), mouse laminin. and doxycycline (2 μg/ml). The day after (DIV3), the NGN2-expressing iNeurons were exposed to differentiation media from the STEMdiff Midbrain Neuron Differentiation Kit (STEMCELL Technologies, Switzerland), supplemented with doxycycline (2 μg/ml) and Sonic Hedgehog (200 ng/ml). At DIV 6, the media was fully changed with the complete STEMdiff Midbrain Neuron Differentiation supplemented with doxycycline (2 μg/ml) and Sonic Hedgehog (200 ng/ml). At DIV 9, the media was replaced with maturation media from the STEMdiff Midbrain Neuron Maturation Kit (STEMCELL Technologies, Switzerland). From DIV9 onward, half of the media was replaced weekly with fresh maturation media. All procedures were approved by the Swiss Cantonal Ethics Committee on Research Involving Humans (CER-VD, Commission cantonale d’éthique de la recherche sur l’être humain), approval number 2025-00664.

### PFF treatment

The iDA cultures were plated in 35-mm fluorodishes (Millian, France), either with or without coverslips (VWR, Switzerland), at a density of 500,000 cells/ml. iDA cultures were plated in black, clear-bottom 96-well plates (Falcon, Switzerland) at a density of 100,000 cells/ml. On DIV9, the differentiation media was completely replaced with maturation media. This media either contained aSyn PFF at a final concentration of 500 nM, or an equivalent volume of PBS, which matched the amount of PFF added to the extracellular media (Table 1).

**Table 1.** Cell densities and media volumes used for PFF treatment. Summary of plating conditions for experiments using 500 nM PFFs. The table lists the required cell density and final media volume per well for 35-mm fluorodishes and black/clear flat-bottom 96-well plates.

PFF treatment (500 nM final)	Cell density (cells/ml)	Final volume of media per well
35-mm fluorodishes	500,000	1.5 ml
Black/clear flat-bottom 96-well plates	100,000	100 μl

### Hippocampal primary neuronal culture

Pregnant C57BL/6Jrj mice (Janvier Labs, France) were imported on demand at the EPFL animal facility. Neonatal mice (postnatal days 0 to 1) were euthanized by decapitation immediately before brain dissection, and primary hippocampal neurons were isolated following established protocols (*53*). All procedures were performed ex vivo. In alignment with the 3Rs principle, dams were, when appropriate, redirected to the EPFL Organ/Tissue Sharing Program (Optimice). All animal protocols received approval from the Swiss Federal Veterinary Office (authorization numbers VD3392 and VD4029). Cells were plated in six-well plates precoated with 0.1% (w/v) poly-l-lysine in water (Brunschwig, Switzerland), at a density of 300,000 cells/ml.

### Total cell lysis and WB analyses

iDA cultures were lysed using 2% SDS in tris-buffered saline [50 mM tris and 150 mM NaCl (pH 7.5)] containing the protease inhibitor cocktail (Roche, Switzerland), 1 mM phenylmethane sulfonyl fluoride, and phosphatase inhibitor cocktails 2 and 3 (Sigma-Aldrich, Switzerland). The cell lysates were boiled for 10 min, and protein concentration was determined using the bicinchoninic acid assay (BCA) protein assay (Thermo Fisher Scientific, Switzerland). Laemmli buffer [4% SDS, 40% glycerol, 0.05% bromophenol blue, 0.252 M tris-HCl (pH 6.8), and 5% β-mercaptoethanol] was added to the lysates, and 30 μg of total protein was loaded onto 16% tricine gels for 2 hours at 125 V. Proteins were then transferred to 0.2-μm nitrocellulose membranes (GE Healthcare, Switzerland) using a semidry transfer system (Life Technologies, Switzerland) at 25 V and 0.5 A. Membranes were blocked for 1 hour at room temperature (RT) in Odyssey blocking buffer (Li-COR Biosciences, Germany), followed by overnight (O/N) incubation at 4°C with primary antibodies (listed in the tables in fig. S1). After three washes in PBS with 0.1% Tween 20, the membranes were incubated with secondary antibodies (Alexa Fluor 680 or 800, Li-COR Biosciences). The membranes were washed again with PBS with 0.1% Tween 20 and scanned using a Li-COR scanner (Li-COR Biosciences, Germany). Total aSyn levels were quantified by analyzing the WB band intensity using Image Studio software (RRID:SCR_015795, Li-COR Biosciences, Germany) and normalized to actin levels. All experiments were performed in triplicate independently.

### Immunocytochemistry

iDA cultures were fixed with 4% formaldehyde (Sigma-Aldrich, Switzerland) for 20 min at RT, followed by immunostaining as previously described (*51*, *53*). Details regarding the antibodies used, including their source and dilution, are provided in each figure legend and fig. S1. In brief, the iDA cultures were blocked with 3% bovine serum albumin (BSA, Sigma-Aldrich, Switzerland) in PBS containing 0.1% Triton X-100 (PBS-T) for 30 min at RT. After blocking, the cells were incubated overnight at 4°C with primary antibodies. Following five washes in PBS-T, the cultures were incubated with secondary antibodies and stained with 4′,6-diamidino-2-phenylindole (DAPI) (Life Technologies, Switzerland). The cells were again washed five times with PBS-T before being mounted using polyvinyl alcohol mounting medium with 1,4-diazabicyclo[2.2.2]octane (DABCO) (Sigma-Aldrich, Switzerland).

Cells plated on coverslips were examined using a confocal laser scanning microscope (LSM 700, Carl Zeiss) with a 40× objective, and images were analyzed with Zen software (RRID:SCR_013672). For iDA cultures grown in black, clear-bottom 96-well plates, imaging was performed using the IN Cell Analyzer 2200, as previously described (*51*, *53*).

### Assessment of PFF uptake in iDA neurons by live-cell imaging

At DIV10, iDA neurons were stained with 1 μM LysoTracker and NeuroFluor NeuO (StemCell, catalog no. 01801) diluted 1:400 in BrainPhys Imaging Optimized Medium (StemCell, catalog no. 05796) for 45 min. Following staining, the medium was replaced with fresh BrainPhys Imaging Optimized Medium containing 300 nM of labeled PFF^647^). Cells were placed in a stage-top incubator (Tokai Hit) on the microscope, with CO_2_ maintained at 5% and temperature at 37°C. After 20 min of incubation, time-lapse imaging was initiated using an inverted Leica TCS SP8 STED 3X microscope in confocal xyzt mode, equipped as previously described, with the following modifications: HC PL APO CS2 63×/1.40 oil objective, 4.5× numerical zoom, 1024 × 1024 pixel resolution, and bidirectional scanning at 1400 Hz with three-line averaging. Two sequential z-stacks were acquired with 1-μm steps (5-μm total thickness) to capture all images for both sequences within 17.389 s (8.69 s per stack for each sequence). Continuous imaging was performed after each stack acquisition for up to 10 min. Fluorescence was recorded using hybrid detectors (HyD) with these settings: First sequence (dual-color acquisition): NeuO (3% laser power at 480 nm, fluorescence collected at 485 to 550 nm, gain 80 V, and 0- to 3.50-ns time gating) and PFF-AF647 (3% laser power at 645 nm, fluorescence collected at 651 to 720 nm, gain 130 V, and 0- to 3.50-ns time gating). Second sequence (single-color acquisition): LysoTracker (2.5% laser power at 570 nm, fluorescence collected at 578 to 650 nm, gain 80 V, and 0- to 3.50-ns time gating).

### Quantification of total aSyn endogenous level in iDA cultures at DIV9

iDA cultures were fixed at DIV9 in 4% formaldehyde (Sigma-Aldrich, Switzerland) for 20 min at RT, immunostained as described in the “Immunocytochemistry” section above, and acquired using IN CellAnalyser 2200 (GE Healthcare). For each independent experiment, three wells were acquired per condition, and in each well, nine fields of view were imaged. Each independent experiment was reproduced at least three times. The identification of TH-positive or TH-negative neuronal cells and the quantification of aSyn intensity were performed using a custom-built Macro in Fiji ImageJ (RRID:SCR_002285). In brief, cells were thresholded on the basis of their TH fluorescence signal, and nuclei were thresholded on the basis of DAPI staining. Then, cells were separated from each other using a marker-controlled watershed with the center of the nuclear detections as seeding points and the binarized TH channel as a mask. Cells with incorrectly differentiated neurites were manually excluded from the analysis. Within each cell, the median gray value of the aSyn fluorescence channel was used as a representative measure of the aSyn level in that cell. Mean fluorescence intensity per cell was divided by the corresponding cell area and normalized to the population mean of each independent experiment, then visualized as a violin plot.

### Correlative light electron microscopy

iDA cultures were grown in 35-mm fluorodishes (Millian, France) onto which identifying numbers and letters were manually engraved onto the glass underside. The cells were treated with either PBS (as a negative control) or 500 nM human WT PFF or PFF^488^. At the specified time point, cells were fixed for 2 hours using 4.0% PFA in 0.1 M phosphate buffer (PB) at pH 7.4. Instead of using detergent for permeabilization, a freeze-thaw method was used to better preserve cellular membranes and organelles. Briefly, the fixed cells were incubated for 20 min in a cryoprotectant solution [20% dimethyl sulfoxide and 2% glycerol in 0.1 M PB (pH 7.4)]. Then, after removing the cryoprotectant, each dish was quickly plunged into liquid nitrogen (LN2) for about 7 s, then removed, and refilled with cryoprotectant to unfreeze the frozen cells. This freeze-thaw process was repeated twice. After washing with PBS, ICC was performed (refer to the “Immunocytochemistry” section for more details). TH-positive neurons containing LB-like inclusions (positive for pS129 staining) were selected using a confocal fluorescence microscope (LSM700, Carl Zeiss, Germany) for ultrastructural analysis. The precise location of selected cells was recorded using the position of the numbers and letters scratched on the underside of the dish. Cells were then processed for EM using a slightly modified previously published protocol (*53*). Briefly, cells were fixed again with 2.5% glutaraldehyde and 2.0% paraformaldehyde in 0.1 M PB at pH 7.4 for an additional 2 hours and postfixed for 40 min in 1% osmium tetroxide and 0.8% potassium ferrocyanide in cacodylate buffer (0.1 M pH 7.4), followed by 0.2% tannic acid in the same buffer. They were then stained with 1% uranyl acetate in an acetate buffer, at pH 5.2 for 1 hour, and lastly dehydrated through a graded ethanol series, and embedded in Durcupan resin. After polymerization, the resin-embedded cells were trimmed, sectioned (50 to 60 nm), and mounted on single-slot copper grids with a pioloform support film. These thin sections were contrasted with uranyl acetate and lead citrate, imaged in a transmission electron microscope (Tecnai Spirit, FEI) operated at 80 kV, and imaged captured with a charge-coupled device camera (Eagle, FEI).

### Immunogold staining

To validate the presence of fibrillar aSyn within aggregates, pre-embedding immunogold labeling was performed. After PFA fixation and incubation in primary antibodies (see above), cells were incubated with FluoroNanogold-conjugated secondary antibody (Nanoprobes) for 2 hours at RT, and immunofluorescence microscopy, resin embedding, ultramicrotome sectioning, and TEM imaging were performed as described above. On the resulting EM micrographs, gold particles were detected using a difference-of-Gaussian filter implemented in Fiji ImageJ (RRID:SCR_002285).

### Tomography

To measure fibril properties, tilt series from −60° to 60° were acquired on resin-embedded slices using a transmission electron microscope (Tecnai T12, FEI) at 120 kV. Tomograms were reconstructed using IMOD software. Small training sets of gold-associated, thick filaments (class 1) and thin filaments without gold particles attached to it (class 2) were segmented manually, and two convolutional neural networks were trained in EMAN2 to segment both classes separately in the whole tomogram. In FIJI, the thickness of the fibrils was measured by making a three-dimensional distance map of the fibril segmentations, which was evaluated at the pixels that constitute the skeletonization of the segmentations.

### Super-resolution STED imaging

For super-resolution STED microscopy, iDA neurons on coverslips were fixed 52 days post-PFF exposure using 3% PFA and 0.1% glutaraldehyde for 15 min at RT. After fixation, neurons were washed three times in PBS (5 min each) and permeabilized with 0.3% Triton X-100 (Sigma-Aldrich, Canada) for 5 min at RT. Following a PBS wash, neurons were incubated in blocking solution (5% normal goat serum, 1% BSA, and 0.1% Triton X-100 in PBS) for 1 hour, then with primary antibodies in the blocking solution overnight at 4°C (refer to the antibody table (fig. S1). The next day, neurons were washed three times with blocking solution (5 min each) and incubated with secondary antibodies for 1 hour (STAR635P for LAMP2 and AF594 for pS129; see the antibody table). After three additional PBS washes (each 5 min) and a brief rinse in Milli-Q water, coverslips were mounted using Prolong Diamond Antifade reagent (refractive index 1.47; Thermo Fisher Scientific, Canada).

Imaging was performed on an inverted Leica TCS SP8 STED 3X microscope, featuring a motorized stage, a tunable white-light laser (470 to 780 nm), and a 405-nm diode laser (Leica Biosystems, Concord, ON, Canada). Sequential scanning of each channel was done with a 405-nm laser (TH, Alexa Fluor 405), 590-nm laser (pS129, Alexa Fluor 594), or 656-nm laser (LAMP2, Abberior STAR 635P). Fluorescence depletion for Alexa Fluor 594 (pS129) and Abberior STAR 635P (LAMP2) was achieved using a 775-nm pulsed laser set at 30% input power, with tuning at 9 and 4.5% for pS129 and LAMP2, respectively. Laser intensities and gain settings were optimized to maximize the signal-to-noise ratio and prevent saturation using the QLUT Glow range indicator. Point scanning was conducted using a bidirectional scanner set to 700 Hz, with four-line averaging applied. A 100× HC PL APO CS2/1.40 NA STED white objective (Leica, Canada) with immersion oil type F (refractive index 1.5180, Leica, Canada) and a 2× numerical zoom was used. HyD collected the emitted wavelengths: 655 to 750 nm for LAMP2 (Abberior STAR 635P), 600 to 630 nm for pS129 (Alexa Fluor 594), and 410 to 500 nm for TH. The pixel size was set to 15 nm (2056 × 2056 pixels), and the pinhole size and z-step were optimized for resolution and oversampling, supporting later deconvolution. Deconvolution was performed using Huygens Professional (Scientific Volume Imaging, Hilversum, the Netherlands) with a theoretical point spread function, custom background settings, default signal-to-noise ratio, and the CMLE algorithm. Image adjustments (color balance, contrast, and brightness) were made using Fiji ImageJ (RRID:SCR_002285).

### Lysosomal functional assay

iDA neurons were exposed to PFF for 52 days and then stained with 1 μM LysoTracker DND-99 (Thermo Fisher Scientific, Canada) for 45 min in maturation medium (STEMcell, Canada). After staining, neurons were rinsed once with 1× PBS containing Ca^2+^ and Mg^2+^ and fixed with 3% PFA and 0.1% glutaraldehyde for 15 min at RT. Neurons were then immunostained as described in the previous section for super-resolution STED microscopy, with adjustments to the primary and secondary antibody concentrations (LAMP2/AF405, 1:100; pS129/AF488, 1:400; TH/AF594, 1:400).

Lysosomal clustering was analyzed using Fiji ImageJ (RRID:SCR_002285). Two regions within each neuron were delineated to define: (i) the area containing aSyn inclusions and (ii) the cytosolic region of the soma without inclusions. The corrected total cell fluorescence (CTCF) of LAMP2 (representing total lysosomes) was measured within both regions to evaluate lysosomal density inside and outside the inclusions. CTCF values were normalized to the surface area of each region (*n* = 3, total of 37 neurons). To assess lysosomal function, fluorescence intensity of LysoTracker and LAMP2 was measured along a line crossing lysosomes (LAMP2) inside aSyn inclusions and in the cytosolic region (*n* = 3, total of 19 neurons). The area under the curve for LysoTracker and LAMP2 fluorescence was calculated and normalized by distance using GraphPad Prism (RRID:SCR_002798).

### Statistics

Statistical analysis was conducted using GraphPad Prism (RRID:SCR_002798) on data from a minimum of three independent experiments. One-way analysis of variance (ANOVA) was used to evaluate the results, followed by Tukey’s post hoc test for multiple comparisons (details on the compared groups are provided in the relevant figure legends). A *P* value of less than 0.05 was considered statistically significant. One-way ANOVA followed by Dunnett’s multiple comparison test was used for fig. S3E, and an unpaired *t* test for Fig. 9.

## References

[1] M. Goedert, R. Jakes, M. G. Spillantini, The synucleinopathies: Twenty years on. J. Parkinsons Dis. 7, S51–S69 (2017). 28282814 10.3233/JPD-179005 PMC5345650

[2] T. Simuni, L. M. Chahine, K. Poston, M. Brumm, T. Buracchio, M. Campbell, S. Chowdhury, C. Coffey, L. Concha-Marambio, T. Dam, P. DiBiaso, T. Foroud, M. Frasier, C. Gochanour, D. Jennings, K. Kieburtz, C. M. Kopil, K. Merchant, B. Mollenhauer, T. Montine, K. Nudelman, G. Pagano, J. Seibyl, T. Sherer, A. Singleton, D. Stephenson, M. Stern, C. Soto, C. M. Tanner, E. Tolosa, D. Weintraub, Y. Xiao, A. Siderowf, B. Dunn, K. Marek, A biological definition of neuronal α-synuclein disease: Towards an integrated staging system for research. Lancet Neurol. 23, 178–190 (2024). 38267190 10.1016/S1474-4422(23)00405-2

[3] L. Concha-Marambio, S. Pritzkow, M. Shahnawaz, C. M. Farris, C. Soto, Seed amplification assay for the detection of pathologic alpha-synuclein aggregates in cerebrospinal fluid. Nat. Protoc. 18, 1179–1196 (2023). 36653527 10.1038/s41596-022-00787-3 PMC10561622

[4] A. Siderowf, L. Concha-Marambio, D. E. Lafontant, C. M. Farris, Y. Ma, P. A. Urenia, H. Nguyen, R. N. Alcalay, L. M. Chahine, T. Foroud, D. Galasko, K. Kieburtz, K. Merchant, B. Mollenhauer, K. L. Poston, J. Seibyl, T. Simuni, C. M. Tanner, D. Weintraub, A. Videnovic, S. H. Choi, R. Kurth, C. Caspell-Garcia, C. S. Coffey, M. Frasier, L. M. A. Oliveira, S. J. Hutten, T. Sherer, K. Marek, C. Soto, Parkinson’s Progression Markers Initiative, Assessment of heterogeneity among participants in the Parkinson’s Progression Markers Initiative cohort using α-synuclein seed amplification: A cross-sectional study. Lancet Neurol. 22, 407–417 (2023). 37059509 10.1016/S1474-4422(23)00109-6 PMC10627170

[5] T.-S. Fan, S.-C. Liu, R.-M. Wu, Alpha-synuclein and cognitive decline in Parkinson disease. Life 11, 1239 (2021). 34833115 10.3390/life11111239 PMC8625417

[6] C. H. Lin, S. Y. Yang, H. E. Horng, C. C. Yang, J. J. Chieh, H. H. Chen, B. H. Liu, M. J. Chiu, Plasma α-synuclein predicts cognitive decline in Parkinson’s disease. J. Neurol. Neurosurg. Psychiatry 88, 818–824 (2017). 28550072 10.1136/jnnp-2016-314857 PMC5629933

[7] H. Braak, K. Del Tredici, U. Rüb, R. A. de Vos, E. N. Jansen Steur, E. Braak, Staging of brain pathology related to sporadic Parkinson’s disease. Neurobiol. Aging 24, 197–211 (2003). 12498954 10.1016/s0197-4580(02)00065-9

[8] K. A. Jellinger, Lewy body-related α-synucleinopathy in the aged human brain. J. Neural Transm. 111, 1219–1235 (2004). 15480835 10.1007/s00702-004-0138-7

[9] T. G. Beach, C. H. Adler, L. Lue, L. I. Sue, J. Bachalakuri, J. Henry-Watson, J. Sasse, S. Boyer, S. Shirohi, R. Brooks, J. Eschbacher, C. L. White III, H. Akiyama, J. Caviness, H. A. Shill, D. J. Connor, M. N. Sabbagh, D. G. Walker, Arizona Parkinson’s Disease Consortium, Unified staging system for Lewy body disorders: Correlation with nigrostriatal degeneration, cognitive impairment and motor dysfunction. Acta Neuropathol. 117, 613–634 (2009). 19399512 10.1007/s00401-009-0538-8 PMC2757320

[10] L. M. Jackson, B. K. Woodruff, C. Tremblay, H. A. Shill, T. G. Beach, G. E. Serrano, C. H. Adler, Parkinson’s disease associated with G2019S LRRK2 mutations without Lewy body pathology. Mov Disord Clin Pract 11, 874–878 (2024). 38757351 10.1002/mdc3.14068 PMC11233835

[11] N. M. Jensen, Z. Vitic, M. R. Antorini, T. B. Viftrup, L. Parkkinen, P. H. Jensen, Abundant non-inclusion α-synuclein pathology in Lewy body-negative LRRK2-mutant cases. Acta Neuropathol. 149, 41 (2025). 40314782 10.1007/s00401-025-02871-w PMC12048437

[12] H. Sekiya, L. Franke, Y. Hashimoto, M. Takata, K. Nishida, N. Futamura, K. Hasegawa, H. Kowa, O. A. Ross, P. J. McLean, T. Toda, Z. K. Wszolek, D. W. Dickson, Widespread distribution of α-synuclein oligomers in LRRK2-related Parkinson’s disease. Acta Neuropathol. 149, 42 (2025). 40314842 10.1007/s00401-025-02872-9 PMC12395563

[13] M. B. Fares, S. Jagannath, H. A. Lashuel, Reverse engineering Lewy bodies: How far have we come and how far can we go? Nat. Rev. Neurosci. 22, 111–131 (2021). 33432241 10.1038/s41583-020-00416-6

[14] E. Kuusisto, L. Parkkinen, I. Alafuzoff, Morphogenesis of Lewy bodies: Dissimilar incorporation of alpha-synuclein, ubiquitin, and p62. J. Neuropathol. Exp. Neurol. 62, 1241–1253 (2003). 14692700 10.1093/jnen/62.12.1241

[15] A. J. Lewis, L. van den Heuve, M. D. Fabrizio, K. Bandelier, D. Proniakova, D. Burger, N. Shafiei, S. Ekundayo, S. Offringa, E. Huisman, J. G. J. M. Bol, W. D. J. van de Berg, H. Stahlberg, Ultrastructural diversity and subcellular organization of nigral Lewy pathology in Parkinson’s disease. Nat. Commun. 10, 10.1038/s41596-025-01153-9 (2026). 10.1038/s41467-026-74083-z PMC13396239 42259820

[16] S. E. Mastenbroek, J. W. Vogel, L. E. Collij, G. E. Serrano, C. Tremblay, A. L. Young, R. A. Arce, H. A. Shill, E. D. Driver-Dunckley, S. H. Mehta, C. M. Belden, A. Atri, P. Choudhury, F. Barkhof, C. H. Adler, R. Ossenkoppele, T. G. Beach, O. Hansson, Disease progression modelling reveals heterogeneity in trajectories of Lewy-type α-synuclein pathology. Nat. Commun. 15, 5133 (2024). 38879548 10.1038/s41467-024-49402-x PMC11180185

[17] S. H. Shahmoradian, A. J. Lewis, C. Genoud, J. Hench, T. E. Moors, P. P. Navarro, D. Castano-Diez, G. Schweighauser, A. Graff-Meyer, K. N. Goldie, R. Sutterlin, E. Huisman, A. Ingrassia, Y. Gier, A. J. M. Rozemuller, J. Wang, A. Paepe, J. Erny, A. Staempfli, J. Hoernschemeyer, F. Grosseruschkamp, D. Niedieker, S. F. El-Mashtoly, M. Quadri, I. W. F. J. Van, V. Bonifati, K. Gerwert, B. Bohrmann, S. Frank, M. Britschgi, H. Stahlberg, W. D. J. Van de Berg, M. E. Lauer, Lewy pathology in Parkinson’s disease consists of crowded organelles and lipid membranes. Nat. Neurosci. 22, 1099–1109 (2019). 31235907 10.1038/s41593-019-0423-2

[18] M. F. Altay, S. T. Kumar, J. Burtscher, S. Jagannath, C. Strand, Y. Miki, L. Parkkinen, J. L. Holton, H. A. Lashuel, Development and validation of an expanded antibody toolset that captures alpha-synuclein pathological diversity in Lewy body diseases. NPJ Parkinsons Dis. 9, 161 (2023). 38062007 10.1038/s41531-023-00604-y PMC10703845

[19] M. F. Altay, A. K. L. Liu, J. L. Holton, L. Parkkinen, H. A. Lashuel, Prominent astrocytic alpha-synuclein pathology with unique post-translational modification signatures unveiled across Lewy body disorders. Acta Neuropathol. Commun. 10, 163 (2022). 36371251 10.1186/s40478-022-01468-8 PMC9652889

[20] Z. A. Sorrentino, M. S. Goodwin, C. J. Riffe, J. S. Dhillon, Y. Xia, K. M. Gorion, N. Vijayaraghavan, K. N. McFarland, L. I. Golbe, A. T. Yachnis, B. I. Giasson, Unique α-synuclein pathology within the amygdala in Lewy body dementia: Implications for disease initiation and progression. Acta Neuropathol. Commun. 7, 142 (2019). 31477175 10.1186/s40478-019-0787-2 PMC6718048

[21] A. Becerra-Calixto, A. Mukherjee, S. Ramirez, S. Sepulveda, T. Sinha, R. Al-Lahham, N. De Gregorio, C. Gherardelli, C. Soto, Lewy body-like pathology and loss of dopaminergic neurons in midbrain organoids derived from familial Parkinson’s disease patient. Cells 12, 625 (2023). 36831291 10.3390/cells12040625 PMC9954141

[22] G. Bieri, M. Brahic, L. Bousset, J. Couthouis, N. J. Kramer, R. Ma, L. Nakayama, M. Monbureau, E. Defensor, B. Schüle, M. Shamloo, R. Melki, A. D. Gitler, LRRK2 modifies α-syn pathology and spread in mouse models and human neurons. Acta Neuropathol. 137, 961–980 (2019). 30927072 10.1007/s00401-019-01995-0 PMC6531417

[23] K. Burbidge, D. J. Rademacher, J. Mattick, S. Zack, A. Grillini, L. Bousset, O. Kwon, K. Kubicki, A. Simon, R. Melki, E. M. Campbell, LGALS3 (galectin 3) mediates an unconventional secretion of SNCA/α-synuclein in response to lysosomal membrane damage by the autophagic-lysosomal pathway in human midbrain dopamine neurons. Autophagy 18, 1020–1048 (2022). 34612142 10.1080/15548627.2021.1967615 PMC9196737

[24] M. C. Caiazza, C. Lang, R. Wade-Martins, What we can learn from iPSC-derived cellular models of Parkinson’s disease. Prog. Brain Res. 252, 3–25 (2020). 32247368 10.1016/bs.pbr.2019.11.002

[25] Y. Chen, K. S. Dolt, M. Kriek, T. Baker, P. Downey, N. J. Drummond, M. A. Canham, A. Natalwala, S. Rosser, T. Kunath, Engineering synucleinopathy-resistant human dopaminergic neurons by CRISPR-mediated deletion of the *SNCA* gene. Eur. J. Neurosci. 49, 510–524 (2019). 30472757 10.1111/ejn.14286 PMC6492083

[26] A. de Rus Jacquet, H. L. Denis, F. Cicchetti, M. Alpaugh, Current and future applications of induced pluripotent stem cell-based models to study pathological proteins in neurodegenerative disorders. Mol. Psychiatry 26, 2685–2706 (2021). 33495544 10.1038/s41380-020-00999-7 PMC8505258

[27] X. Diao, F. Wang, A. Becerra-Calixto, C. Soto, A. Mukherjee, Induced pluripotent stem cell-derived dopaminergic neurons from familial Parkinson’s disease patients display α-synuclein pathology and abnormal mitochondrial morphology. Cells 10, 2402 (2021). 34572052 10.3390/cells10092402 PMC8467069

[28] J. Gao, G. Perera, M. Bhadbhade, G. M. Halliday, N. Dzamko, Autophagy activation promotes clearance of α-synuclein inclusions in fibril-seeded human neural cells. J. Biol. Chem. 294, 14241–14256 (2019). 31375560 10.1074/jbc.RA119.008733 PMC6768637

[29] S. Gribaudo, P. Tixador, L. Bousset, A. Fenyi, P. Lino, R. Melki, J. M. Peyrin, A. L. Perrier, Propagation of α-synuclein strains within human reconstructed neuronal network. Stem Cell Rep. 12, 230–244 (2019). 10.1016/j.stemcr.2018.12.007 PMC6372945 30639210

[30] A. Iannielli, M. Luoni, S. G. Giannelli, R. Ferese, G. Ordazzo, M. Fossati, A. Raimondi, F. Opazo, O. Corti, J. H. M. Prehn, S. Gambardella, R. Melki, V. Broccoli, Modeling native and seeded Synuclein aggregation and related cellular dysfunctions in dopaminergic neurons derived by a new set of isogenic iPSC lines with SNCA multiplications. Cell Death Dis. 13, 881 (2022). 36261424 10.1038/s41419-022-05330-6 PMC9581971

[31] M. S. Kim, E. A. Ra, S. H. Kweon, B. A. Seo, H. S. Ko, Y. Oh, G. Lee, Advanced human iPSC-based preclinical model for Parkinson’s disease with optogenetic alpha-synuclein aggregation. Cell Stem Cell 30, 973–986.e11 (2023). 37339636 10.1016/j.stem.2023.05.015 PMC10829432

[32] I. Lam, A. Ndayisaba, A. J. Lewis, Y. Fu, G. T. Sagredo, A. Kuzkina, L. Zaccagnini, M. Celikag, J. Sandoe, R. L. Sanz, A. Vahdatshoar, T. D. Martin, N. Morshed, T. Ichihashi, A. Tripathi, N. Ramalingam, C. Oettgen-Suazo, T. Bartels, M. Boussouf, M. Schäbinger, E. Hallacli, X. Jiang, A. Verma, C. Tea, Z. Wang, H. Hakozaki, X. Yu, K. Hyles, C. Park, X. Wang, T. W. Theunissen, H. Wang, R. Jaenisch, S. Lindquist, B. Stevens, N. Stefanova, G. Wenning, W. D. J. van de Berg, K. C. Luk, R. Sanchez-Pernaute, J. C. Gómez-Esteban, D. Felsky, Y. Kiyota, N. Sahni, S. S. Yi, C. Y. Chung, H. Stahlberg, I. Ferrer, J. Schöneberg, S. J. Elledge, U. Dettmer, G. M. Halliday, T. Bartels, V. Khurana, Rapid iPSC inclusionopathy models shed light on formation, consequence, and molecular subtype of α-synuclein inclusions. Neuron 112, 2886–2909.e16 (2024). 39079530 10.1016/j.neuron.2024.06.002 PMC11377155

[33] D. Little, C. Luft, O. Mosaku, M. Lorvellec, Z. Yao, S. Paillusson, J. Kriston-Vizi, S. Gandhi, A. Y. Abramov, R. Ketteler, M. J. Devine, P. Gissen, A single cell high content assay detects mitochondrial dysfunction in iPSC-derived neurons with mutations in SNCA. Sci. Rep. 8, 9033 (2018). 29899557 10.1038/s41598-018-27058-0 PMC5998042

[34] A. L. Mahul-Mellier, M. F. Altay, N. Maharjan, N. Ait-Bouziad, A. Chiki, S. Jagannath, G. Limorenko, S. Novello, J. Ricci, Y. Jasiqi, S. Vingill, R. Wade-Martins, J. Holton, C. Strand, C. Haikal, J. Y. Li, R. Hamelin, M. Croisier, G. Knott, G. Mairet-Coello, L. Weerens, A. Michel, P. Downey, M. Citron, H. A. Lashuel, Differential role of C-terminal truncations on alpha-synuclein pathology and Lewy body formation. NPJ Parkinsons Dis. 11, 261 (2025). 40858624 10.1038/s41531-025-01084-y PMC12381302

[35] I. Pediaditakis, K. R. Kodella, D. V. Manatakis, C. Y. Le, C. D. Hinojosa, W. Tien-Street, E. S. Manolakos, K. Vekrellis, G. A. Hamilton, L. Ewart, L. L. Rubin, K. Karalis, Modeling alpha-synuclein pathology in a human brain-chip to assess blood-brain barrier disruption. Nat. Commun. 12, 5907 (2021). 34625559 10.1038/s41467-021-26066-5 PMC8501050

[36] P. Hornauer, G. Prack, N. Anastasi, S. Ronchi, T. Kim, C. Donner, M. Fiscella, K. Borgwardt, V. Taylor, R. Jagasia, D. Roqueiro, A. Hierlemann, M. Schröter, Downregulating α-synuclein in iPSC-derived dopaminergic neurons mimics electrophysiological phenotype of the A53T mutation. bioRxiv 10.1101/2022.03.31.486582 [Preprint] (2022); 10.1101/2022.03.31.486582.

[37] A. Spathopoulou, F. Edenhofer, L. Fellner, Targeting α-synuclein in Parkinson’s disease by induced pluripotent stem cell models. Front. Neurol. 12, 786835 (2022). 35145469 10.3389/fneur.2021.786835 PMC8821105

[38] H. Suzuki, N. Egawa, T. Kondo, K. Imamura, T. Enami, K. Tsukita, M. Suga, R. Shibukawa, Y. Okanishi, T. Uchiyama, H. Inoue, R. Takahashi, Generation of a human induced pluripotent stem cell line derived from a Parkinson’s disease patient carrying *SNCA* duplication. Stem Cell Res. 45, 101828 (2020). 32413791 10.1016/j.scr.2020.101828

[39] B. Tanudjojo, S. S. Shaikh, A. Fenyi, L. Bousset, D. Agarwal, J. Marsh, C. Zois, S. Heman-Ackah, R. Fischer, D. Sims, R. Melki, G. K. Tofaris, Phenotypic manifestation of α-synuclein strains derived from Parkinson’s disease and multiple system atrophy in human dopaminergic neurons. Nat. Commun. 12, 3817 (2021). 34155194 10.1038/s41467-021-23682-z PMC8217249

[40] C. Vajhøj, B. Schmid, A. Alik, R. Melki, K. Fog, B. Holst, T. C. Stummann, Establishment of a human induced pluripotent stem cell neuronal model for identification of modulators of A53T α-synuclein levels and aggregation. PLOS ONE 16, e0261536 (2021). 34932569 10.1371/journal.pone.0261536 PMC8691628

[41] V. D. Valderhaug, K. Heiney, O. H. Ramstad, G. Bråthen, W. L. Kuan, S. Nichele, A. Sandvig, I. Sandvig, Early functional changes associated with alpha-synuclein proteinopathy in engineered human neural networks. Am. J. Physiol. Cell Physiol. 320, C1141–C1152 (2021). 33950697 10.1152/ajpcell.00413.2020

[42] G. S. Virdi, M. L. Choi, J. R. Evans, Z. Yao, D. Athauda, S. Strohbuecker, R. S. Nirujogi, A. I. Wernick, N. Pelegrina-Hidalgo, C. Leighton, R. S. Saleeb, O. Kopach, H. Alrashidi, D. Melandri, J. Perez-Lloret, P. R. Angelova, S. Sylantyev, S. Eaton, S. Heales, D. A. Rusakov, D. R. Alessi, T. Kunath, M. H. Horrocks, A. Y. Abramov, R. Patani, S. Gandhi, Protein aggregation and calcium dysregulation are hallmarks of familial Parkinson’s disease in midbrain dopaminergic neurons. NPJ Parkinsons Dis. 8, 162 (2022). 36424392 10.1038/s41531-022-00423-7 PMC9691718

[43] F. Zambon, M. Cherubini, H. J. R. Fernandes, C. Lang, B. J. Ryan, V. Volpato, N. Bengoa-Vergniory, S. Vingill, M. Attar, H. D. E. Booth, W. Haenseler, J. Vowles, R. Bowden, C. Webber, S. A. Cowley, R. Wade-Martins, Cellular α-synuclein pathology is associated with bioenergetic dysfunction in Parkinson’s iPSC-derived dopamine neurons. Hum. Mol. Genet. 28, 2001–2013 (2019). 30753527 10.1093/hmg/ddz038 PMC6548224

[44] A. Bayati, R. Ayoubi, A. Aguila, C. E. Zorca, G. Deyab, C. Han, S. J. Recinto, E. Nguyen-Renou, C. Rocha, G. Maussion, W. Luo, I. Shlaifer, E. Banks, I. McDowell, E. Del Cid Pellitero, X. E. Ding, B. Sharif, P. Séguéla, M. Yaqubi, C. X. Chen, Z. You, N. Abdian, H. M. McBride, E. A. Fon, J. A. Stratton, T. M. Durcan, P. C. Nahirney, P. S. McPherson, Modeling Parkinson’s disease pathology in human dopaminergic neurons by sequential exposure to α-synuclein fibrils and proinflammatory cytokines. Nat. Neurosci. 27, 2401–2416 (2024). 39379564 10.1038/s41593-024-01775-4

[45] J. E. Beevers, M. C. Lai, E. Collins, H. D. E. Booth, F. Zambon, L. Parkkinen, J. Vowles, S. A. Cowley, R. Wade-Martins, T. M. Caffrey, *MAPT* genetic variation and neuronal maturity alter isoform expression affecting axonal transport in iPSC-derived dopamine neurons. Stem Cell Reports 9, 587–599 (2017). 28689993 10.1016/j.stemcr.2017.06.005 PMC5549835

[46] H. J. R. Fernandes, N. Patikas, S. Foskolou, S. F. Field, J. E. Park, M. L. Byrne, A. R. Bassett, E. Metzakopian, Single-cell transcriptomics of Parkinson’s disease human in vitro models reveals dopamine neuron-specific stress responses. Cell Rep. 33, 108263 (2020). 33053338 10.1016/j.celrep.2020.108263

[47] C. Lang, K. R. Campbell, B. J. Ryan, P. Carling, M. Attar, J. Vowles, O. V. Perestenko, R. Bowden, F. Baig, M. Kasten, M. T. Hu, S. A. Cowley, C. Webber, R. Wade-Martins, Single-cell sequencing of iPSC-dopamine neurons reconstructs disease progression and identifies HDAC4 as a regulator of Parkinson cell phenotypes. Cell Stem Cell 24, 93–106.e6 (2019). 30503143 10.1016/j.stem.2018.10.023 PMC6327112

[48] M. Sundberg, H. Bogetofte, T. Lawson, J. Jansson, G. Smith, A. Astradsson, M. Moore, T. Osborn, O. Cooper, R. Spealman, P. Hallett, O. Isacson, Improved cell therapy protocols for Parkinson’s disease based on differentiation efficiency and safety of hESC-, hiPSC-, and non-human primate iPSC-derived dopaminergic neurons. Stem Cells 31, 1548–1562 (2013). 23666606 10.1002/stem.1415 PMC3775937

[49] R. Sheta, M. Teixeira, W. Idi, A. Oueslati, Optimized protocol for the generation of functional human induced-pluripotent-stem-cell-derived dopaminergic neurons. STAR Protoc. 4, 102486 (2023). 37515763 10.1016/j.xpro.2023.102486 PMC10400954

[50] R. Sheta, M. Teixeira, W. Idi, M. Pierre, A. de Rus Jacquet, V. Emond, C. E. Zorca, B. Vanderperre, T. M. Durcan, E. A. Fon, F. Calon, M. Chahine, A. Oueslati, Combining NGN2 programming and dopaminergic patterning for a rapid and efficient generation of hiPSC-derived midbrain neurons. Sci. Rep. 12, 17176 (2022). 36229560 10.1038/s41598-022-22158-4 PMC9562300

[51] S. T. Kumar, A. L. Mahul-Mellier, R. N. Hegde, G. Rivière, R. Moons, A. Ibáñez de Opakua, P. Magalhães, I. Rostami, S. Donzelli, F. Sobott, M. Zweckstetter, H. A. Lashuel, A NAC domain mutation (E83Q) unlocks the pathogenicity of human alpha-synuclein and recapitulates its pathological diversity. Sci. Adv. 8, eabn0044 (2022). 35486726 10.1126/sciadv.abn0044 PMC9054026

[52] A. T. Balana, A. L. Mahul-Mellier, B. A. Nguyen, M. Horvath, A. Javed, E. R. Hard, Y. Jasiqi, P. Singh, S. Afrin, R. Pedretti, V. Singh, V. M. Lee, K. C. Luk, L. Saelices, H. A. Lashuel, M. R. Pratt, O-GlcNAc forces an α-synuclein amyloid strain with notably diminished seeding and pathology. Nat. Chem. Biol. 20, 646–655 (2024). 38347213 10.1038/s41589-024-01551-2 PMC11062923

[53] A. L. Mahul-Mellier, J. Burtscher, N. Maharjan, L. Weerens, M. Croisier, F. Kuttler, M. Leleu, G. W. Knott, H. A. Lashuel, The process of Lewy body formation, rather than simply α-synuclein fibrillization, is one of the major drivers of neurodegeneration. Proc. Natl. Acad. Sci. U.S.A. 117, 4971–4982 (2020). 32075919 10.1073/pnas.1913904117 PMC7060668

[54] L. A. Volpicelli-Daley, K. C. Luk, T. P. Patel, S. A. Tanik, D. M. Riddle, A. Stieber, D. F. Meaney, J. Q. Trojanowski, V. M. Lee, Exogenous alpha-synuclein fibrils induce Lewy body pathology leading to synaptic dysfunction and neuron death. Neuron 72, 57–71 (2011). 21982369 10.1016/j.neuron.2011.08.033 PMC3204802

[55] K. C. Luk, C. Song, P. O’Brien, A. Stieber, J. R. Branch, K. R. Brunden, J. Q. Trojanowski, V. M. Lee, Exogenous alpha-synuclein fibrils seed the formation of Lewy body-like intracellular inclusions in cultured cells. Proc. Natl. Acad. Sci. U.S.A. 106, 20051–20056 (2009). 19892735 10.1073/pnas.0908005106 PMC2785290

[56] A. Bayati, E. Banks, C. Han, W. Luo, W. E. Reintsch, C. E. Zorca, I. Shlaifer, E. Del Cid Pellitero, B. Vanderperre, H. M. McBride, E. A. Fon, T. M. Durcan, P. S. McPherson, Rapid macropinocytic transfer of α-synuclein to lysosomes. Cell Rep. 40, 111102 (2022). 35858558 10.1016/j.celrep.2022.111102

[57] S. A. Tanik, C. E. Schultheiss, L. A. Volpicelli-Daley, K. R. Brunden, V. M. Lee, Lewy body-like alpha-synuclein aggregates resist degradation and impair macroautophagy. J. Biol. Chem. 288, 15194–15210 (2013). 23532841 10.1074/jbc.M113.457408 PMC3663539

[58] A. Sanyal, G. Scanavachi, E. Somerville, A. Saminathan, A. Nair, R. F. B. D. C. Correia, B. Aylan, E. Sitarska, A. Oikonomou, N. S. Hatzakis, T. Kirchhausen, Neuronal constitutive endolysosomal perforations enable α-synuclein aggregation by internalized PFFs. J. Cell Biol. 224, e202401136 (2025). 39714357 10.1083/jcb.202401136 PMC11665449

[59] M. E. Diaz-Ortiz, Y. Seo, M. Posavi, M. Carceles Cordon, E. Clark, N. Jain, R. Charan, M. D. Gallagher, T. L. Unger, N. Amari, R. T. Skrinak, R. Davila-Rivera, E. M. Brody, N. Han, R. Zack, V. M. Van Deerlin, T. F. Tropea, K. C. Luk, E. B. Lee, D. Weintraub, A. S. Chen-Plotkin, GPNMB confers risk for Parkinson’s disease through interaction with α-synuclein. Science 377, eabk0637 (2022). 35981040 10.1126/science.abk0637 PMC9870036

[60] C. Paschou, O. Apokotou, A. Kollias, K. Charmpi, S. Dede, M. Samiotaki, F. Palese, K. Dimoula, E. Emmanouilidou, E. Taoufik, C. Zurzolo, R. Matsas, F. Papastefanaki, Proteostasis dysregulation in p.A53T-α-Synuclein iPSC-derived astrocytes exacerbates neurodegeneration in a Parkinson’s disease model with Lewy-like pathology. bioRxiv 10.1101/2024.11.06.621638 [Preprint] (2025); 10.1101/2024.11.06.621638.

[61] R. Vroman, L. de Lichtervelde, K. Singh Dolt, G. Robertson, M. Kriek, M. Barbato, J. Cholewa-Waclaw, T. Kunath, P. Downey, M. Zagnoni, A high-fidelity microfluidic platform reveals retrograde propagation as the main mechanism of α-Synuclein spread in human neurons. NPJ Parkinsons Dis. 11, 80 (2025). 40254612 10.1038/s41531-025-00936-x PMC12009960

[62] F. F. Geibl, M. T. Henrich, Z. Xie, E. Zampese, J. Ueda, T. Tkatch, D. L. Wokosin, E. Nasiri, C. A. Grotmann, V. L. Dawson, T. M. Dawson, N. S. Chandel, W. H. Oertel, D. J. Surmeier, α-Synuclein pathology disrupts mitochondrial function in dopaminergic and cholinergic neurons at-risk in Parkinson’s disease. Mol. Neurodegener. 19, 69 (2024). 39379975 10.1186/s13024-024-00756-2 PMC11462807

[63] E. Hallacli, C. Kayatekin, S. Nazeen, X. H. Wang, Z. Sheinkopf, S. Sathyakumar, S. Sarkar, X. Jiang, X. Dong, R. Di Maio, W. Wang, M. T. Keeney, D. Felsky, J. Sandoe, A. Vahdatshoar, N. D. Udeshi, D. R. Mani, S. A. Carr, S. Lindquist, P. L. De Jager, D. P. Bartel, C. L. Myers, J. T. Greenamyre, M. B. Feany, S. R. Sunyaev, C. Y. Chung, V. Khurana, The Parkinson’s disease protein alpha-synuclein is a modulator of processing bodies and mRNA stability. Cell 185, 2035–2056.e33 (2022). 35688132 10.1016/j.cell.2022.05.008 PMC9394447

[64] A. Iwai, E. Masliah, M. Yoshimoto, N. Ge, L. Flanagan, H. A. de Silva, A. Kittel, T. Saitoh, The precursor protein of non-Aβ component of Alzheimer’s disease amyloid is a presynaptic protein of the central nervous system. Neuron 14, 467–475 (1995). 7857654 10.1016/0896-6273(95)90302-x

[65] B. N. Dugger, D. W. Dickson, Cell type specific sequestration of choline acetyltransferase and tyrosine hydroxylase within Lewy bodies. Acta Neuropathol. 120, 633–639 (2010). 20721565 10.1007/s00401-010-0739-1 PMC3107979

[66] S. Lehrer, P. H. Rheinstein, α-synuclein enfolds tyrosine hydroxylase and dopamine ß-hydroxylase, potentially reducing dopamine and norepinephrine synthesis. J. Proteins Proteom. 13, 109–115 (2022). 36277464 10.1007/s42485-022-00088-z PMC9585989

[67] A. Wawer, I. Joniec-Maciejak, A. Sznejder-Pachołek, J. Schwenkgrub, A. Ciesielska, D. Mirowska-Guzel, Exogenous α-synuclein monomers alter dopamine metabolism in murine brain. Neurochem. Res. 41, 2102–2109 (2016). 27161373 10.1007/s11064-016-1923-z

[68] F. De Giorgi, F. Laferrière, F. Zinghirino, E. Faggiani, A. Lends, M. Bertoni, X. Yu, A. Grélard, E. Morvan, B. Habenstein, N. Dutheil, E. Doudnikoff, J. Daniel, S. Claverol, C. Qin, A. Loquet, E. Bezard, F. Ichas, Novel self-replicating α-synuclein polymorphs that escape ThT monitoring can spontaneously emerge and acutely spread in neurons. Sci. Adv. 6, eabc4364 (2020). 33008896 10.1126/sciadv.abc4364 PMC7852382

[69] S. Donzelli, S. A. Osullivan, A.-L. Mahul-Mellier, A. Ulusoy, G. Fusco, S. T. Kumar, A. Chiki, J. Burtscher, M. L. D. Boussouf, I. Rostami, A. D. Simone, D. A. Di Monte, H. A. Lashuel, Post-fibrillization nitration of alpha-synuclein abolishes its seeding activity and pathology formation in primary neurons and *in vivo*. bioRxiv 10.1101/2023.03.24.534149 [Preprint] (2023); 10.1101/2023.03.24.534149.

[70] Y. Miki, K. Tanji, K. Shinnai, M. T. Tanaka, F. Altay, S. C. Foti, C. Strand, T. Sasaki, T. Kon, S. Shimoyama, T. Furukawa, H. Nishijima, H. Yamazaki, Y. T. Asi, C. Bettencourt, Z. Jaunmuktane, M. Tada, F. Mori, H. Mizukami, M. Tomiyama, H. A. Lashuel, T. Lashley, A. Kakita, H. Ling, A. J. Lees, J. L. Holton, T. T. Warner, K. Wakabayashi, Pathological substrate of memory impairment in multiple system atrophy. Neuropathol. Appl. Neurobiol. 48, e12844 (2022). 35906771 10.1111/nan.12844

[71] M. Hariri, G. Millane, M. P. Guimond, G. Guay, J. W. Dennis, I. R. Nabi, Biogenesis of multilamellar bodies via autophagy. Mol. Biol. Cell 11, 255–268 (2000). 10637306 10.1091/mbc.11.1.255 PMC14772

[72] J. P. Luzio, Y. Hackmann, N. M. Dieckmann, G. M. Griffiths, The biogenesis of lysosomes and lysosome-related organelles. Cold Spring Harb. Perspect. Biol. 6, a016840 (2014). 25183830 10.1101/cshperspect.a016840 PMC4142962

[73] M. A. G. Gilbert, N. Fatima, J. Jenkins, T. J. O’Sullivan, A. Schertel, Y. Halfon, M. Wilkinson, T. H. J. Morrema, M. Geibel, R. J. Read, N. A. Ranson, S. E. Radford, J. J. M. Hoozemans, R. A. W. Frank, CryoET of β-amyloid and tau within postmortem Alzheimer’s disease brain. Nature 631, 913–919 (2024). 38987603 10.1038/s41586-024-07680-x PMC11269202

[74] R. A. Nixon, J. Wegiel, A. Kumar, W. H. Yu, C. Peterhoff, A. Cataldo, A. M. Cuervo, Extensive involvement of autophagy in Alzheimer disease: An immuno-electron microscopy study. J. Neuropathol. Exp. Neurol. 64, 113–122 (2005). 15751225 10.1093/jnen/64.2.113

[75] L. S. Forno, R. L. Norville, Ultrastructure of Lewy bodies in the stellate ganglion. Acta Neuropathol. 34, 183–197 (1976). 178142 10.1007/BF00688674

[76] K. Wakabayashi, K. Tanji, S. Odagiri, Y. Miki, F. Mori, H. Takahashi, The Lewy body in Parkinson’s disease and related neurodegenerative disorders. Mol. Neurobiol. 47, 495–508 (2013). 22622968 10.1007/s12035-012-8280-y

[77] J. B. Leverenz, I. Umar, Q. Wang, T. J. Montine, P. J. McMillan, D. W. Tsuang, J. Jin, C. Pan, J. Shin, D. Zhu, J. Zhang, Proteomic identification of novel proteins in cortical Lewy bodies. Brain Pathol. 17, 139–145 (2007). 17388944 10.1111/j.1750-3639.2007.00048.x PMC8095629

[78] M. A. McFarland, C. E. Ellis, S. P. Markey, R. L. Nussbaum, Proteomics analysis identifies phosphorylation-dependent α-synuclein protein interactions. Mol. Cell. Proteomics 7, 2123–2137 (2008). 18614564 10.1074/mcp.M800116-MCP200 PMC2577212

[79] Q. Xia, L. Liao, D. Cheng, D. M. Duong, M. Gearing, J. J. Lah, A. I. Levey, J. Peng, Proteomic identification of novel proteins associated with Lewy bodies. Front. Biosci. 13, 3850–3856 (2008). 18508479 10.2741/2973 PMC2663966

[80] T. E. Moors, C. A. Maat, D. Niedieker, D. Mona, D. Petersen, E. Timmermans-Huisman, J. Kole, S. F. El-Mashtoly, L. Spycher, W. Zago, R. Barbour, O. Mundigl, K. Kaluza, S. Huber, M. N. Hug, T. Kremer, M. Ritter, S. Dziadek, J. J. G. Geurts, K. Gerwert, M. Britschgi, W. D. J. van de Berg, The subcellular arrangement of alpha-synuclein proteoforms in the Parkinson’s disease brain as revealed by multicolor STED microscopy. Acta Neuropathol. 142, 423–448 (2021). 34115198 10.1007/s00401-021-02329-9 PMC8357756

[81] C. Böing, M. Di Fabrizio, D. Burger, J. Bol, E. Huisman, A. J. M. Rozemuller, W. D. J. van de Berg, H. Stahlberg, A. J. Lewis, Distinct ultrastructural phenotypes of glial and neuronal alpha-synuclein inclusions in multiple system atrophy. Brain 147, 3727–3741 (2024). 38696728 10.1093/brain/awae137 PMC11531854

[82] N. Shafiei, D. Stähli, D. Burger, M. D. Fabrizio, L. van den Heuvel, J. Daraspe, C. Böing, S. H. Shahmoradian, W. D. J. van de Berg, C. Genoud, H. Stahlberg, A. J. Lewis, Correlative light and electron microscopy for human brain and other biological models. Nat. Protoc. 20, 2994–3023 (2025). 40164750 10.1038/s41596-025-01153-9

[83] L. V. Albrecht, N. Tejeda-Muñoz, E. M. De Robertis, Protocol for probing regulated lysosomal activity and function in living cells. STAR Protoc 1, 100132 (2020). 33377026 10.1016/j.xpro.2020.100132 PMC7757114

[84] A. Dilsizoglu Senol, M. Samarani, S. Syan, C. M. Guardia, T. Nonaka, N. Liv, P. Latour-Lambert, M. Hasegawa, J. Klumperman, J. S. Bonifacino, C. Zurzolo, α-Synuclein fibrils subvert lysosome structure and function for the propagation of protein misfolding between cells through tunneling nanotubes. PLOS Biol. 19, e3001287 (2021). 34283825 10.1371/journal.pbio.3001287 PMC8291706

[85] H. A. Lashuel, Do Lewy bodies contain alpha-synuclein fibrils? and Does it matter? A brief history and critical analysis of recent reports. Neurobiol. Dis. 141, 104876 (2020). 32339655 10.1016/j.nbd.2020.104876

[86] N. Riguet, A. L. Mahul-Mellier, N. Maharjan, J. Burtscher, M. Croisier, G. Knott, J. Hastings, A. Patin, V. Reiterer, H. Farhan, S. Nasarov, H. A. Lashuel, Nuclear and cytoplasmic huntingtin inclusions exhibit distinct biochemical composition, interactome and ultrastructural properties. Nat. Commun. 12, 6579 (2021). 34772920 10.1038/s41467-021-26684-z PMC8589980

[87] A. Sadek, B. E. Correia, H. A. Lashuel, Mapping the structural landscape of amyloid fibrils to guide polymorph-specific therapeutics. bioRxiv 10.1101/2025.05.08.652887 [Preprint] (2025); 10.1101/2025.05.08.652887.

[88] V. A. Trinkaus, I. Riera-Tur, A. Martínez-Sánchez, F. J. B. Bäuerlein, Q. Guo, T. Arzberger, W. Baumeister, I. Dudanova, M. S. Hipp, F. U. Hartl, R. Fernández-Busnadiego, In situ architecture of neuronal α-Synuclein inclusions. Nat. Commun. 12, 2110 (2021). 33854052 10.1038/s41467-021-22108-0 PMC8046968

[89] A. Bayati, P. S. McPherson, Alpha-synuclein, autophagy-lysosomal pathway, and Lewy bodies: Mutations, propagation, aggregation, and the formation of inclusions. J. Biol. Chem. 300, 107742 (2024). 39233232 10.1016/j.jbc.2024.107742 PMC11460475

[90] L. S. Forno, L. E. DeLanney, I. Irwin, J. W. Langston, Electron microscopy of Lewy bodies in the amygdala-parahippocampal region. Comparison with inclusion bodies in the MPTP-treated squirrel monkey. Adv. Neurol. 69, 217–228 (1996). 8615131

[91] P. E. Duffy, V. M. Tennyson, Phase and electron microscopic observations of Lewy bodies and melanin granules in the substantia nigra and locus caeruleus in Parkinson’s disease. J. Neuropathol. Exp. Neurol. 24, 398–414 (1965).

[92] M. Baba, S. Nakajo, P. H. Tu, T. Tomita, K. Nakaya, V. M. Lee, J. Q. Trojanowski, T. Iwatsubo, Aggregation of alpha-synuclein in Lewy bodies of sporadic Parkinson’s disease and dementia with Lewy bodies. Am. J. Pathol. 152, 879–884 (1998). 9546347 PMC1858234

[93] P. G. Galloway, P. Mulvihill, G. Perry, Filaments of Lewy bodies contain insoluble cytoskeletal elements. Am. J. Pathol. 140, 809–822 (1992). 1314025 PMC1886359

[94] S. Kuzuhara, H. Mori, N. Izumiyama, M. Yoshimura, Y. Ihara, Lewy bodies are ubiquitinated. A light and electron microscopic immunocytochemical study. Acta Neuropathol. 75, 345–353 (1988). 3364159 10.1007/BF00687787

[95] B. Sonustun, M. F. Altay, C. Strand, K. Ebanks, G. Hondhamuni, T. T. Warner, H. A. Lashuel, R. Bandopadhyay, Pathological relevance of post-translationally modified alpha-synuclein (pSer87, pSer129, nTyr39) in idiopathic Parkinson’s disease and multiple system atrophy. Cells 11, 906 (2022). 35269528 10.3390/cells11050906 PMC8909017

[96] W. A. den Jager, Sphingomyelin in Lewy inclusion bodies in Parkinson’s disease. Arch. Neurol. 21, 615–619 (1969). 4187718 10.1001/archneur.1969.00480180071006

[97] W. P. Gai, H. X. Yuan, X. Q. Li, J. T. Power, P. C. Blumbergs, P. H. Jensen, In situ and in vitro study of colocalization and segregation of alpha-synuclein, ubiquitin, and lipids in Lewy bodies. Exp. Neurol. 166, 324–333 (2000). 11085897 10.1006/exnr.2000.7527

