# Supplementary material for: Modeling Parkinson’s pathology in human iPSC dopaminergic neurons uncovers key mechanisms of Lewy body formation and heterogeneity
Source: Sci Adv. 2026 Jul 10;12(28):eaed8851. doi: 10.1126/sciadv.aed8851 (PMC13353426; doi:10.1126/sciadv.aed8851)
Supplement: Supplementary file 1 — Figs. S1 to S16 Legend for data S1 [file sciadv.aed8851_sm.pdf]

Science Advances
AAAS

Supplementary Materials for

Modeling Parkinson's pathology in human iPSC dopaminergic neurons uncovers key mechanisms of Lewy body formation and heterogeneity

Anne-Laure Mahul-Mellier *et al.*

Corresponding author: Anne-Laure Mahul-Mellier, anne-laure.mahul@epfl.ch;
Hilal A. Lashuel, hil4001@qatar-med.cornell.edu

Sci. Adv. **12**, eaed8851 (2026)
DOI: 10.1126/sciadv.aed8851

The PDF file includes:

Figs. S1 to S16
Legend for data S1

Other Supplementary Material for this manuscript includes the following:

Data S1

Supplementary materials

Supplemental information - Figures titles and Legends

Figure S1

A

Primary Antibody	Catalog #	Company	Clone	RRID	Host	Concentration	WB dilution	ICC dilution	Epitope
anti-aSyn total	610787	BD	SYN-1	AB_398108	Mouse	0.25 mg/mL	1:1000	1:1000	91-99
anti-aSyn total	807803	Biolegend	4B12	AB_2564731	Mouse	1 mg/mL	1:2000	1:1000	103-108
anti-aSyn total	ab131508	Abcam	-	AB_11155736	Rabbit	1 mg/mL	1:1000	1:500	134-138

B

Primary Antibody	Catalog #	Company	Clone	RRID	Host	Concentration	WB dilution	ICC dilution	Epitope
anti-**pS129**-aSyn	825701	BioLegend	p-syn/81A	AB_2564891	Mouse	1.0 mg/mL	1:1000	1:2000	Peptide (residues 124-134) including phosphorylated Ser129 of human aSyn
anti-**pS129**-aSyn	015-25191	Wako	pSyn #64	AB_2537218	Mouse	1.0 mg/mL	1:1000	1:2000	residues 124-134
anti-**pS129**-aSyn	ab168381	Abcam	MJF-R13	AB_2728613	Rabbit	4.229 mg/mL	1:3000	1:3000	The exact sequence is proprietary
anti-**pS129**-aSyn	ab51253	Abcam	EP1536Y	AB_869973	Rabbit	2.578 mg/mL	1:500	1:250	Synthetic peptide within human aSyn (aa 100 to the C-terminus). The exact sequence is proprietary.
anti-**pY39**-aSyn	849202	Biolegend	A15119B	AB_2650702	Mouse	0.5 mg/mL	1:1000	1:500	KLH-conjugated α-synuclein synthetic peptide containing pY39
anti-**pS87**-aSyn	Lashuel	-	128005	-	Rabbit	0.1 mg/mL	Not tested	1:500	H2N- C A Q K T V E G A G S(p) I A A -CONHH
anti-**pY125**-aSyn	ab10789	Abcam	phospho Y125	AB_442822	Rabbit	0.1 mg/mL	Not tested	1:500	100 to the C-terminus pY125 The exact sequence is proprietary
anti-**pY133**-aSyn	ab194910	Abcam	phospho Y133	Not available	Rabbit	1.5 mg/ml	Not tested	1:500	pY133
anti-**pY136**-aSyn	ab131491	Abcam	phospho Y136	AB_11159508	Rabbit	1 mg/mL	Not tested	1:500	phospho Y136 aa 100 to C-terminus. The exact sequence is proprietary.
anti-**Ni**-aSyn (total)	838903	Biolegend	Syn514	AB_2616740	Mouse	0.5 mg/mL	Not tested	1:500	Syn514 was raised using oxidized alpha-synuclein (α-synuclein) and recognizes its native and nitrated/oxidized forms
anti-**NiY39**-aSyn	36-012	Merck - Millipore	nSyn14	AB_310821	Mouse	0.5 mg/mL	Not tested	1:500	Full-length recombinant human α-synuclein nitrated in vitro.
anti-**NiY125-136** aSyn	36-011	Merck - Millipore	nSyn12	AB_310820	Mouse	0.5 mg/mL	Not tested	1:500	Full-length recombinant human α-synuclein nitrated in vitro.

C

Primary Antibody	Catalog #	Company	Clone	RRID	Host	Concentration	WB dilution	ICC dilution	
anti-**actin**	ab6276	Abcam	AC-15	AB_2223210	Mouse	2.2 mg/mL	1:5000	Not tested	DDDIAALVIDNGSGK
anti-**MAP2**	ab92434	Abcam	-	AB_2138147	Chicken	Not provided	Not tested	1:2000	Recombinant full length protein
anti-**p62**	H00008878	Abnova	2C11	AB_437085	Mouse	1 mg/mL	1:1000	1:500	
anti-**p62**	18420-1	Proteintech		AB_10694431	Rabbit	0.7 mg/mL	Not tested	1:500	NA
anti-**ubiquitin**	Sc-8017	Santa-Cruz	P4D1	AB_628423	Mouse	0.2 mg/mL	-	1:100	1-76
anti-**β-tubulin III**	AB9354	Merck		AB_570918	Chicken	1 mg/mL	1:2000	1:2000	
anti-**β-Tubulin III**	ab18207	Abcam	-	AB_444319	Rabbit	1 mg/mL	1:1000	1:500	Synthetic peptide corresponding to Human beta III Tubulin aa 350 to the C-terminus
anti-**TH**	ab76442	Abcam	-	AB_1524535	Chicken	0.2 mg/mL	1:1000	1:500	Synthetic Peptide within Human TH conjugated to Keyhole Limpet Haemocyanin.
anti-**TH**	AB152	Merck - Millipore	-	AB_390204	Rabbit	150 µg/mL	1:1000	1:500	Denatured tyrosine hydroxylase from rat pheochromocytoma
anti-**NFL**	837801	BioLegend	SMI 311	AB_2565384	Mouse	1 mg/mL	Not tested	1:500	-
anti-**NEFL**	13-0400	Thermo Fisher	DA2	AB_2532995	Mouse	0.5 mg/mL	Not tested	1:500	Specifically recognizes a phosphate-independent epitope on NF-L.
anti-**NF-H**	AB5539	Millipore	-	AB_11212161	Chicken	1 mg/mL	Not tested	1:500	
anti-**LAMP1**	ab24170	Abcam	-	AB_775978	Rabbit	?	Not tested	1:500	Synthetic peptide corresponding to Human LAMP1 aa 400 to the C-terminus
anti-**BIP/Grp78**	ab21685	Abcam	-	AB_2119834	Rabbit	1 mg/mL	1:1000	1:500	Synthetic peptide conjugated to KLH derived from within residues 600 to the C-terminus
anti-**Tom20**	ab186735	Abcam	-	AB_2889972	Rabbit	1 mg/mL	Not tested	1:500	The exact sequence is proprietary
**anti-GM130**	610823	BD	Clone 35	AB_398142	Mouse	250 mg/mL	Not tested	1:250	surrounding Thr185

D

Secondary Antibody	Catalog #	Company	RRID	Concentration	WB dilution	ICC dilution
Goat anti-**mouse Alexa Fluor 680**	A21058	Invitrogen	AB_2535724	2 mg/mL	1:20'000	-
Goat anti-**rabbit Alexa Fluor 680**	A21109	Invitrogen	AB_2535758	2 mg/mL	1:20'000	-
Goat anti-**mouse Alexa Fluor 800**	926-32210	Li-Cor	AB_621842	1 mg/mL	1:20'000	-
Goat anti-**rabbit Alexa Fluor 800**	926-32211	Li-Cor	AB_621843	1 mg/mL	1:20'000	-
Donkey anti-**rabbit Alexa Fluor 647**	A31573	Invitrogen	AB_2536183	2 mg/mL	-	1:800
Donkey anti-**mouse Alexa Fluor 647**	A31571	Invitrogen	AB_162542	2 mg/mL	-	1:800
Goat anti-**chicken Alexa Fluor 568**	A11041	Invitrogen	AB_2534098	2 mg/mL	-	1:500
Goat anti-**mouse Alexa Fluor 488**	A-11029	Invitrogen	AB_2534088	2 mg/mL	-	1:800
Donkey anti-**chicken Alexa Fluor 488**	703-545-155	Jackson Immunoresearch	AB_2340375	1 mg/mL	-	1:400
Donkey anti-**rabbit Alexa Fluor 488**	A21206	Invitrogen	AB_2535792	2 mg/mL	-	1:800

Figure S1. List of the antibodies used in this study
(**A**) Antibodies used for the detection of total aSyn.
(**B**) Antibodies used for the detection of aSyn PTM.
(**C**) Other antibodies used in the study.
(**D**) Secondary antibodies used for immunoblotting or confocal imaging.

Supplementary materials

Figure S2

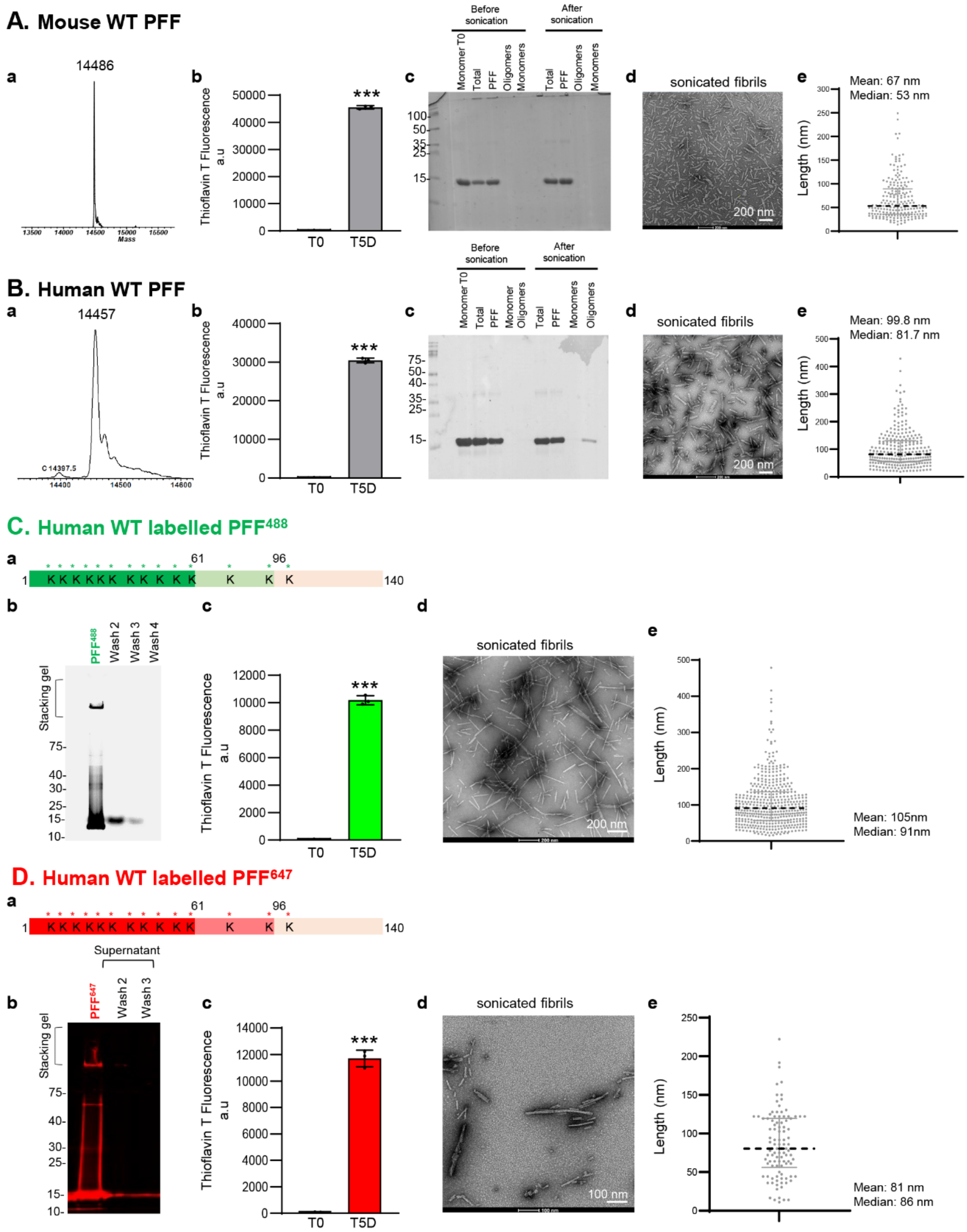

Figure S2. Preparation and characterization of recombinant monomeric and PFF aSyn species.
(**A-D**) Mouse aSyn (**A**) and human (**B-D**) aSyn wild-type (WT) were produced in *E. coli* and purified by anion exchange chromatography and size-exclusion chromatography, followed by a final chromatographic step using reverse-phase HPLC, as previously described *(52, 54)*. **a.** Purity and characterization of aSyn monomers. The purity of recombinant monomeric aSyn after purification was assessed by ESI-LC/MS, which showed the expected mass. **b-e.** Purity and characterization of aSyn PFF. aSyn PFF were formed by incubation of monomeric aSyn for 5 days (T5D) at 37°C under constant agitation at 1000 rpm. After fibrilization, part of human aSyn PFF were labelled with Atto488 maleimide (**C**) or Atto647 (**D**). **b**. After sonication, PFF formation was assessed by ThT fluorometry. All data represent the average ± SD (n=3). **c**. The purity of aSyn PFF was verified by SDS-PAGE gel and Coomassie blue staining. After sonication, PFF preparations were centrifuged, and the presence of the PFF was verified in the pellet fraction, while the absence of monomer release after the sonication step was assessed in the supernatant fraction or after filtration through a 100 kDa filter (filtration). **d-e**. aSyn PFF were characterized using transmission electron microscopy (TEM). (**d**) Representative images of negatively stained aSyn PFF before and after sonication. All aSyn PFF showed the characteristic rigid non-branched fibrillar morphology. Scale bars = 200 nm (**Ad** and **Bd**) and 100 nm (**Dd**). (**E**) The average length of the PFF after sonication. (**Aa, Bb, Cc, Dc**) Data represent mean ± standard deviation (SD) from a minimum of three technical replicates (N=3). Statistical analyses: ANOVA with Tukey HSD post hoc tests. ***p<0.0001 (T0 vs. T5D).

Supplementary materials

Figure S3

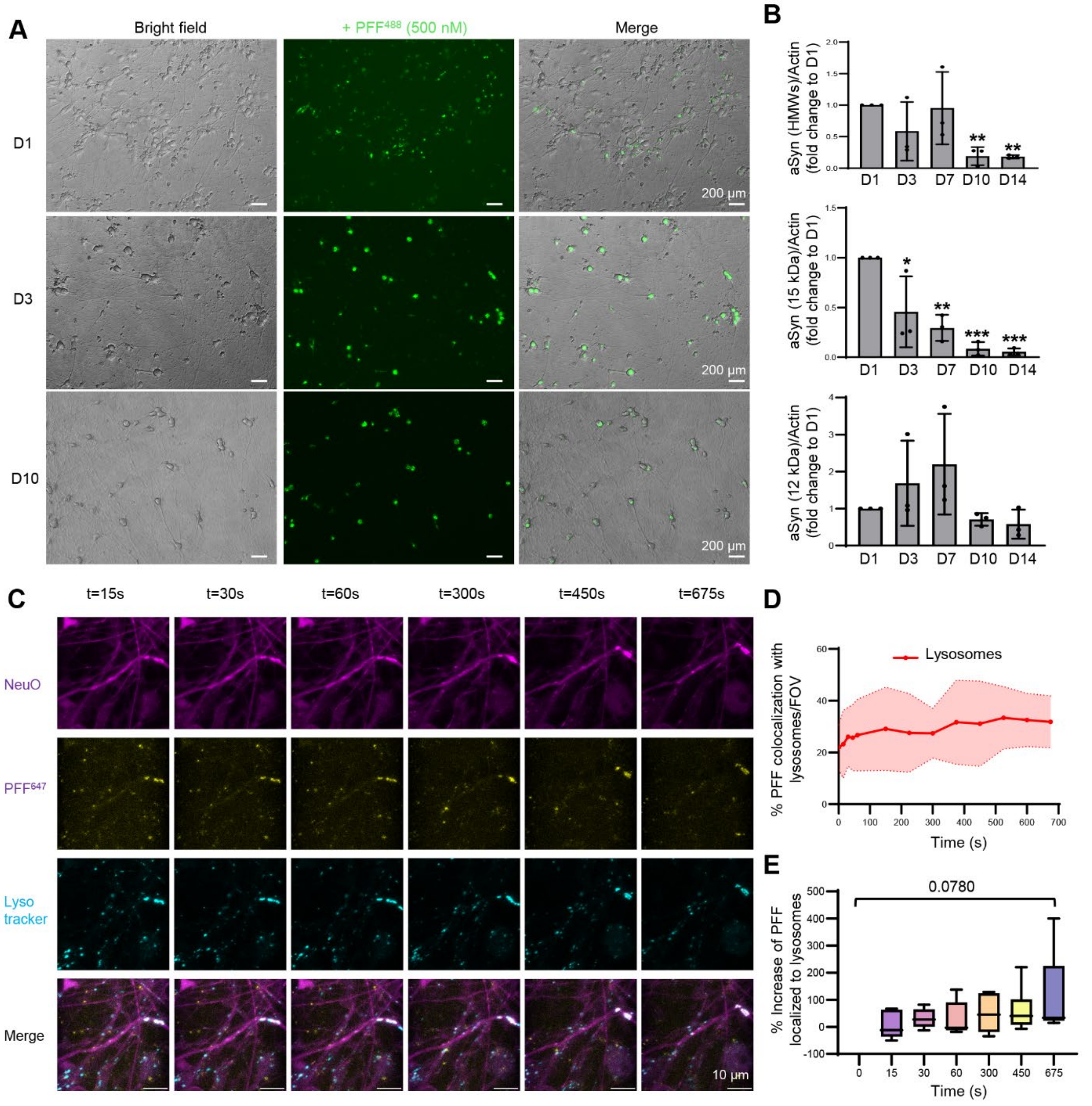

Figure S3. Internalisation, processing, and clearance of aSyn PFF seeds over time in human iPSC-derived dopaminergic neurons.

(**A**) Individual channels of the images showing fluorescently labelled PFF (PFF^{488}) internalised by iDA neurons at D1, D4, and D10 post-treatment in **Figure 1E**. Scale bars = 200 µm. (**B**) WB analyses of immunoblots shown in **Figure 1G**. Levels of full-length aSyn (15 kDa) or truncated aSyn (12 kDa) or HMWs were quantified by densitometry and normalized to actin (see immunoblots in Figure 1G). (**C**) Representative live-cell confocal images showing iDA (NeuO, purple) at DIV10 exposed to labelled PFF^{647} (yellow) for 20 min and showing colocalization in real time with active lysosomes (lysotracker, cyan) right after the PFF settles. (**D**) Graphical representation of the colocalization between PFFs and lysosomes over time, showing a stable interaction between both entities (20 to 30% colocalizing). (**E**) Box plots show the increase in colocalization signal between Lysotracker and PFF^{647} over time. (**B, D-E**) Data represent mean ± SD from a minimum of three independent biological replicates (**B**. N=3; **D.** N=8, and **E.** N=6). Statistical analyses: ANOVA with Tukey HSD post hoc tests. (**B**) *$p<0.01$, **$p<0.001$, ***$p<0.0001$ (D1 vs. others time-points). (**D-E**) No statistically significant differences.

Supplementary materials

Figure S4

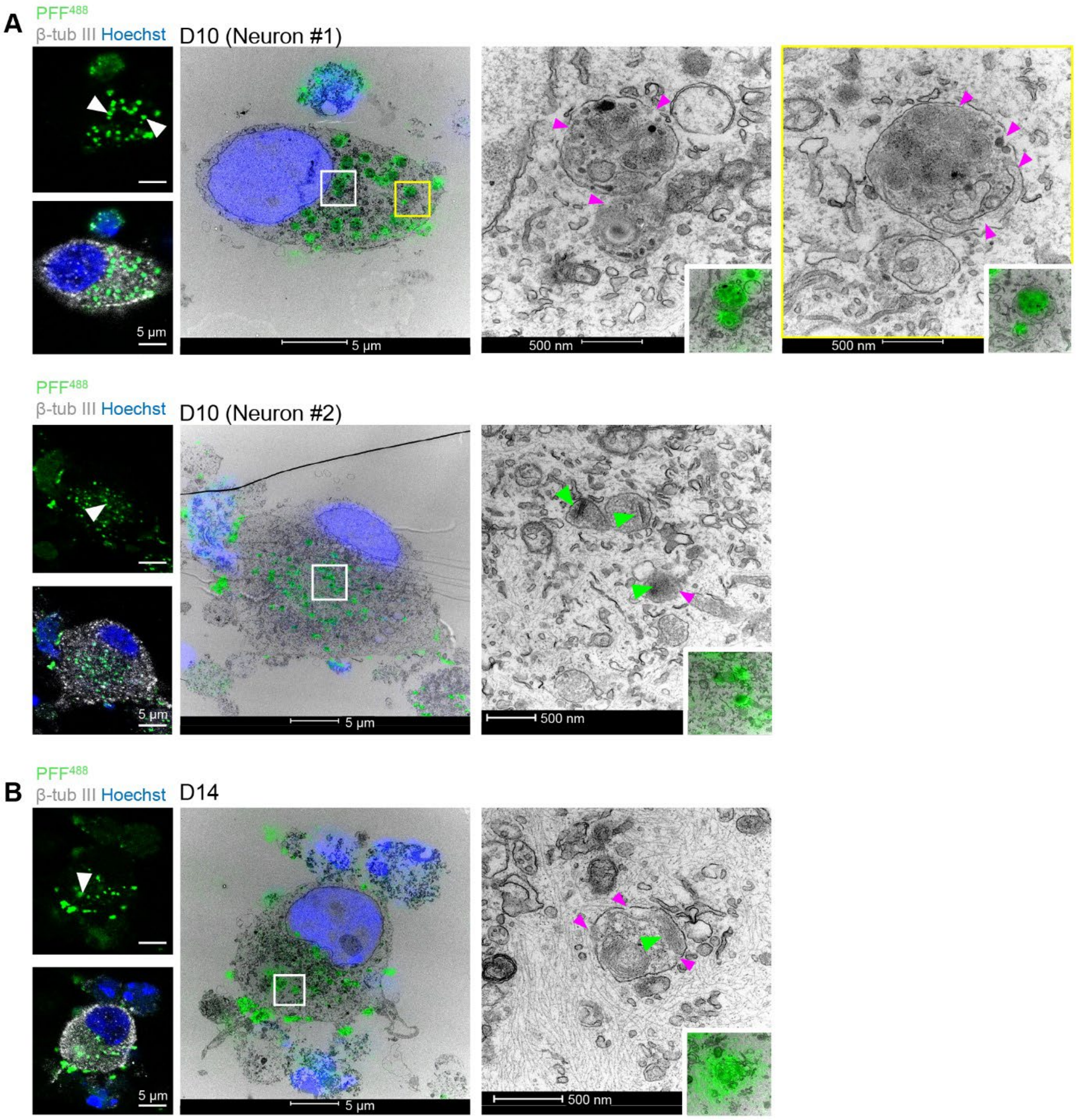

Figure S4. Correlative light and electron microscopy of endolysosomal structures in neurons challenged with fluorescently labelled PFF (PFF^{488}).
(**A**) Ten days after fibril addition (D10), PFF^{488} accumulate in large (>500 nm), membrane-bound endolysosomal structures containing membranes and other subcompartments. Rupture of the endolysosomal membrane is indicated by the pink arrow in Neuron #1. In another example (Neuron #2), PFF^{488}-positive structures contained laterally associated fibrils (green arrows). (**B**) A late endolysosome with a ruptured membrane (pink arrows) containing membranes and fibrils (green arrow). Scale bars = 5 µm and 500 nm.

Figure S5

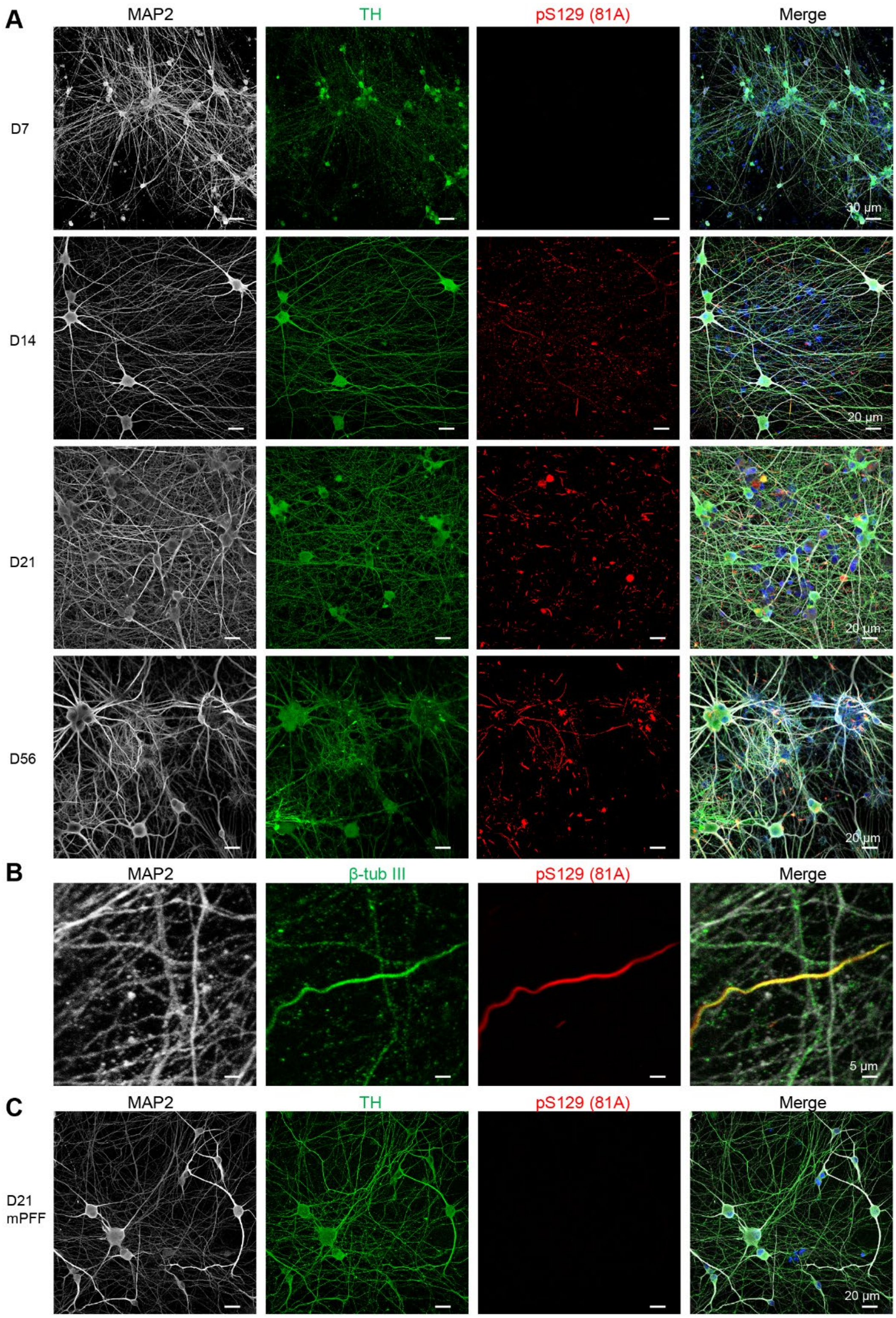

Figure S5. Characterization of aSyn pathology formation in human iPSC-derived dopaminergic neurons over time. (**A**) Individual fluorescence channels corresponding to the images shown in Figure 1J at D14, D21, and D56 following PFF treatment. The pS129 signal (81A antibody), microtubule-associated protein 2 (MAP2), tyrosine hydroxylase (TH), and DAPI nuclear staining are shown separately from the same multichannel acquisitions and identical fields of view. While Figure 1J displays only the pS129 channel, this figure provides the full set of channels for those images. Scale bars = 20 µm. (**B**) pS129 neuritic pathology was colocalized with neurites positively stained by the β-tubulin III antibody but not with MAP2, indicating that the pS129 pathology localized predominantly in axonal neurites rather than dendrites. Scale bars = 20 µm. (**C**) Mouse PFF failed to induce pS129 pathology (81A antibody staining) in iDA 21 days (D21) after PFF treatment. Neurons were labelled with microtubule-associated protein 2 (MAP2) and Tyrosine Hydroxylase (TH) antibodies, while nuclei were counterstained with DAPI. Scale bars = 20 µm.

Supplementary materials

Figure S6

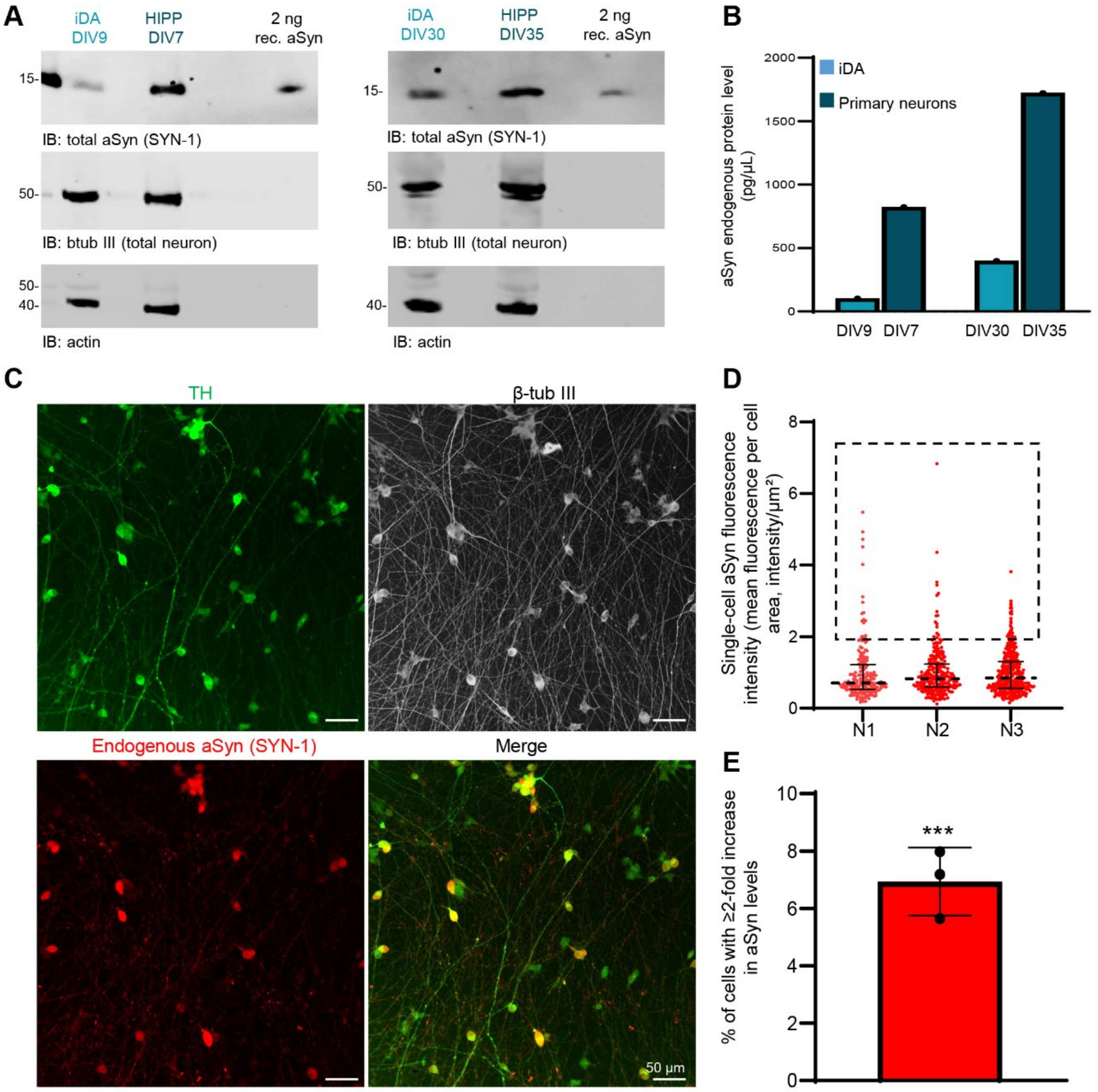

Figure S6. Endogenous aSyn levels in iDA cultures.
(**A–B**) WB analysis comparing endogenous aSyn levels in iDA cultures and hippocampal primary neurons at two key time points: DIV9, when both cultures are treated with aSyn preformed fibrils (PFF) in the seeding model, and DIV30, when initial somatic aggregates begin to appear in iDA cultures following PFF addition. Total aSyn was detected using the SYN-1 antibody. Actin and β-tubulin III were used as loading controls, while TH staining was included to confirm dopaminergic differentiation of iPSC-derived neurons. (**A**) Representative immunoblots show substantially lower aSyn expression in iDA cultures relative to primary neurons. (**B**) Densitometric analysis reveals aSyn concentrations of ~104 pg/μL in iDA cultures at DIV9 versus ~825 pg/μL in primary neurons at DIV7, and ~400 pg/μL in iDA cultures at DIV30 versus ~1723 pg/μL in primary neurons at DIV35. Quantification was performed using the signal intensity of 2 ng of recombinant aSyn protein loaded on the same blot as a reference standard. Data represent mean ± SD from one experiment. (**C-E**) Quantification of endogenous aSyn levels in iDA cultures using high-content imaging analysis to assess single-cell expression. iDA neurons were plated in 96-well plates and fixed at DIV9. Cells were immunostained for total aSyn (SYN-1 antibody), TH, and MAP2 to identify dopaminergic neurons and neuronal processes, respectively (**C**). Data represent mean ± SD from a minimum of three independent biological replicates. For each independent experiment, three wells per condition were imaged, with nine fields of view acquired per well. Each experiment was independently replicated at least three times. For each detected cell, the median gray value of the aSyn fluorescence channel was used as a representative measure of intracellular aSyn levels. Mean fluorescence intensity per cell was normalized by the corresponding cell area and then normalized to the population mean for each independent experiment. The resulting data were visualized as violin plots (**D**). Graph **E** shows the percentage of cells exhibiting a ≥2-fold increase in aSyn levels, derived from the three independent experiments presented in Graph **C**. The quantified values correspond to the regions highlighted by the dashed boxes in Graph **C**. Statistical analyses: ANOVA with Tukey HSD post hoc tests. (**E**) *p < 0.0001. Scale bars = 50 μm.

Supplementary materials

Figure S7

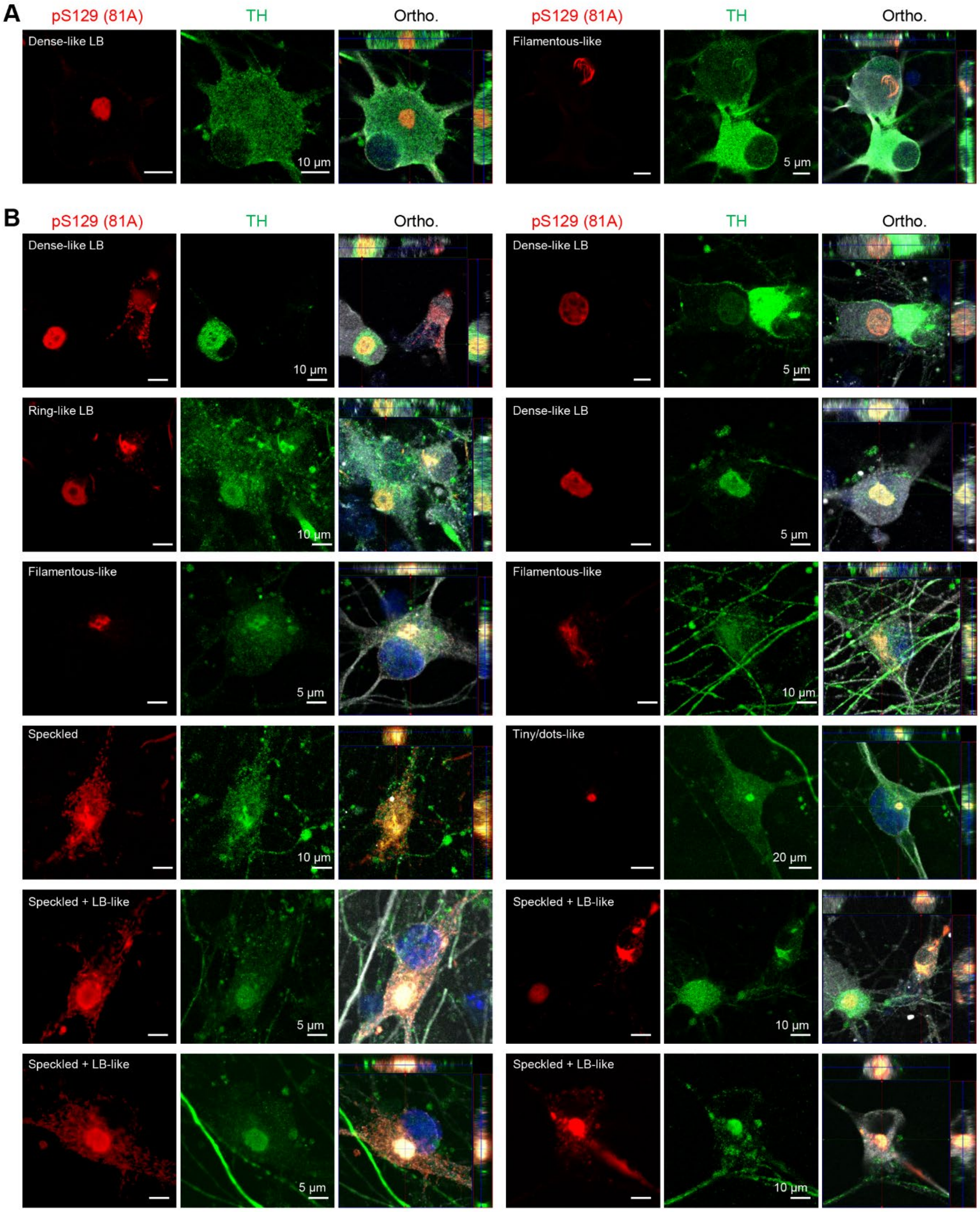

Figure S7. Tyrosine Hydroxylase relocalization in pS129-positive aggregates.
TH immunoreactivity was observed within pS129-positive aggregates (detected using the 81A antibody) at both D21 (**A**) and D56 (**B**). At D21, approximately 10–20% of aggregates showed TH relocalization, increasing to ~20–30% by D56. This relocalization occurred irrespective of aggregate morphology. Notably, TH signal intensity was significantly higher within the seeded aggregates than in the surrounding cytoplasm, which appeared nearly devoid of TH staining, indicating redistribution of TH into the aggregates. Nuclei were counterstained with DAPI. Scale bars = 5 or 10 µm.

Supplementary materials

Figure S8

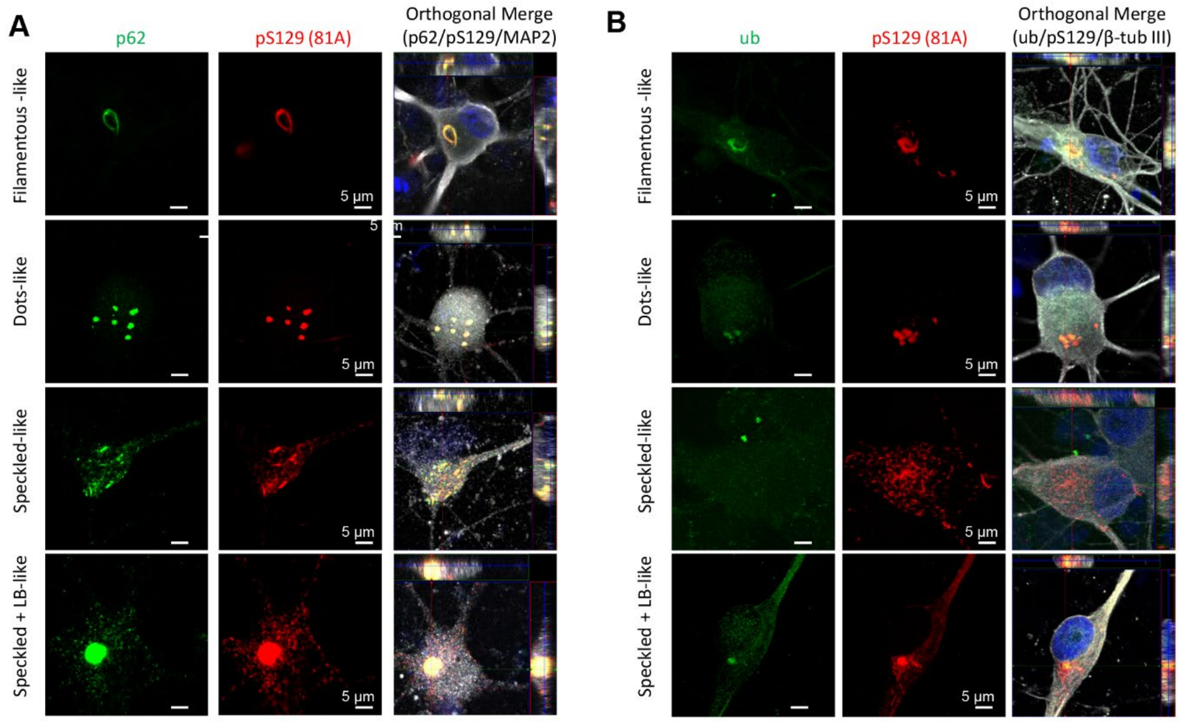

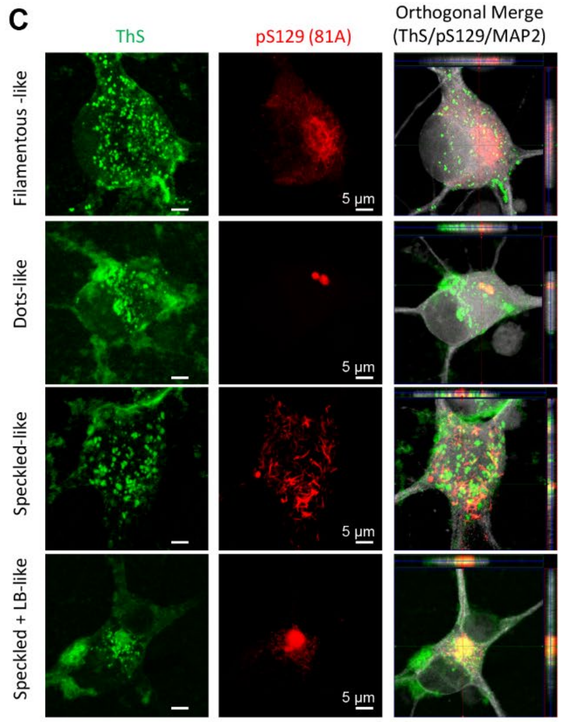

D

Type of aggregates (D56)	p62	ub	ThS
Neuritic aggregates	-	+	+
Filamentous-like Like aggregates	+	+	-
Dots-like aggregates	+	+/-	+/-
Speckled filaments	+	-	-
Ring-like LB	+	+	+
Dense-like LB	+	+	+
Speckled<br>+LB	+<br>+	-<br>+	+<br>+

Figure S8. Seeded aggregates in iDA neurons exhibit LB pathological markers similar to those in human brain pathology. Representative images showing the differential sequestration of p62 (**A**), ub (**B**) and ThS (**C**) with pS129 pathology (81A antibody) in somatic pathology at D56. Neurons were stained with MAP2, while nuclei were counterstained with DAPI. Orthogonal projections (Ortho.). Scale bars = 5 µm. (**D**) Summary table showing the differential sequestration of p62, ubiquitin, and ThS in different types of seeded aggregates at D56 (see additional staining in **Figure 3**). Schematic illustrations were generated using BioRender.com.

Supplementary materials

Figure S9 – Part I

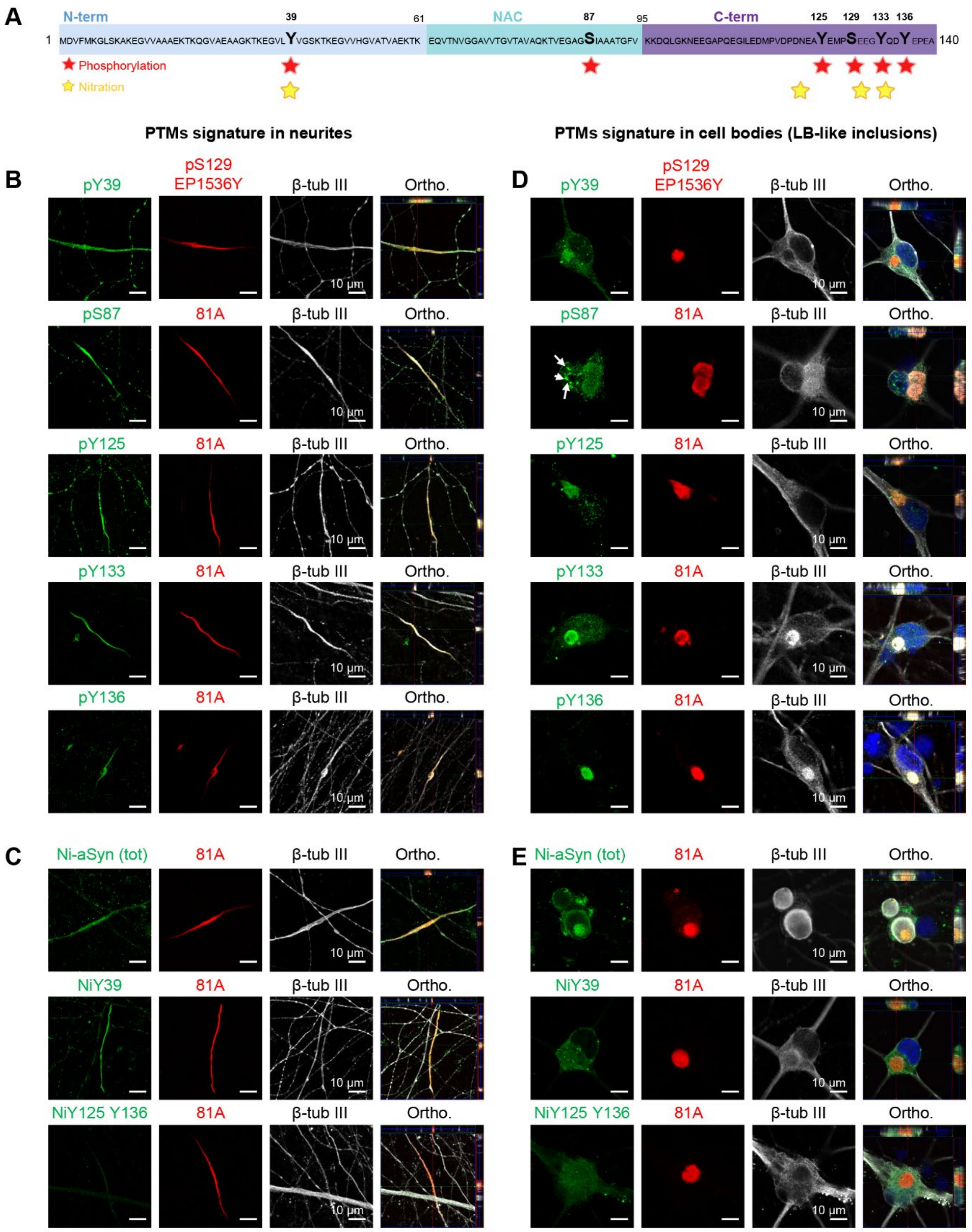

Supplementary materials

Figure S9 – Part II

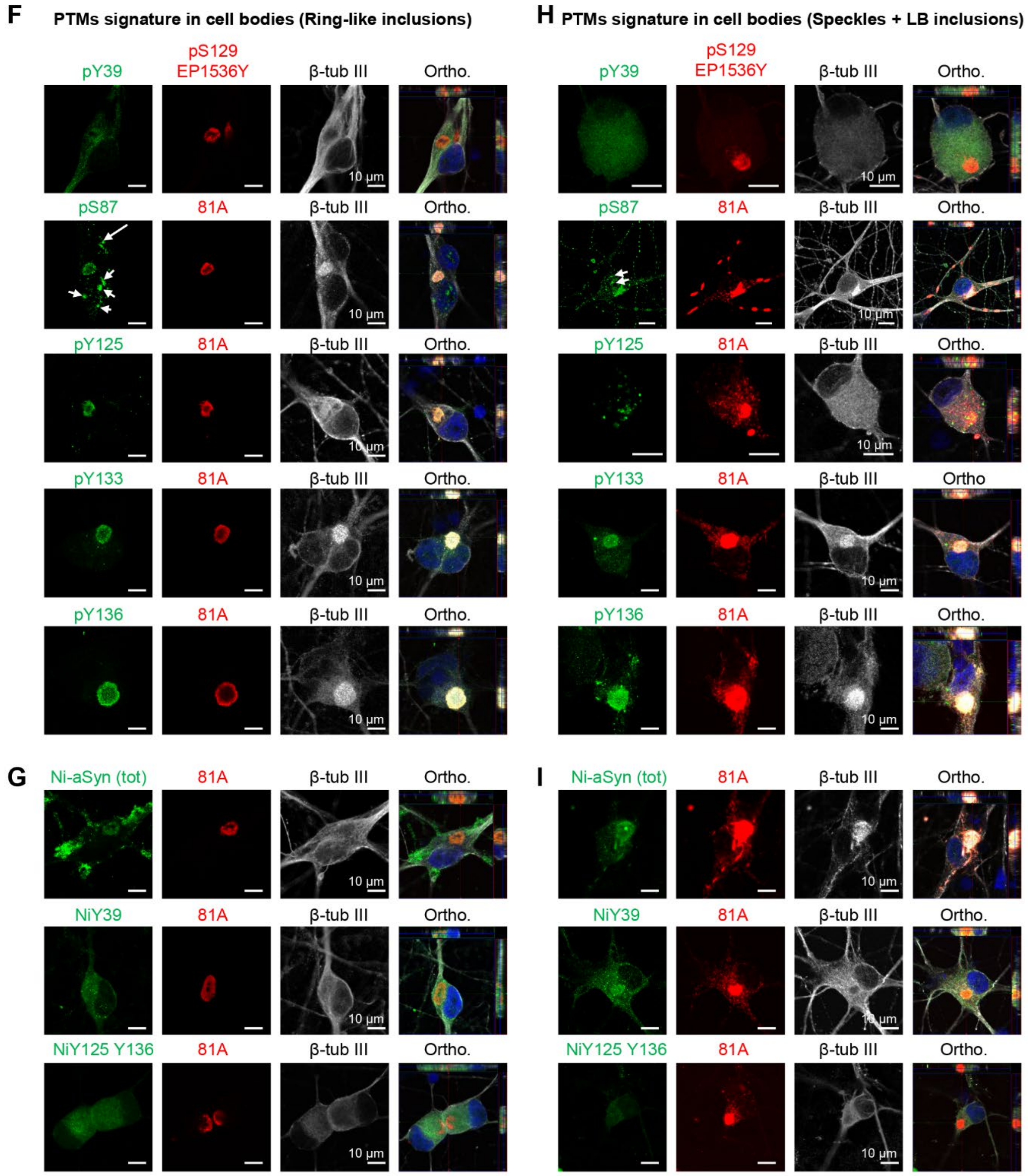

Supplementary materials

Figure S9 - Part III

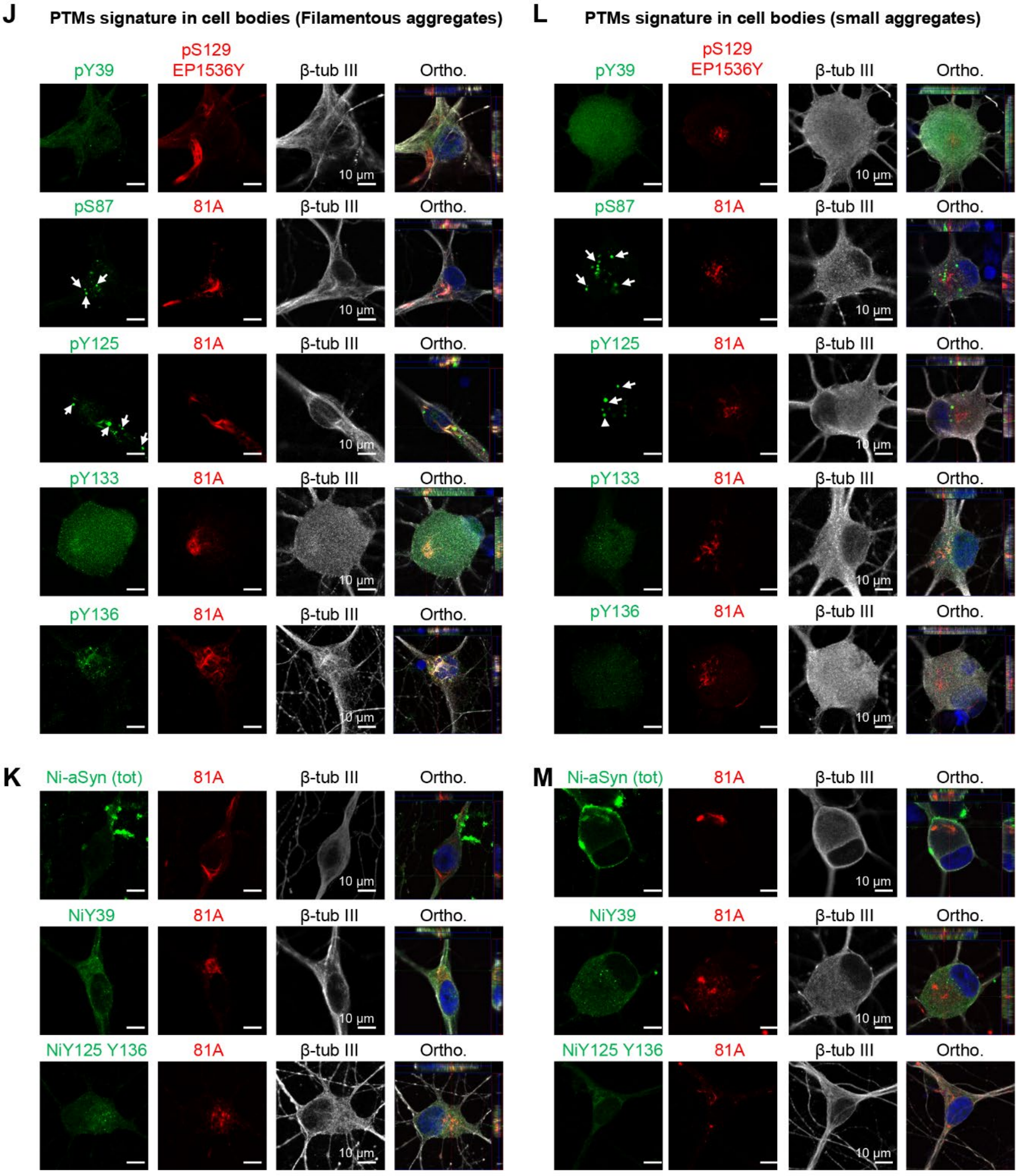

Figure S9. The iDA seeding model replicates the PTM signature associated with human pathology. (**A**) Schematic representation of PTM analyzed in this study. These include phosphorylation at residue Y39, S87, Y125, S129, Y131 and Y136, and nitration at Tyrosine residues (nY39, nY125, nY131, nY136). All of these PTM have been previously identified in aSyn pathology in post-mortem brain tissues from patients with PD and MSA. (**B-M**) Representative images of pS129 pathology at D56 (B-C, neuritic pathology; D-E, dense LB inclusions; **F-G**, ring-like LB inclusions; **H-I**, Speckles and LB-like inclusions; **J-K**, filamentous aggregates and **L-M**, small aggregates) colocalizing with various PTM, including pY39, pS87, pY125, pY131, and pY136 phosphorylation (**B**, **D**, **F**, **H**, **J** and **L**) or with antibodies specific for nitration at Y39 (nY39) or Y125/Y136 (nY125/nY136) (**C**, **E**, **G**, **I**, **K** and **M**). White arrows indicate additional dot-like structures detected by some PTM antibodies, which are not pS129-positive. Neurons were stained with the β-tubulin III antibody, while nuclei were counterstained with DAPI. Orthogonal projections (Ortho.). Scale bars = 10 µm. The merged images shown in Fig. S9 correspond to those presented in Fig. 4 and originate from the same fields of view. Fig. S9 provides both the merged images and the corresponding individual fluorescence channels.

Supplementary materials

Figure S10

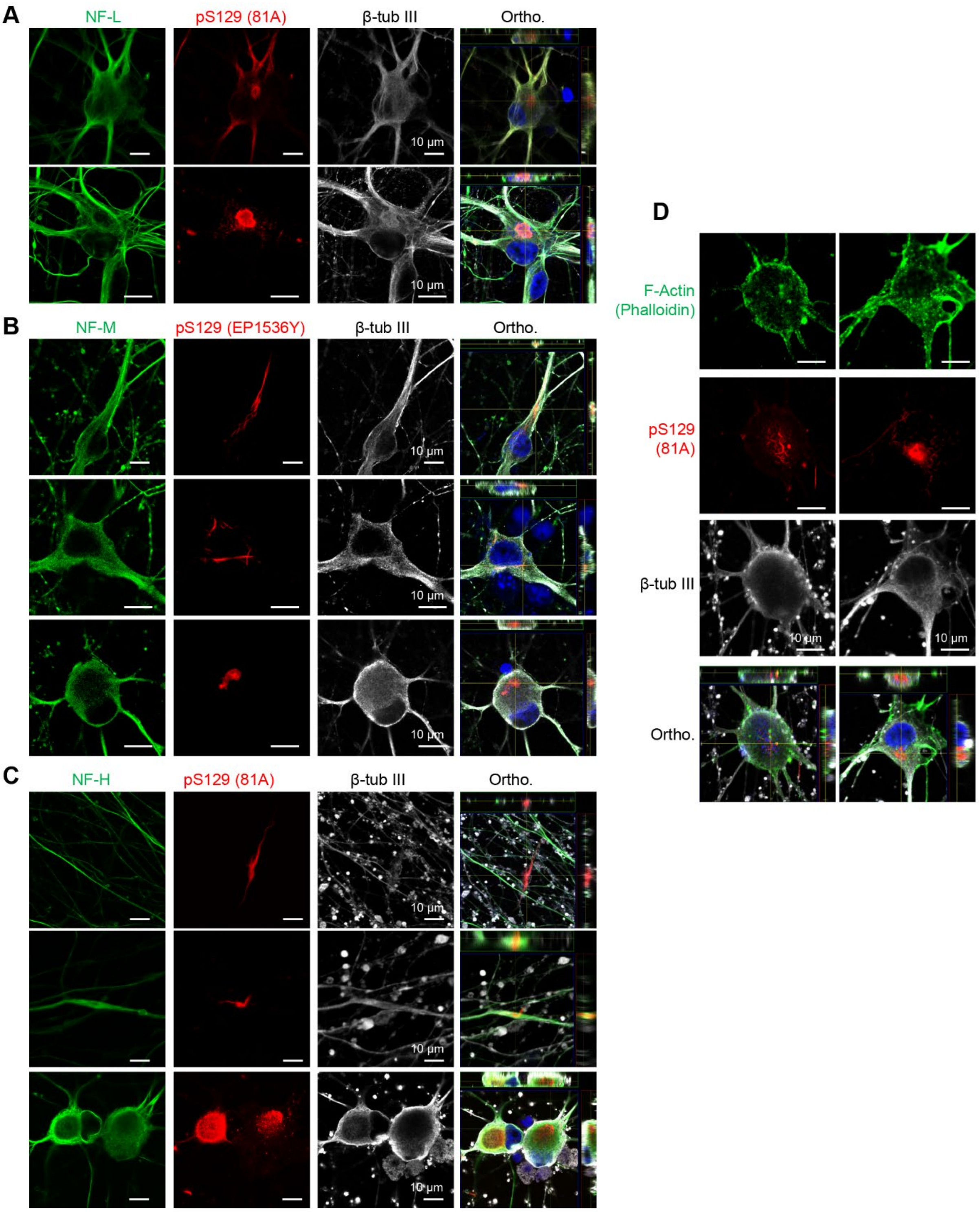

Figure S10. pS129 pathology does not colocalize with major cytoskeletal proteins.
ICC demonstrates the absence of colocalization between pS129 pathology and filamentous cytoskeletal proteins. (**A**) Neurofilament light chain (NF-L, green) does not colocalize with pS129 pathology detected by 81A antibody (red) in somatic or neuritic regions. β-tubulin III (gray) staining highlights neuronal morphology, with orthogonal (Ortho.) projections confirming the lack of overlap. (**B**) Similar results were observed for neurofilament medium chain (NF-M, green), with pS129 detected using EP1536Y antibody (red). (**C**) Neurofilament heavy chain (NF-H, green) also showed no colocalization with pS129 pathology detected by 81A antibody (red). (**D**) Phalloidin staining of F-actin (green) confirms no colocalization with pS129 pathology (81A antibody, red) in neuritic or somatic regions. Neurons were stained with the β-tubulin III antibody, while nuclei were counterstained with DAPI. Orthogonal projections (Ortho.). Scale bars = 10 µm.

Supplementary materials

Figure S11

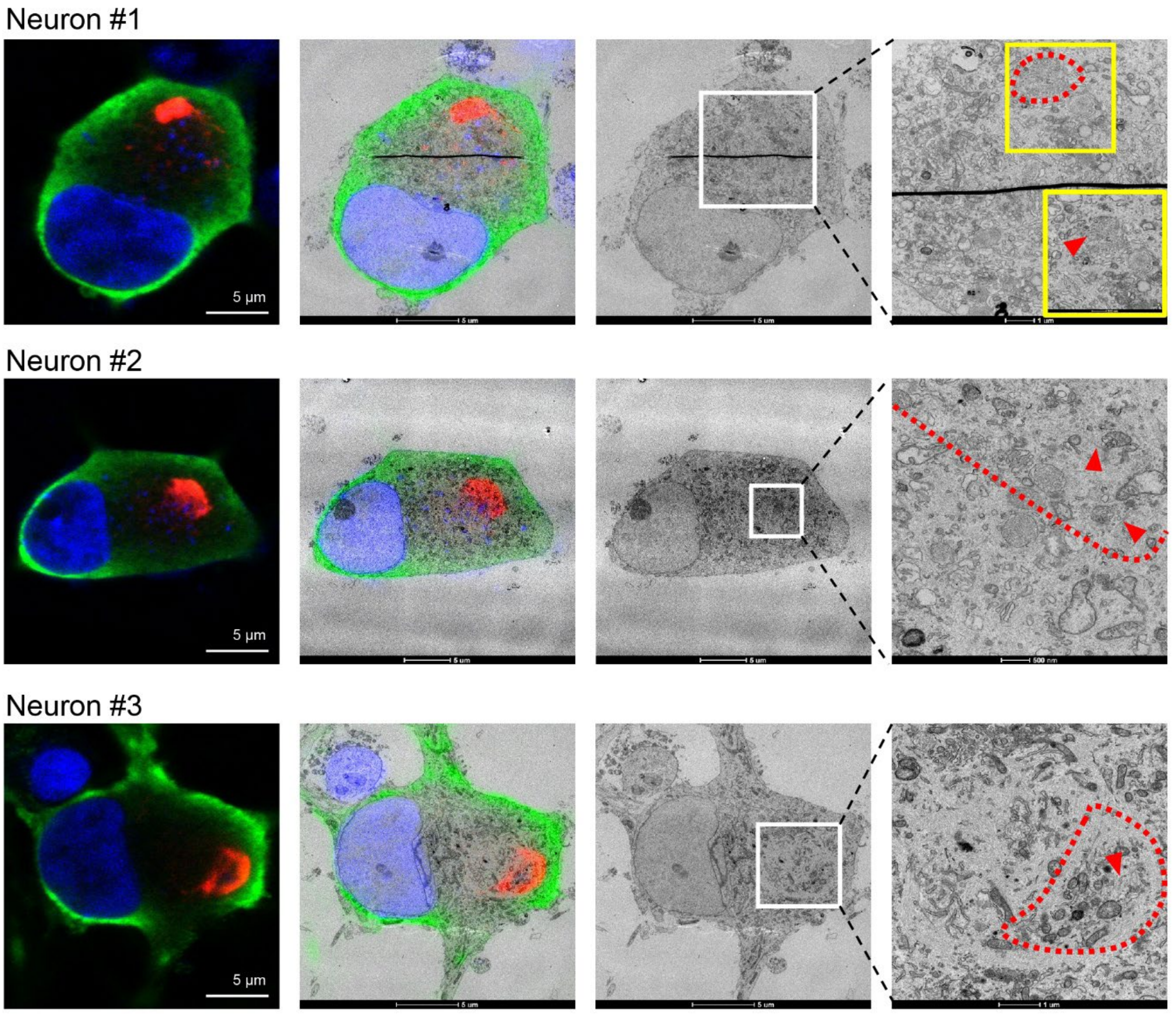

Figure S11. Ultrastructure of dense D21 aggregates surrounded by speckled cytoplasmic staining.
The aSyn-pS129 immunofluorescence profile of the neurons #1, #2 and #3 consists of a dense centre surrounded by a punctate pattern of aSyn-pS129 immunoreactivity. The dense immunostaining colocalizes with a mixture of small vesicles, membranous organelles (notably mitochondria and lysosomes) and thick (13 ± 2.5 nm) fibrils that are randomly oriented (red arrows). The surrounding regions, with a speckled immunopositivity, correlate with thin (8 ± 2nm) filaments that curve throughout the cytosol. Scale bars = 500 nm or 1 and 5 µm.

Supplementary materials

Figure S12 – Part I

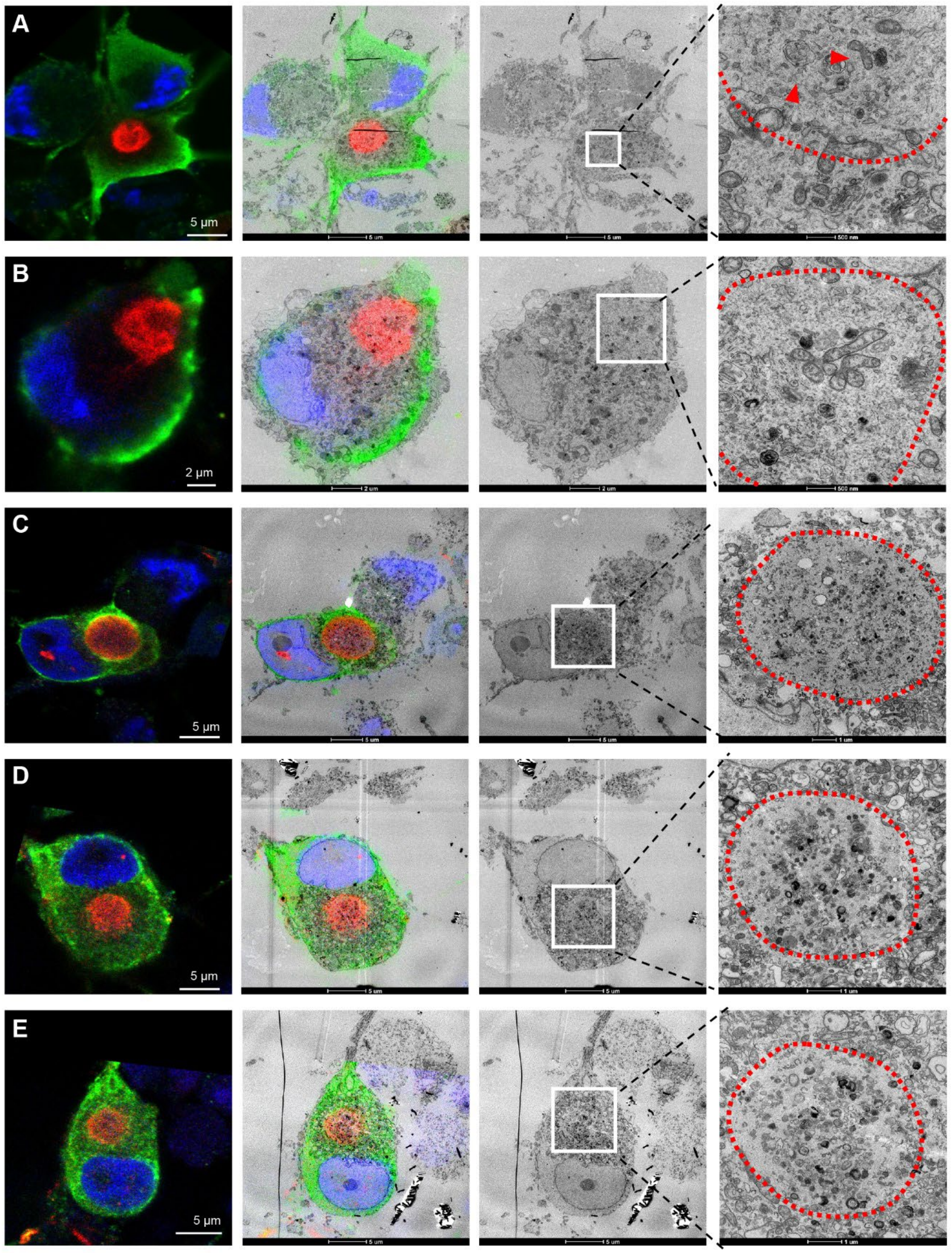

Figure S12 – Part II

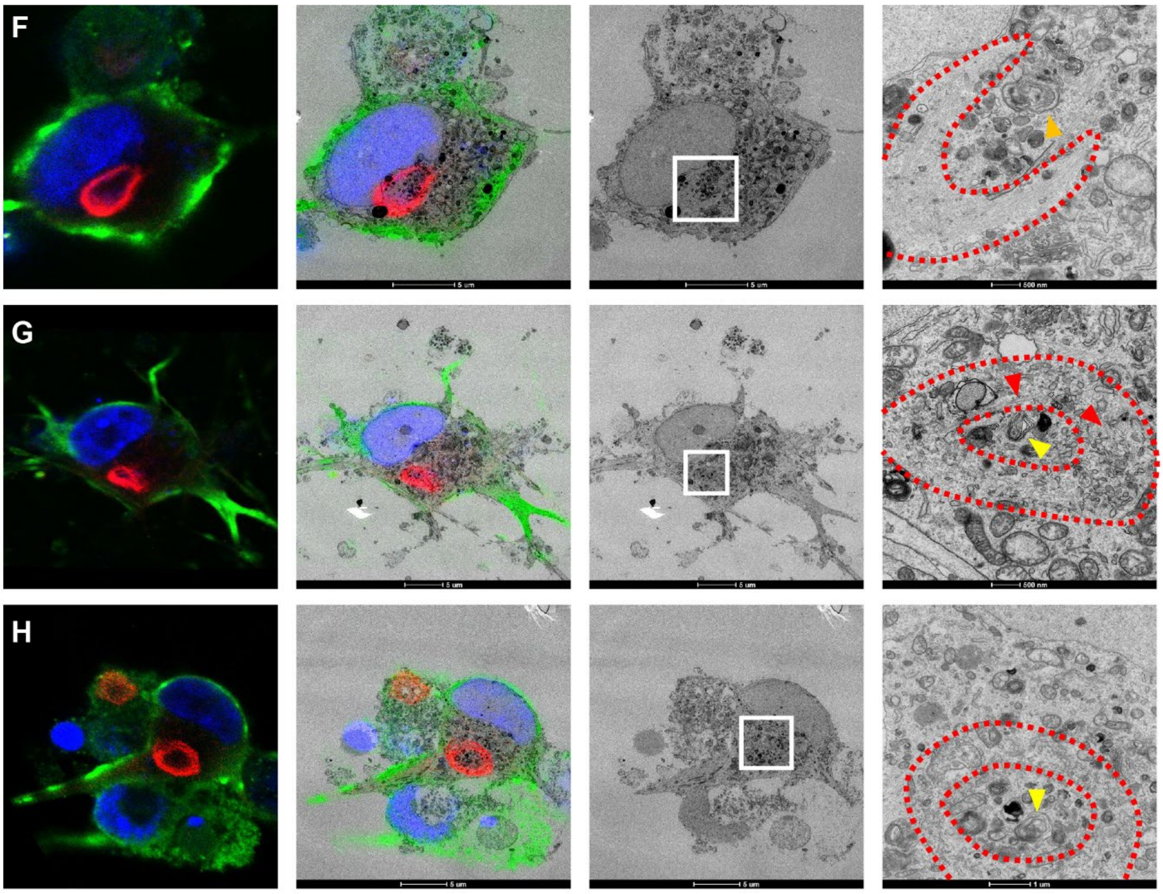

Figure S12. Ultrastructure of the different types of LB at D56.
(**A-E**) Ultrastructure of the dense LB. These aggregates share a membranous ultrastructure rich in small vesicles, a few thick fibrils (13.5 ± 2.5 nm, as indicated by the red arrow in **A**), clustered mitochondria (notably in **B**) and thin filaments (8 ± 2nm, notably in **C**, **D** and **E**). Note that the number of thick fibrils in dense D56 aggregates is lower than in dense D21 aggregates, suggesting degradation of newly formed aSyn fibrils over time. Scale bars = µm. (**F-H**) Ultrastructure of the ring-like LB. The centre of the ring, which is low in aSyn-pS129 immunoreactivity, correlates with the presence of autophagosomes (orange arrow), multilamellar bodies (yellow arrows) and other lipid-rich organelles. In **F**, the ring itself correlates with an ultrastructure that is rich in parallel filaments, whereas in **G** and **H,** the ring is a membranous region consisting of small vesicles, organelles, and thick filaments (13 ± 2.5 nm, red arrows). Scale bars = 500 nm or 1, 2 and 5 µm.

Supplementary materials

Figure S13 – Part I

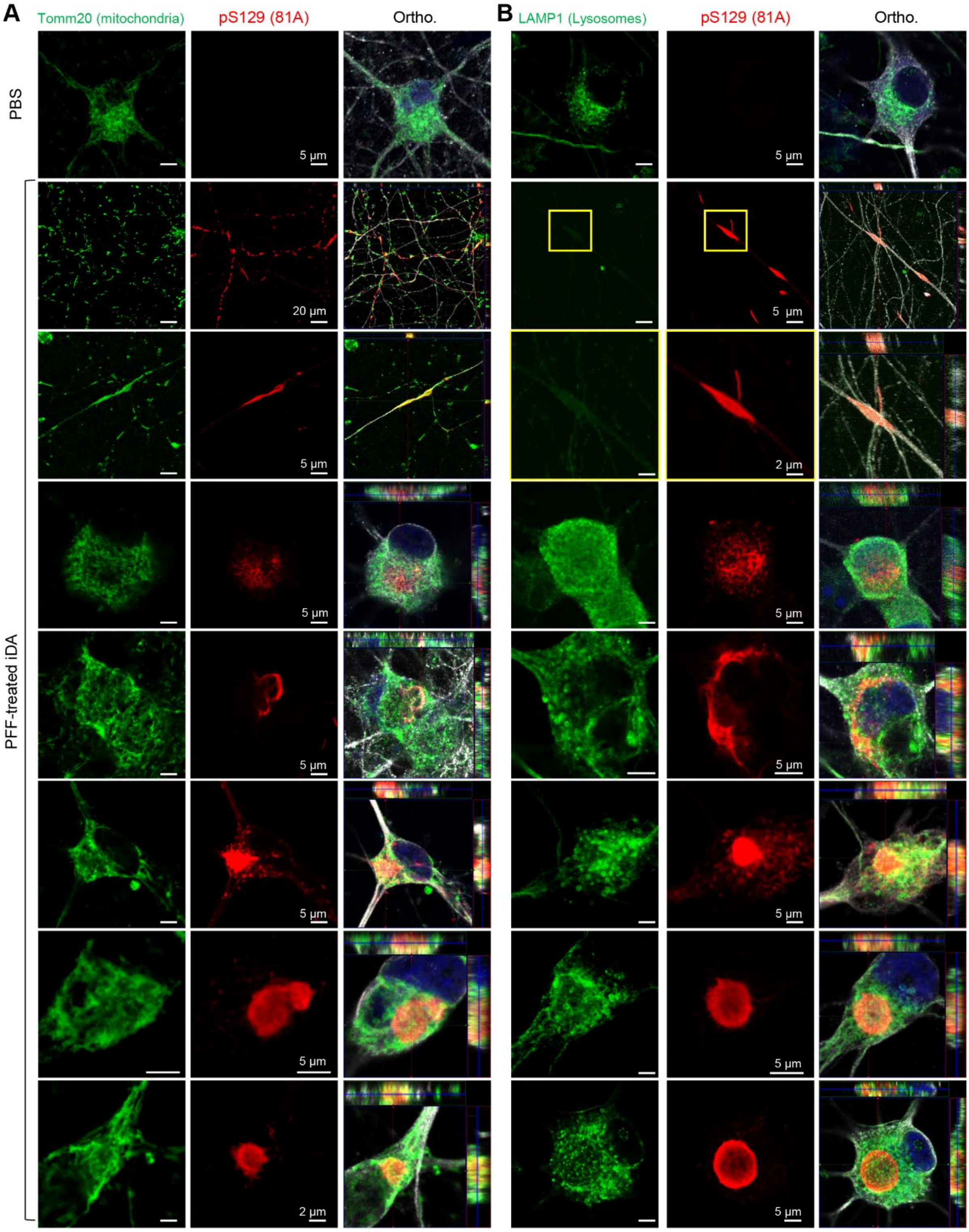

Supplementary materials

Figure S13 – Part II

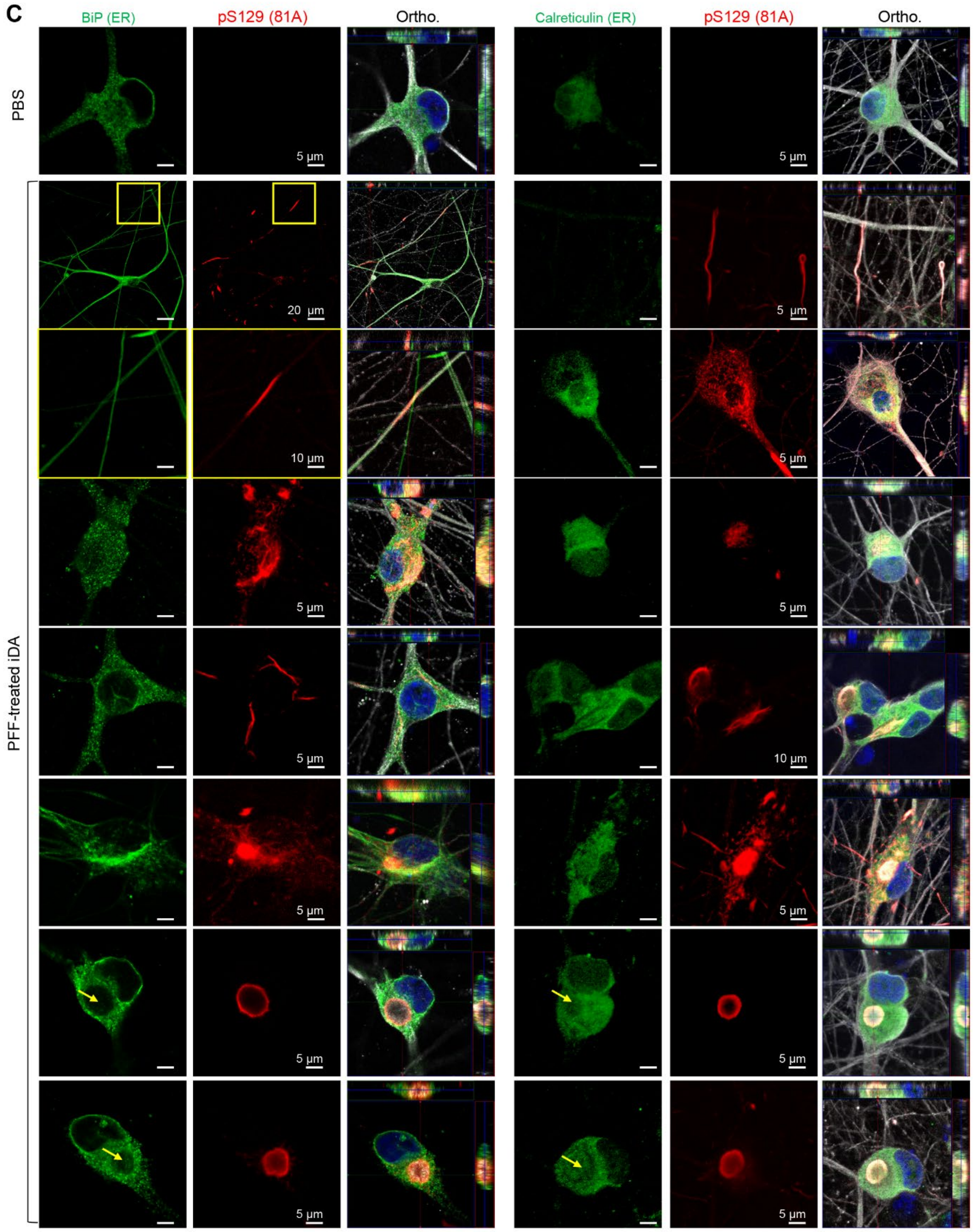

Figure S13 – Part III

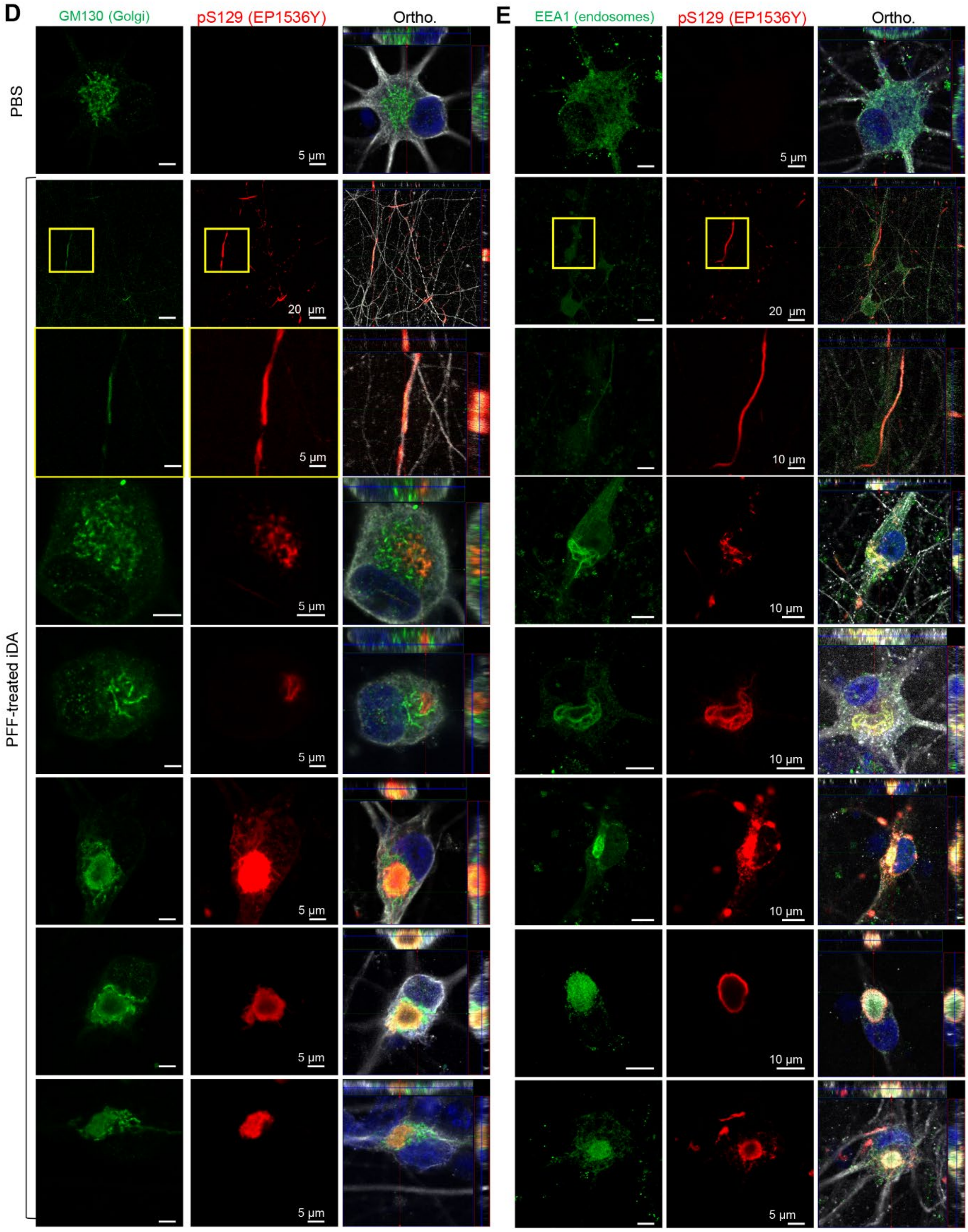

Figure S13. Sequestration of key organelles into pS129-positive aggregates at D56.
(**A-D**) Representative images show pS129 pathology (red, 81A antibody) alongside markers for key organelles: Tomm20 for mitochondria (**A**), LAMP1 for lysosomes (**B**), Bip for the endoplasmic reticulum (ER) (**C**), GM130 for the Golgi apparatus (**D**) and EEA1 for early endosomes (**E**). Neurons were stained with the β-tubulin III antibody, while nuclei were counterstained with DAPI. Orthogonal projections (Ortho.). Scale bars = 5, 10 or 20 µm as indicated for each panel.

Supplementary materials

Figure S14

Categories	Parameters	Published iPSC-derived seeding models	This study
**Neuronal model and differentiation**	Cell type	Cortical neurons *(28, 29, 32, 44)*<br>Dopaminergic neurons *(25, 32, 31, 35, 39, 44, 60, 61)*<br>or mixed neuronal cultures (iNeurons) *(22, 32, 40, 44, 58, 59, 63)*	Isogenic iPSC-derived dopaminergic neurons
	Differentiation strategy	Developmental patterning *(28, 29, 32, 31, 35, 39, 44, 60, 61)*<br>or NGN2-driven programming *(22, 32, 44, 58, 40, 59, 63)*	NGN2-driven programming
	Neuronal population homogeneity	Variable neuronal composition frequently reported *(22, 25, 28, 29, 32, 31, 32, 35, 39, 40, 44, 58, 59, 60, 61, 63)*	Highly homogeneous dopaminergic neuronal population (> 80%)
**Endogenous aSyn context**	Endogenous aSyn protein level quantification	Reported in a limited number of studies *(22, 40, 59)*	Endogenous aSyn protein levels quantified longitudinally by WB using recombinant aSyn standards to determine absolute concentration
	Endogenous aSyn abundance	Prior studies generally reported WB detection without absolute quantification *(22, 59)* or ~200 pg/µL at DIV46 *(40)* or 25 pg/µL at DIV21 *(iCell DopaNeurons, Fujifilm, Filali et al., ADPD2022 poster P538)*	Increased endogenous aSyn levels overtime (~100 pg/µL at DIV9 and 300 pg/µL at DIV30)
**PFF preparation and quality control**	PFF source	Recombinant human aSyn PFFs *(22, 25, 28, 29, 35, 59)* or brain-derived fibrils *(32, 39)*	Recombinant human aSyn PFFs
	Fibrillization protocol	Variable protocols across studies	Standardized fibrillization protocol
	Structural / biochemical QC	Structural/biochemical QC reported in some studies but not systematically (e.g., kinetics of fibrilization *(25, 28)*<br>TEM *(22, 25, 28, 29, 32, 39, 40, 44)*, PFFs stability *(29, 40)*	Systematic TEM characterization pre- and post-sonication with biochemical QC (SDS–PAGE/Coomassie) and ThT β-sheet validation.
	Fibril length distribution post-sonication	≤50 nm *(32)*<br>50-100 nm *(22, 32, 39, 44)*	Controlled fibril length distribution; only post-sonication batches containing 50–100 nm fibrils were selected for experiments.
	Endotoxin removal	Endotoxin-free preparations used in some studies *(44)*	PFFs were not prepared under endotoxin-free conditions; Standard preparation maximizing cellular internalization *(51, 53)*
**Seeding paradigm**	PFF concentration	Broad concentration range across studies<br>PFF concentration:<br>≤1 µg/mL *(32, 40, 44, 56, 59);*<br>2-5 µg/mL *(25, 28, 32, 34, 35, 60);*<br>10-15 µg/mL *(22, 32, 39, 41, 63)*<br>59µg/mL *(29)*<br>In cell lines with aSyn gene multiplication, a minimum of 5 µg/mL and up to 10 µg/mL was used *(32, 39, 63)*	This study employed an optimized concentration (500 nM; ~7 µg/mL)
	Timing of PFF addition	Variable timing of seeding across studies<br>≤DIV7 *(28, 35)*<br>DIV7-DIV14 *(32, 44, 59, 60, 61)*<br>DIV14-DIV21 *(22, 32)*<br>>DIV21 *(25, 29, 39, 40, 60, 61)*	Defined maturation stage optimized (DIV9) prior to seeding
	Duration post-PFF treatment	1-2 weeks *(22, 28, 31, 35, 40, 58, 59, 63)*<br>3-4 weeks *(25, 29, 32, 32, 39, 44)*<br>> 2months *(61)*	Longitudinal follow-up enabling maturation analysis (D14, D21, D40 and D56 post PFF-treatment)
**Pathology induction context**	Requirement for aSyn overexpression	PFF + aSyn overexpression used in several studies *(32, 40, 58)*	No aSyn overexpression
	Requirement for genetic risk factors	PFF seeding performed in genetic risk backgrounds, including A53T *(32, 39, 60)*<br>SNCA triplication *(25, 31, 32, 39, 44, 61, 63)*<br>or other PD-associated variants (LRRK2 *(22)*, GBA *(22)*, GPNMB *(59)*)	No genetic sensitization required
	Requirement for cellular stressors	Additional stressors used to enhance seeding in some studies *(44)*	No stressor treatment required

Figure S14. Key experimental determinants influencing aSyn seeding efficiency in iPSC-derived neuronal models. G. Bieri et al ***(22)***, LRRK2 modifies α-syn pathology and spread in mouse models and human neurons. Acta Neuropathol (2019). Y. Chen et al ***(25)***, Engineering synucleinopathy-resistant human dopaminergic neurons by CRISPR-mediated deletion of the SNCA gene. Eur J Neurosci (2019). J. Gao, et al ***(28)***, Autophagy activation promotes clearance of α-synuclein inclusions in fibril-seeded human neural cells. J Biol Chem (2019). S. Gribaudo et al ***(31)***, Propagation of α-Synuclein Strains within Human Reconstructed Neuronal Network. Stem Cell Reports (2019). A. Iannielli et al ***(32)***, Modeling native and seeded Synuclein aggregation and related cellular dysfunctions in dopaminergic neurons derived by a new set of isogenic iPSC lines with SNCA multiplications. Cell Death Dis (2022). M. S. Kim et al ***(31)***, Advanced human iPSC-based preclinical model for Parkinson's disease with optogenetic alpha-synuclein aggregation. Cell Stem Cell (2023). I. Lam et al ***(32)***, Rapid iPSC inclusionopathy models shed light on formation, consequence, and molecular subtype of α-synuclein inclusions. Neuron (2024). A.-L. Mahul-Mellier et al ***(34)***, Differential role of C-terminal truncations on alpha-synuclein pathology and Lewy body formation NPJ Parkinsons (2025). I. Pediaditakis et al ***(35)***, Modeling alpha-synuclein pathology in a human brain-chip to assess blood-brain barrier disruption. Nat Commun (2021). B. Tanudjojo et al ***(39)***, Phenotypic manifestation of α-synuclein strains derived from Parkinson's disease and multiple system atrophy in human dopaminergic neurons. Nat Commun (2021). C. Vajhøj et al ***(40)***, Establishment of a human induced pluripotent stem cell neuronal model for identification of modulators of A53T α-synuclein levels and aggregation. PLoS One (2021). V. D. Valderhaug et al ***(41)***, Early functional changes associated with alpha-synuclein proteinopathy in engineered human neural networks. Am J Physiol Cell Physiol (2021). A. Bayati et al ***(44)***, Modeling Parkinson's disease pathology in human dopaminergic neurons by sequential exposure to α-synuclein fibrils and proinflammatory cytokines. Nat Neurosci (2024). S. T. Kumar et al ***(51)***, A simple, versatile and robust centrifugation-based filtration protocol for the isolation and quantification of α-synuclein monomers, oligomers and fibrils: Towards improving experimental reproducibility in α-synuclein research. J Neurochem (2020). A. L. Mahul-Mellier et al ***(53)***, The process of Lewy body formation, rather than simply α-synuclein fibrillization, is one of the major drivers of neurodegeneration. Proc Natl Acad Sci (2020). A. Bayati et al ***(56)***, Rapid macropinocytic transfer of α-synuclein to lysosomes. Cell Rep (2022). A. Sanyal et al ***(58)***, Neuronal constitutive endolysosomal perforations enable α-synuclein aggregation by internalized PFFs. J Cell Biol (2025). M. E. Diaz-Ortiz et al ***(59)***, GPNMB confers risk for Parkinson's disease through interaction with α-synuclein (2022). C. Paschou et al ***(60)***, Proteostasis dysregulation in p.A53T-α-Synuclein iPSC-derived astrocytes exacerbates neurodegeneration in a Parkinson's disease model with Lewy-like pathology. bioRxiv (2025). R. Vroman et al ***(61)***, A high-fidelity microfluidic platform reveals retrograde propagation as the main mechanism of α-Synuclein spread in human neurons. NPJ Parkinsons (2025). E. Hallacli et al ***(63)***, The Parkinson's disease protein alpha-synuclein is a modulator of processing bodies and mRNA stability. Cell (2022).

Supplementary materials

Figure S15

Determinant	Parameter	Recommended criteria (present study)
**Endogenous aSyn levels**	Longitudinal quantification	WB. ICC and/or ELISA monitoring across the differentiation timeline
	Minimum endogenous aSyn concentration	≥ 80 pg/μL prior to seeding
**PFF structural and biochemical quality control**	Fibrillization validation	ThT β-sheet confirmation
	Residual species assessment	SDS-PAGE/Coomassie to evaluate remaining monomers/oligomers
	Morphology verification	TEM validation before and after sonication
	Post-sonication PFF length	Length distribution 50-100 nm
**Human aSyn PFF exposure parameters**	Seeding concentration	500 nM (~7 μg/mL), empirically optimized
	Seeding timing	Adjusted to endogenous aSyn level (≥ 80 pg/μL)
	Incubation duration	~3 weeks for neuritic pathology;<br>~8 weeks for mature LB-like inclusions

Figure S15. Key parameters supporting robust seeding efficiency in the iDA model.

Figure S16

Features of the seeding models vs. human pathology		Primary Hippocampal Neurons	iDA	Human pathology
Endogenous level of aSyn		~ 825 pg/µl (DIV7)	~ 84 pg/µl (DIV9)	Not reported
Pathological hallmarks (pS129, p62, ub, ThS)		+	+	+
Neuritic pathology		Dendritic pS129 pathology >>> axonal	Axonal pS129 pathology >>> dendritic	Axonal pS129 pathology >>> dendritic
Somatic pathology	Dots tiny	-	+	+
	Filamentous	-	+	+
	Speckled	-	+	+
	Ring-like LB	+	+	+
	Dense-like LB	+	+	+
	Speckled + LB-like	-	+	+
PTMs signature		-	+	+
Colocalization with organelles		+	+	+

Figure S16. Comparative analysis of aSyn seeding responses in primary hippocampal cultures and iDA models, and their resemblance to human pS129 pathology.
This table summarizes key features of aSyn pathology observed in hippocampal primary neurons and induced dopaminergic (iDA) neurons following seeding, and compares these with the LB pathological hallmarks described in human brain tissue *(14, 76)*. Parameters assessed include: endogenous aSyn levels, neuritic distribution of pS129 pathology (axon vs. dendrite), aggregate morphological spectrum *(18-19)*, PTM signatures, and organelles sequestration *(15, 17, 75, 80, 90, 91).*

Supplementary materials

Caption for Raw Data S1 (excel file).
Source data associated with all figures in the manuscript. For each figure panel, the Excel file includes the raw replicate values underlying the graphs and the number of samples analyzed in each replicate. Data are organized by figure and panel.
